# Estrogen-Functionalized Ru(II) Polypyridyl Complexes Self-Assemble into Aggregates and Exhibit Selective Phototoxicity against Breast Cancer Cells

**DOI:** 10.1021/acs.inorgchem.5c03244

**Published:** 2025-11-05

**Authors:** Sofia Alexandra Tsoni, Timothy Kench, Ramon Vilar, Theodore Lazarides

**Affiliations:** † Department of Chemistry, 37782 Aristotle University of Thessaloniki, Thessaloniki 54124 Greece; ‡ Department of Chemistry, 4615 Imperial College London, White City Campus, London W12 0BZ U.K.

## Abstract

Photodynamic therapy (PDT) constitutes a promising cancer treatment modality in which an administered photosensitizer is excited with visible light to induce the localized generation of cytotoxic reactive oxygen species (ROS) at the tumor site, thus minimizing damage to healthy tissues and avoiding the use of ionizing radiation. However, most photosensitizers lack inherent selectivity for cancer cells, leading to undesirable accumulation in healthy tissues. The readily formed triplet excited states of Ru­(II) polypyridyl complexes, coupled with their kinetic inertness, make them suitable candidates as photosensitizers for PDT. Herein, we report the synthesis of a series of estrogen-functionalized Ru­(II) complexes using click chemistry and investigate their photophysical properties and biological activity. Dynamic light scattering studies, supported by time-resolved luminescence studies, indicate that the complexes self-assemble into micelle-like structures under experimental conditions relevant to biological studies. Our findings reveal that two of these complexes exhibit highly selective light-induced cytotoxicity toward ER+ breast cancer cells compared to estrogen receptor-negative (ER−) cells, by a factor of up to 9.6. Overall, this study highlights the potential of estrogen-conjugated Ru­(II) complexes for targeted PDT, as well as the importance of aggregation in enhancing cellular uptake.

## Graphical abstract

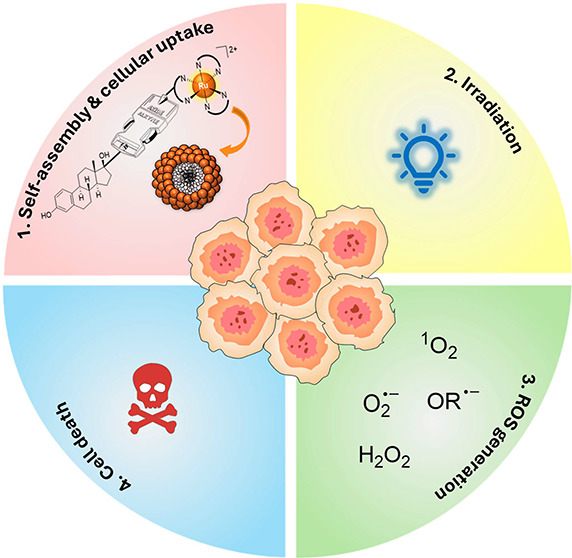

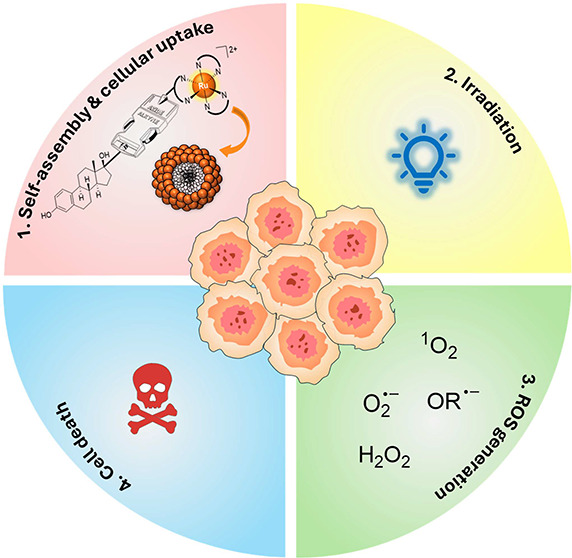

## Introduction

Targeting cancer cells selectively with therapeutic and diagnostic agents remains one of the greatest challenges in cancer treatment.^1^ Photodynamic therapy (PDT) is a cancer treatment modality that employs a photosensitizer (PS) as a prodrug to induce the generation of cytotoxic reactive oxygen species (ROS) within a tumor through activation by light in the visible or near-infrared regions. The photosensitizer is administered either locally or systemically, and after allowing some time for accumulation, the tumor site is irradiated. As a result, the photosensitizer is typically promoted to a triplet excited state, from which it can trigger the generation of ROS (such as singlet oxygen,^1^O_2_) in the irradiated area, ultimately causing cell death.^2^ The localized activation of the photosensitizer within the tumor results in less damage to healthy tissues compared to conventional chemotherapy for cancer treatment.^3^

However, a significant limitation of PDT is that the photosensitizer often lacks inherent selectivity for cancer cells, leading to its accumulation in healthy tissues as well as the tumor. This nonspecific distribution can cause one of PDT’s major side effects: prolonged photosensitivity.^4^ This condition is characterized by an increased sensitivity of healthy tissues to visible light until the photosensitizer is fully cleared from the body.^5^ Consequently, the development of new photosensitizers with active targeting to cancer cells is of great interest.

The [Ru­(bpy)_3_]^2+^ (bpy = 2,2’-bipyridine) core has been widely studied as a photosensitizer due to its well-documented photophysical properties, such as its long-lived triplet metal-to-ligand charge transfer (^3^MLCT) excited state and high ^1^O_2_ production (^1^O_2_ quantum yield (Φ_Δ_) for [Ru­(bpy)_3_]^2+^ in water: Φ_Δ_ = 0.41).^6–8^ Additionally, metal complexes such as these offer advantages over typical organic dyes, including high photostability and the ability to fine-tune their chemical and photophysical properties through ligand functionalization.^9–11^ A prime example of the potential of Ru­(II) polypyridyl complexes in PDT is TLD1433, the first ruthenium-based photosensitizer to progress to clinical trials for treating nonmuscle invasive bladder cancer.^12^ However, TLD1433 is not inherently tumor-selective, prompting efforts to develop tumor-selective analogues.^13^ One of the most promising strategies for achieving this selectivity is bioconjugation, in which the photosensitizer is linked to biomolecules that can specifically target cancer cells such as antibodies. Previous studies have shown promising results by leveraging bioconjugation on Ru­(II) complexes.^14^ In most cases, these complexes have been functionalized with DNA aptamers and nucleotides^15–18^ or proteins, peptides, and amino acids,^19–24^ with only relatively few examples involving functionalization with other biomolecules, such as vitamins, lipids, sugars, and carbohydrates.^14,25–28^

In recent years, significant efforts have focused on functionalizing Ru­(II) polypyridyl complexes with small biomolecules for targeted PDT. For example, one small biomolecule that has been utilized for this purpose is biotin;^29–32^ Li et al. developed a tumor-targeting biotin-functionalized Ru­(II) photosensitizer activated by two-photon excitation,^33^ while Oliveira et al. synthesized a biotin-conjugated complex that was cytotoxic to cancer cells overexpressing biotin receptors.^34^ Gasser et al. explored a different avenue by synthesizing a biotin-functionalized complex that self-assembled into nanoparticles in biological media.^35^ Other small biomolecules that have been utilized for this purpose include various sugars, lipids, and folic acid.^25,26,36,37^ To date, there are limited examples of Ru­(II) polypyridyl complexes designed for estrogen receptor (ER) targeting.^38,39^ In this context, Lo et al. have previously explored the conjugation of ethinyl estradiol to Ru­(II) polypyridyl complexes for the development of luminescent cellular probes and found that two of their complexes exhibited binding affinities to the estrogen receptor alpha (ERα) that are lower than the unmodified estradiol but comparable to other metal complexes functionalized with estradiol.^40^ However, the phototoxicity and selectivity of these complexes between estrogen receptor-positive (ER+) and estrogen receptor-negative (ER−) cells were not explored. Additionally, Peng et al. utilized tamoxifen as an ER-targeting unit and qualitatively demonstrated increased cellular uptake and photodynamic therapeutic efficacy in ER+ cancer cells compared to its tamoxifen-free counterpart.^41^ These studies suggest that incorporating ER-targeting moieties into Ru­(II) polypyridyl complexes could be a promising approach to improving selectivity for ER+ cancer cells. Further research on steroid-functionalized Ru­(II) complexes was conducted by Bonnet and coworkers. Their studies explored the behavior of steroid-functionalized Ru­(II) complexes where the steroid moiety was directly coordinated to the metal core via a sulfur atom.^37,42^ In the first study, they achieved interaction of the metal complex with a biomimetic lipid bilayer built by negatively charged lipids, whereas in the second study, they presented two different modes of action for the complex based on its concentration and incubation time. At concentrations exceeding 3.5 μM, the complexes formed aggregates, leading to detergent-like behavior. Conversely, at lower concentrations, the complexes remained in their monomeric form, and thus, their lipophilic tail was inserted into the cell membrane. The functionalization of transition metal complexes with estrogen moieties remains an area of active research, particularly for developing ER-targeted anticancer therapies. While Ru­(II) complexes have demonstrated potential as ER-targeting photosensitizers, other metal centers—most notably Pt­(II)—have also been investigated for estrogen conjugation.^43–45^ Nevertheless, further studies are necessary to determine whether the observed selectivity is truly mediated by ER interactions.^46^

In this work, we selected three different estrogen derivatives, namely, ethinyl estradiol, mestranol, and quinestrol, as targeting groups for conjugation with the Ru­(II) polypyridyl core for potential use as ER-targeted photosensitizers. This selection is based on the fact that estrogen receptors are overexpressed in several types of cancers, such as breast cancer.^47^ We employed click chemistry to functionalize the ligands, as it is a facile and efficient approach to bioconjugation (Scheme 1a).^48^ We synthesized a series of six new luminescent Ru­(II) complexes with the general formula [Ru­(**L-2**)_2_(**L-1**)]^2+^, where **L-2** is an ancillary polypyridine ligand and **L-1** is a polypyridine estrogen-conjugated ligand (complexes **2**–**7**, Scheme 1b). Alongside these, a control complex lacking the estrogen moiety but maintaining the same general structure was synthesized (complex **1**, Scheme 1b). The photophysical and biological properties of the complexes were investigated, leading to the identification of two of the complexes as the most promising candidates for targeted PDT against estrogen-receptor-positive breast cancer cells.

**Scheme 1:**
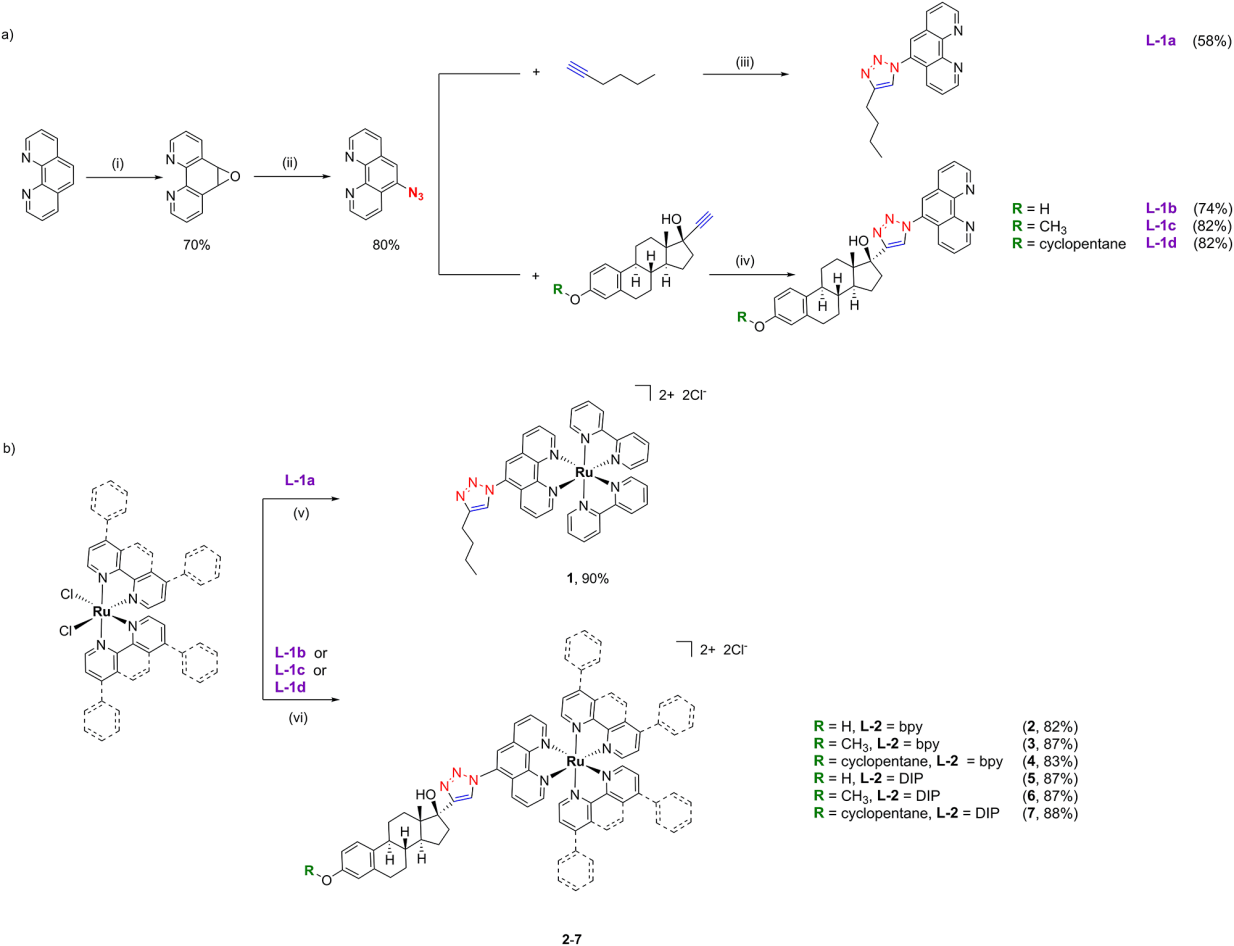
Compounds Studied in this Work^a^ ^b^

a General route for the synthesis of ligands.
b Complexation reactions. Reagents and conditions: (i) Bleach, pH = 8–9, 18 °C, 1 h, (ii) NaN_3_, MeOH/H_2_O, reflux, 24 h, (iii) CuSO_4_, sodium ascorbate, DCM/H_2_O, RT, 4 h, (iv) CuSO_4_, sodium ascorbate, t-butanol/H_2_O, reflux, 12 h for L-1b and CuSO_4_, sodium ascorbate, DCM/H_2_O, RT, 4 h for L-1c and L-1d, (v) and (vi) EtOH/H_2_O, reflux, 12 h.

## Results and Discussion

### Synthesis and Characterization

We employed the “click then chelate” approach to synthesize the Ru­(II) polypyridyl complexes; thus, the estrogen-conjugated ligands were synthesized first, followed by their coordination to the Ru­(II) core. 5-Azido-1,10-phenanthroline was reacted with substrates bearing a terminal alkyne functionality through a Cu­(I)-catalyzed azide–alkyne cycloaddition (CuAAC) reaction, yielding the desired functionalized phenanthroline ligands (Scheme 1a). During this step, an excess of the copper catalyst, which forms in situ by the reduction of Cu­(II) to Cu­(I) by sodium ascorbate,^49^ was required, likely due to the limited reactivity of the aromatic azide combined with the reduced availability of the Cu­(I) catalyst because of its tendency to form bis-phenanthroline complexes.^50,51^ The removal of the excess Cu­(II) cations was ensured by multiple extractions with an ethylenediaminetetraacetic acid (EDTA) solution (0.01 M), as EDTA is an efficient chelator for Cu­(II) ions.^52^ 1-Hexyne was used to synthesize the nonestrogen-functionalized control complex (**L-1a**), whereas ethinyl estradiol, mestranol, and quinestrol were used to synthesize the estrogen-functionalized counterparts (**L-1b**–**L-1d**).

Based on the ancillary ligands, two types of complexes were synthesized. In the first case, we used bpy as the ancillary ligand, whereas in the second case, we used 4,7-diphenyl-1,10-phenanthroline (DIP). All final complexes were obtained by refluxing the corresponding reactants in a 1:1 EtOH/H_2_O mixture overnight. Complexes **1**–**4** were obtained by the reaction of ligands **L-1a**–**L-1d** with *cis*-[Ru­(bpy)_2_Cl_2_], and similarly, complexes **5**, **6**, and **7** were obtained by reacting ligands **L-1b**–**L-1d** with *cis*-[Ru­(DIP)_2_Cl_2_]. All complexes were purified by silica column chromatography using a gradient of MeCN/KNO_3(aq)_ (0.05M) as a solvent system and were converted to chloride salts using an ion-exchange resin to improve their aqueous solubility and biocompatibility.

### Lipophilicity Assessment

The lipophilicity of a compound provides an indication of its likelihood to be cell-permeable. Therefore, we determined the partition coefficient (logP) of the new Ru­(II) complexes between octanol and Tris-HCl buffer using the well-established shake-flask method^53^ (Figure 1a). For comparison, logP values of [Ru­(bpy)_3_]­Cl_2_, estradiol, and ethinyl estradiol are also provided in Figure 1a.^40^ As anticipated, all of the estrogen-functionalized complexes (**2**–**7**) exhibit greater lipophilicity than the control complex **1** and [Ru­(bpy)_3_]­Cl_2_. Complexes **2**–**4**, which feature bpy as the ancillary ligand, display logP values indicative of greater hydrophilicity in comparison to complexes **5**–**7**, which feature DIP as the ancillary ligand. The logP value of the control complex **1** aligns more closely with that of [Ru­(bpy)_3_]­Cl_2_, whereas the incorporation of an estrogen moiety increases the logP value, confirming that estrogen conjugation enhances the complex’s overall lipophilicity. Complexes **2** and **3** maintain negative logP values, with complex **2** being more hydrophilic than complex **3**, likely due to the presence of the hydroxyl group in place of the methoxy group. The incorporation of the cyclopentyl ring in complex **4** further increases the lipophilicity, resulting in a slightly positive logP value. Following this trend, complexes **5**–**7** exhibit positive logP values, confirming their predominantly lipophilic nature. Notably, all synthesized complexes are less lipophilic than estradiol and ethinyl estradiol, likely due to their 2+ charge.^40^ Overall, the logP values obtained indicate that the lipophilicity of the complexes can be modulated by altering the estrogen moiety and ancillary ligand.

**Figure 1:**
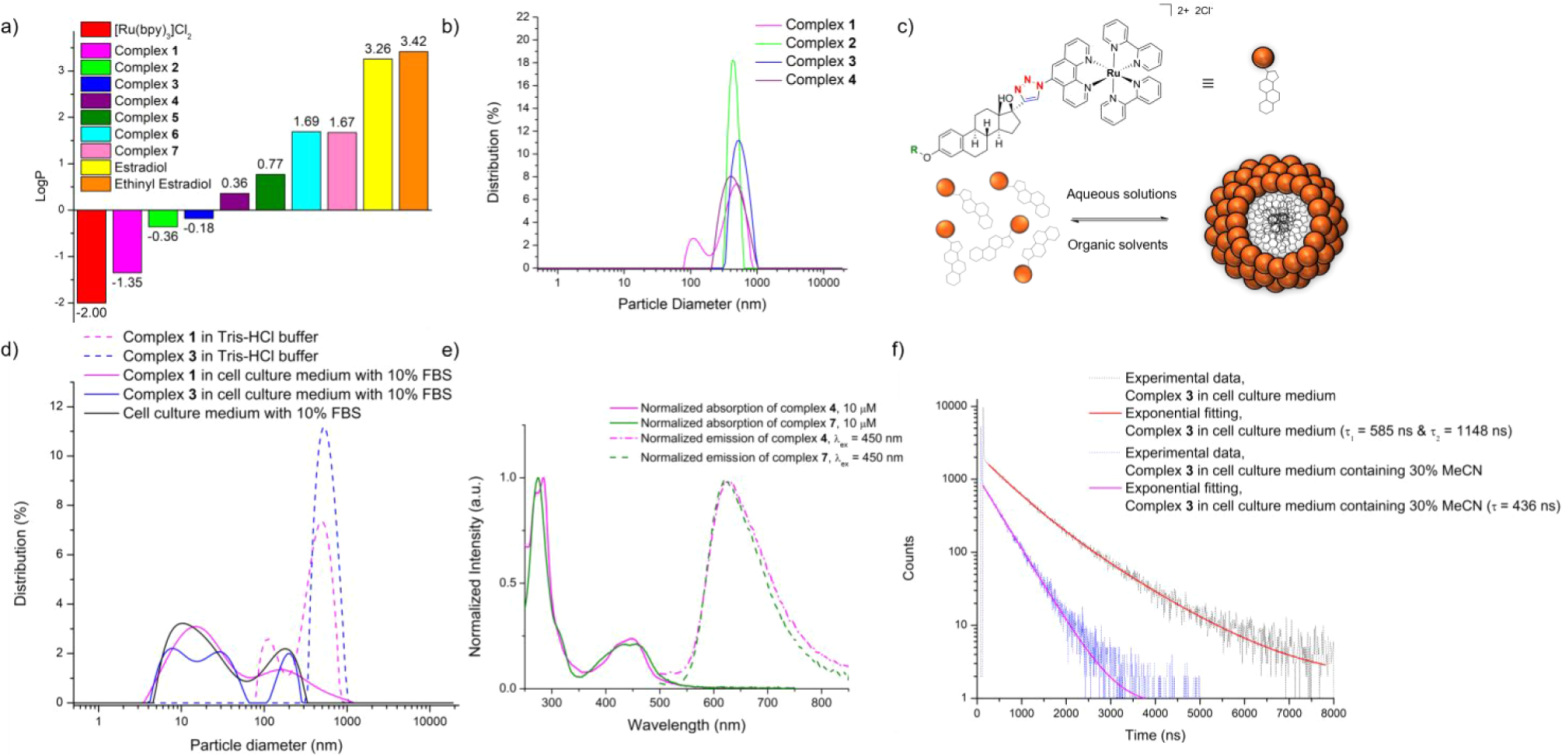
Solubility, aggregation, and photophysical properties of the synthesized complexes. (a) Partition coefficients of complexes **1**–**7** (50 μΜ) between octanol and Tris-HCl buffer (0.05 M, pH 7.4) phases. (b) DLS data: particle size distribution by intensity for complexes **1**–**4** (20 μM) in Tris-HCl buffer. (c) Schematic representation of the postulated aggregation behavior of complexes **1**–**4**. (d) DLS data: comparison of particle size distribution by intensity for complexes **1** and **3** (20 μΜ) in cell culture medium containing 10% FBS and in Tris-HCl buffer. (e) Normalized absorption (solid lines) and emission spectra (dashed lines) of complex **4** (pink, 10 μΜ) and **7** (green, 10 μΜ) in MeCN. (f) Experimental decay data and exponential fittings of complex **3** in pure cell culture medium containing 10% FBS and in a mixture of 30% MeCN and 70% cell culture medium containing 10% FBS.

### Aggregation Studies

Given the amphiphilic nature of the complexes, with the functionalized phenanthroline ligand serving as a lipophilic tail and the Ru­(II) core serving as a hydrophilic headgroup, it was of interest to investigate the potential tendency of the complexes to form micelle-like aggregates in aqueous solutions. Indeed, dynamic light scattering (DLS) analysis for complexes **1**–**4** in Tris-HCl buffer solutions (20 μM) revealed the presence of aggregates (Figure 1b). Our focus was to investigate the aggregation behavior in a purely aqueous environment, without the use of cosolvents such as DMSO. Since complexes **5**–**7** could not be solubilized under these conditions, they were not included in the DLS analysis.

Size distribution analysis indicates that the tested complexes self-assemble into large aggregates in aqueous solution. As shown in Figure 1b, complexes **2**–**4** predominantly form aggregates larger than 200 nm, whereas complex **1** exhibits a broader polydispersity profile, including a population of smaller aggregates around 100 nm. When [Ru­(bpy)_3_]­Cl_2_ was analyzed under the same conditions, no visible aggregates were observed. The aggregation behavior of the complexes could be attributed to their amphiphilic nature, where the functionalized phenanthroline ligands account for the hydrophobic part of the molecule and the Ru­(II) core accounts for the hydrophilic part. The data obtained support our hypothesis that the complexes self-organize into micelle-like aggregates, where the hydrophobic moiety is sheltered by the hydrophilic one in aqueous solutions (Figure 1c). This behavior is in agreement with the literature, where the presence of one polypyridine ligand modified with a lipophilic tail results in an overall amphiphilic complex and the formation of self-assembled supramolecular structures, often referred to as metallosurfactants.^35,56–60^

To assess whether the aggregation behavior observed in buffer is maintained under conditions closer to those used for cell studies, we performed DLS measurements in cell culture medium supplemented with 10% fetal bovine serum (FBS) (Figure S53b).^61,62^ As a control, pure complex-free cell culture medium with 10% FBS was analyzed and, as expected, displayed two peaks attributable to its protein composition.^62^ Interestingly, the peaks in FBS-containing medium for complexes **1**–**4** were different from those observed in Tris-HCl buffer at the same complex concentration (Figure 1d), something that is also reflected in our time-resolved emission studies (*vide infra*). Instead, the complex-containing samples exhibited altered DLS profiles relative to the complex-free control, suggesting that interactions with serum proteins promote the formation of smaller assemblies. For clarity, Figure 1d compares the profiles of complexes **1** and **3** in Tris-HCl buffer versus the FBS-containing medium. While both complexes show altered distributions in the presence of FBS, complex **3** exhibits more pronounced changes, as evidenced by the reshaping of the distribution peak relative to the complex-free control. This trend was consistently observed across all estrogen-functionalized complexes (Figure S53b). Especially for complexes **3** and **4**, a substantial fraction of aggregates are below 200 nm, a size range generally considered favorable for drug delivery applications due to enhanced cellular uptake via endocytosis,^63–65^ as well as the enhanced permeability and retention (EPR) effect within tumor tissues.^66–68^

### Photophysical Properties

We studied the absorption and emission properties of the complexes in different solvents including MeCN, MeOH, and Tris-HCl aqueous buffer (0.05 M, pH 7.4). Due to solubility limitations, only complexes **1**–**4** were studied in Tris-HCl aqueous buffer. Indicatively, the comparative UV–vis absorption and emission spectra of complexes **4** and **7** in MeCN at 298 K are presented in Figure 1e. All compounds exhibited strong absorption bands at ca. 250–280 nm, corresponding to fully allowed π–π* transitions of the polypyridyl ligands, along with characteristic bands in the range of 410–470 nm, corresponding to spin-allowed metal-to-ligand charge transfer (^1^MLCT) transitions.^69,70^

Upon excitation at the ^1^MLCT absorption band, all complexes exhibited a broad emission band peaking at approximately 630 nm in fluid solutions under ambient conditions, attributed to a triplet metal-to-ligand charge transfer (^3^MLCT) state, as anticipated for complexes based on the [Ru­(bpy)_3_]^2+^ core.^71^ Similarly, all complexes exhibited a vibronically resolved peak with a maximum at ca. 580 nm in EtOH/MeOH 4:1 glass at 77 K (Figure S49). The photophysical data are summarized in Tables 1 and 2, and all measurements were conducted in air-equilibrated solvents.

**Table 1:** Photophysical Data of Complexes 1–7

Compound	Medium	λ_abs_ (nm)	ε (M^–1^ cm^–1^)	λ_em_ ^a^ (nm)	Φ (%)	Compound	Medium	λ_abs_ (nm)	ε (M^–1^ cm^–1^)	λ_em_ ^a^ (nm)	Φ (%)
Complex **1**	MeCN (298 K)	450	1.2 × 10^4^	625	2.1	Complex **5**	MeCN (298 K)	457	2.4 × 10^4^	625	2.0
	buffer^b^ (298 K)	449	1.1 × 10^4^	635	5.6		MeOH (298 K)	460	2.2 × 10^4^	620	2.5
	MeOH (298 K)	-	-	-	-		glass^c^ (77 K)	-	-	580 (max), 635	-
	glass^c^ (77 K)	-	-	580 (max), 630	-		buffer (298 K)^b^	-	-	-	-
Complex **2**	MeCN (298 K)	448	9.0 × 10^3^	625	1.7	Complex **6**	MeCN (298)	456	2.5 × 10^4^	625	2.4
	buffer^b^ (298 K)	450	1.0 × 10^4^	635	5.5		MeOH (298)	460	2.3 × 10^4^	620	2.6
	MeOH (298 K)	-	-	-	-		glass (77^c^ K)	-	-	580 (max), 635	-
	glass^c^ (77 K)	-	-	580 (max), 630	-		buffer (298^b^ K)	-	-	-	-
Complex **3**	MeCN (298 K)	450	1.0 × 10^4^	625	2.9	Complex **7**	MeCN (298 K)	458	2.2 × 10^4^	625	2.9
	buffer^b^ (298 K)	450	8.0 × 10^3^	635	6.2		MeOH (298 K)	453	2.6 × 10^4^	620	2.4
	MeOH (298 K)	-	-	-	-		glass^c^ (77 K)	-	-	580 (max), 635	-
	glass (77^c^ K)	-	-	580 (max), 630	-		buffer (298^b^ K)	-	-	-	-
Complex **4**	MeCN (298 K)	446	1.4 × 10^4^	625	2.0	[Ru(bpy)_3_] Cl_2_	MeCN (298 K)	-	-	-	1.6 ^54^
	buffer (298^b^ K)	451	1.4 × 10^4^	635	6.6		buffer (298^c^ K)	453	-	630	-
	MeOH (298 K)	-	-	-	-		H_2_O	-	-	-	4.9^55^
	glass (77^c^ K)	-	-	580 (max), 630	-		glass^c^ (77 K)	-	-	580 (max), 630	-

a Excitation wavelength = 450 nm.

b Tris-HCl buffer (0.05 M, pH = 7.4).

c EtOH/MeOH (4:1 v/v).

**Table 2:** Emission Decay Data of Complexes 1–7

Compound	Medium	τ_1_ (ns)	τ_2_ (ns)	Compound	Medium	τ_1_ (ns)	τ_2_ (ns)
Complex **1**	MeCN	165	-	Complex **5**	MeCN	186	-
	MeOH	225	-				
	Buffer^a^	599	-		MeOH	243	-
	Cell culture medium^b^	355 (6.6%)	672 (94.6%)				
Complex **2**	MeCN	171	171	Complex **6**	MeCN	183	-
	MeOH	222	-				
	Buffer^a^	620	-		MeOH	248	-
	Cell culture medium^b^	584 (50.9%)	1167 (49.1%)				
Complex **3**	MeCN	173	-	Complex **7**	MeCN	178	-
	MeOH	229	-				
	Buffer^a^	340 (4.4%)	700 (95.6%)		MeOH	248	-
	Cell culture medium^b^	586 (43.3%)	1148 (56.7%)				
Complex **4**	MeCN	175	-	[Ru(bpy)_3_]Cl_2_	MeCN	156	-
	MeOH	230	-		MeOH	205	-
	Buffer^a^	266 (6.9%)	682 (93.1%)		Buffer^a^	329	-
	Cell culture medium^b^	588 (19.1%)	1279 (80.9%)		Cell culture medium^b^	357	-

a Tris-HCl buffer (0.05 M, pH = 7.4).

b DMEM containing 10% FBS (without phenol red).

As expected, based on their self-assembly properties, complexes **1**–**4** exhibited a significant enhancement in emission lifetimes and quantum yields in aqueous Tris-HCl buffer when compared to MeCN (Tables 1, 2 and Figure S50–S52). For example, the emission lifetime of complex **4** in Tris-HCl buffer (682 ns) increased by a factor of nearly four in comparison to that measured in MeCN solution (175 ns). Measuring [Ru­(bpy)_3_]­Cl_2_ as a control revealed only a 1.5-fold increase in its emission lifetime when the solvent was switched from MeCN (156 ns) to Tris-HCl buffer (329 ns). An increase of this magnitude is expected for complexes with the [Ru­(bpy)_3_]^2+^ core, as MeCN has a significantly larger concentration of dissolved oxygen, which is known to be the primary quencher of the ^3^MLCT excited state in these systems.^72^ Additionally, due to the polar nature of the ^3^MLCT excited state, an increase in the emission lifetime for complexes based on the [Ru­(bpy)_3_]^2+^ motif is expected in polar solvents and can be attributed to a slight stabilization of the ^3^MLCT excited state, thereby decreasing the efficiency of a thermal deactivation pathway involving a transition from the ^3^MLCT state to a low-lying metal-centered (MC) excited state.^73^ To further explore this hypothesis, we measured the emission lifetimes of complexes **1**–**7** and [Ru­(bpy)_3_]­Cl_2_ in MeOH, noting a slight increase in the lifetimes consistent with the stabilization of the ^3^MLCT excited state in polar environments.

However, our Ru­(II) complexes demonstrate substantially longer emission lifetimes in aqueous solutions, ranging from 672 to 1279 ns (Table 2), suggesting that factors beyond those discussed above are at play. We thus hypothesize that this enhancement stems from the formation of aggregates, as we observed in our DLS studies in aqueous environments (*vide supra*). Due to the aggregation, the metal headgroups are brought into closer proximity, thereby rigidifying the [Ru­(bpy)_3_]^2+^ chromophores, leading to suppression of the vibrational nonradiative decay pathways. Additionally, the formation of aggregates renders the chromophores less accessible to molecular oxygen. This explanation is in agreement with the increase in the quantum yield that was observed when the complexes were dissolved in Tris-HCl buffer in comparison to MeCN.^59^ According to the literature, self-assembled Ru­(II) complexes typically display biexponential emission decays, with a minor short-lived component corresponding to the nonaggregated species and a major long-lived component associated with the aggregated form.^59,74^ In our study, fitting the emission decays of complexes **1** and **2** in Tris-HCl buffer to a biexponential model revealed a short-lived component contributing less than 1% to the overall decay. Hence, we report only the monoexponential fitting results. In contrast, the decay profiles of complexes **3** and **4** exhibited more substantial contributions from the nonaggregated species, accounting for 4.4% and 6.9% of the overall decay, respectively (Table 2). Indicatively, complex **3** displays a single emission lifetime of 677 ns when we employ a monoexponential fit, though a biexponential fit reveals a minor component with a decay time of 340 ns (4.4%), consistent with a monomeric [Ru­(bpy)_3_]^2+^ chromophore, and a major component at 700 ns (95.6%), attributed to the aggregated species. This aggregation-induced enhancement is consistent with previous observations by De Cola and coworkers regarding Ru­(II) aggregate formation in aqueous media.^59^ To break the aggregates, we gradually added 100 μL aliquots of MeCN to a 20 μM solution of complex **3** in Tris-HCl, and the decay profiles remained largely unchanged until reaching 30% v/v MeCN and a final concentration of 13 μM, where we observed an abrupt disappearance of the long-lived component and a monoexponential decay with a time constant of 378 ns, signaling the breakage of the aggregates and a return to monomeric behavior (Figure S52K).^58^ Control experiments at 13 μM in pure Tris-HCl buffer confirmed aggregation, with a long-lived emission lifetime of 647 ns (Figure S52L). Additional testing of complex **3** in 5% DMSO/Tris-HCl buffer at 20 μM yielded a predominantly long-lived decay with an emission lifetime of 645 ns, again indicative of aggregation. To assess whether concentration influences the observed emission lifetime, we studied solutions of the complexes in both MeCN and Tris-HCl buffer over a concentration range of 10^–5^ to 10^–4^ μM (Figure S53a). No significant concentration-dependent variation in the measured emission lifetimes was observed, indicating that under the tested conditions, the solvent, rather than concentration, plays an important role in the aggregation behavior of the complexes.

Given the changes in the aggregation profiles of the complexes in cell culture medium containing FBS, as observed by DLS analysis, we also examined their emission lifetimes under these conditions (Table 2 and Figure S52F–J). For this analysis, an 80 μM solution of each complex was utilized to achieve an improved signal-to-background ratio. As shown in Table 2, [Ru­(bpy)_3_]^2+^ displays a monoexponential emission decay profile with a lifetime of 357 ns, which is consistent with its lifetime in Tris-HCl buffer. However, the emission decay behavior of complexes **2**–**4** differs markedly from that of the estrogen-free control and from that of [Ru­(bpy)_3_]^2+^. In a cell culture medium with 10% FBS, the decay profile of complex **1** can be fitted to a biexponential model, comprising a short-lived component (355 ns, 6.6% contribution), consistent with the monomeric species, and a long-lived component (672 ns, 93.4% contribution) attributable to the aggregated form. By contrast, estrogen-functionalized complexes **2**–**4** display biexponential decay profiles characterized by two long-lived components (ranging from 584 to 1279, Table 2) with comparable contributions and no detectable monomeric component. These findings are consistent with the DLS measurements in FBS-containing medium, where complexes **2**–**4** displayed more pronounced alterations in the size distribution profiles compared to complex **1**. Consistent with the behavior observed in Tris-HCl buffer, the addition of MeCN to a solution of complex **3** in FBS-containing medium led to aggregate disruption once the MeCN content reached 30%, as evidenced by a decrease in emission lifetime to 436 ns, signaling the dominance of the nonaggregated form (Figure 1f). Overall, our findings collectively confirm that aggregation correlates with the observed emission enhancement in aqueous solutions and that complexes **1**–**4** predominantly exist as self-assembled micelle-like aggregates in the cell culture medium used for our cellular studies. Nevertheless, it cannot be unequivocally determined from these experiments whether the complexes preserve their aggregated form in the intracellular environment.

### ROS Generation Studies

The triplet excited state of Ru­(II) polypyridyl complexes is highly sensitive to the presence of molecular oxygen, making them efficient type II photosensitizers that produce ^1^O_2_ with high quantum yields.^9^ To assess ROS generation, we used two different assays, namely 9,10-anthracenediyl-bis­(methylene)­dimalonic acid (ABDA) for assessing type II ROS production and dihydrorhodamine-123 (DHR-123) for type I ROS generation.^75^ ABDA reacts selectively with ^1^O_2_ leading to a decrease in the absorbance of the dye. On the other hand, DHR-123 is oxidized by various type I ROS species, including superoxide and peroxide anions, to yield the highly fluorescent rhodamine-123. Based on the results of the ABDA assay, which are summarized in Figure 2a, all complexes efficiently generate ^1^O_2_, and as expected, complexes **5**–**7** exhibit stronger ^1^O_2_ production which can be attributed to their increased molar absorptivity due to the presence of the DIP ligand.^76,77^ In general, [Ru­(bpy)_3_]^2+^-type photosensitizers are known to exhibit high photostability under irradiation.^70,78^ In our case, this was also confirmed by monitoring the UV–vis absorption spectra during the ABDA assay. As demonstrated in Figure S55, irradiation at the MLCT absorption band (457 nm) did not alter the characteristic ^1^MLCT absorption features of the complexes (400–500 nm), thereby validating their photostability under the experimental conditions employed. Based on the DHR-123 assay, all complexes were proven to efficiently produce ROS at comparable levels and showed scores similar to those of [Ru­(bpy)_3_]^2+^ (Figure 2b). In summary, while all complexes can generate both type I and type II ROS, evidence indicates that complexes **5**–**7** exhibit greater potential as type II photosensitizers.

**Figure 2:**
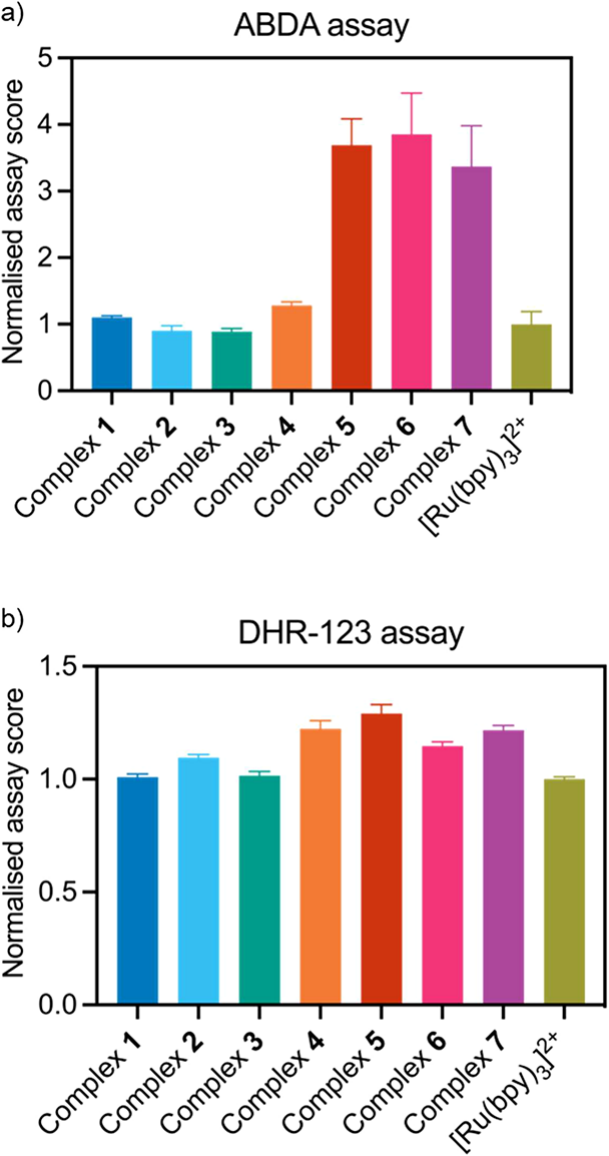
ROS generation studies of the synthesized complexes: (a) ABDA assay: ABDA (100 μM) and the respective complex (10 μM) were combined in PBS and irradiated using a 457 nm LED. (b) DHR-123 assay: DHR-123 (10 μM) and the respective complex (1 μΜ) were combined in PBS and irradiated using a 457 nm LED. The DHR-123 dye was monitored using λ_ex_ = 505 nm and λ_em_ = 535 nm. The assay scores were calculated by plotting the ABDA absorbance or the DHR-123 emission over time and then fitting to a linear regression (Figures S56 and S57). The gradients were then normalized to that of [Ru­(bpy)_3_]^2+^.

### Cell Studies

We determined the cytotoxicity of all the complexes using the well-established MTS assay^79^ against two breast cancer cell lines: MCF-7, which is ER+, and MDA-MB-453, which is ER-. [Ru­(bpy)_3_]^2+^ was used as a control.

Initially, the cytotoxicity of the complexes was assessed in the dark over a concentration range of 30 nM to 100 μM for complexes **1**–**4** and 30 nM to 20 μM for **5**–**7** (due to complex absorbance interference with the MTS assay at concentrations >20 μM), and none of them was found to induce a significant cytotoxic effect (Figures S58 and S60). Therefore, all complexes proceeded to the next stage of the light-induced cytotoxicity evaluation. In this experiment, the cells were irradiated with blue light (457 nm) for 12 min.

As illustrated in Table 3, complexes **3**–**7** exhibit notable light-dependent toxicity under the tested conditions, with a more pronounced effect on ER+ MCF-7 cells compared to ER-MDA-MB-453 cells for complexes **3** and **4**. In contrast, [Ru­(bpy)_3_]^2+^ and complex **1** display no light-induced phototoxicity within the tested concentration range. Interestingly, complex **2**, which contains ethinyl estradiol, is significantly less phototoxic than its mestranol and quinestrol analogues (complexes **3** and **4**). This reduced phototoxicity observed for complexes **1** and **2** and [Ru­(bpy)_3_]^2+^ can be attributed to their hydrophilic nature, which limits their cellular uptake.^80^ In contrast, the less optimal behavior of complexes **5**–**7**, as reflected by their lower SI values (Table 3), may be attributed to their higher lipophilicity, resulting in reduced solubility and potential precipitation under the experimental conditions. Complexes **3** and **4** display significant phototoxicity, with complex **4** exhibiting a phototherapeutic index (PI, defined as [IC_50_]­dark/[IC_50_]­light) of >52.36 in ER+ cells. When comparing the [IC_50_]­light values with the concentration ranges used to study the aggregation behavior of the complexes, we observe that they fall within the same order of magnitude, namely, the micromolar range. In particular, complex **3**, which exhibits a higher [IC_50_]­light value of 90.96 μM, exists in its aggregated form at these concentrations, as confirmed by DLS studies (*vide supra*). Overall, the observed [IC_50_]­light values, together with the DLS and emission lifetime data, provide strong evidence that the complexes exist predominantly in their aggregated forms under the conditions utilized for our cellular studies.

**Table 3:** Cytotoxicity Data of Complexes 1–7^c^

Compound	[IC_50(ER+)_]light	[IC_50(ER‑)_]light	SI^a^
[Ru(bpy)_3_]Cl_2_	>100	>100	NPD^b^
Complex **1**	>100	>100	NPD^b^
Complex **2**	>100	85.72	>0.86
Complex **3**	9.47	90.96	9.61
Complex **4**	1.91	6.27	3.28
Complex **5**	7.29	4.72	0.65
Complex **6**	4.25	2.53	0.60
Complex **7**	3.32	1.97	0.59

a SI = selectivity index, defined as [IC_50(ER‑)_]­light/[IC_50(ER+)_]­light.

b NPD = no phototoxicity detected under the tested concentration range.

c IC_50_ values are given in μM.

The highest selectivity was observed for complex **3**, where the reduction in the ER+ cell population was found to be approximately ten times greater than that in ER– cells. The increased internalization of complex **3** in ER+ cells, in comparison to that in ER– cells, is also supported by fluorescence microscopy. As shown in Figure 3a, ER+ cells exhibit intense nonnuclear red luminescence, whereas ER– cells exhibit only very weak luminescence under similar experimental conditions. Similarly, complex **4** displays approximately a 3-fold selectivity between ER+ and ER– cell lines, a lower contrast than the 10-fold selectivity observed for complex **3**. Microscopy images of ER+ and ER– cells treated with complexes **1**, **3**, and **4** further highlight these differences (Figure S62). Complex **1**, which is estrogen-free, shows no notable difference in luminescence between the two cell lines, indicating nonselective internalization. In contrast, complexes **3** and **4** both display significantly higher intracellular luminescence in ER+ cells, with complex **4** exhibiting the greatest overall luminescence. These results further support the preferential uptake of these complexes in ER+ cells, reinforcing the hypothesis that estrogen conjugation promotes selective internalization. Notably, the observed selectivity indices are comparable to—or in some cases exceed—those reported for other estrogen-modified photosensitizers in the literature.^81,82^

**Figure 3:**
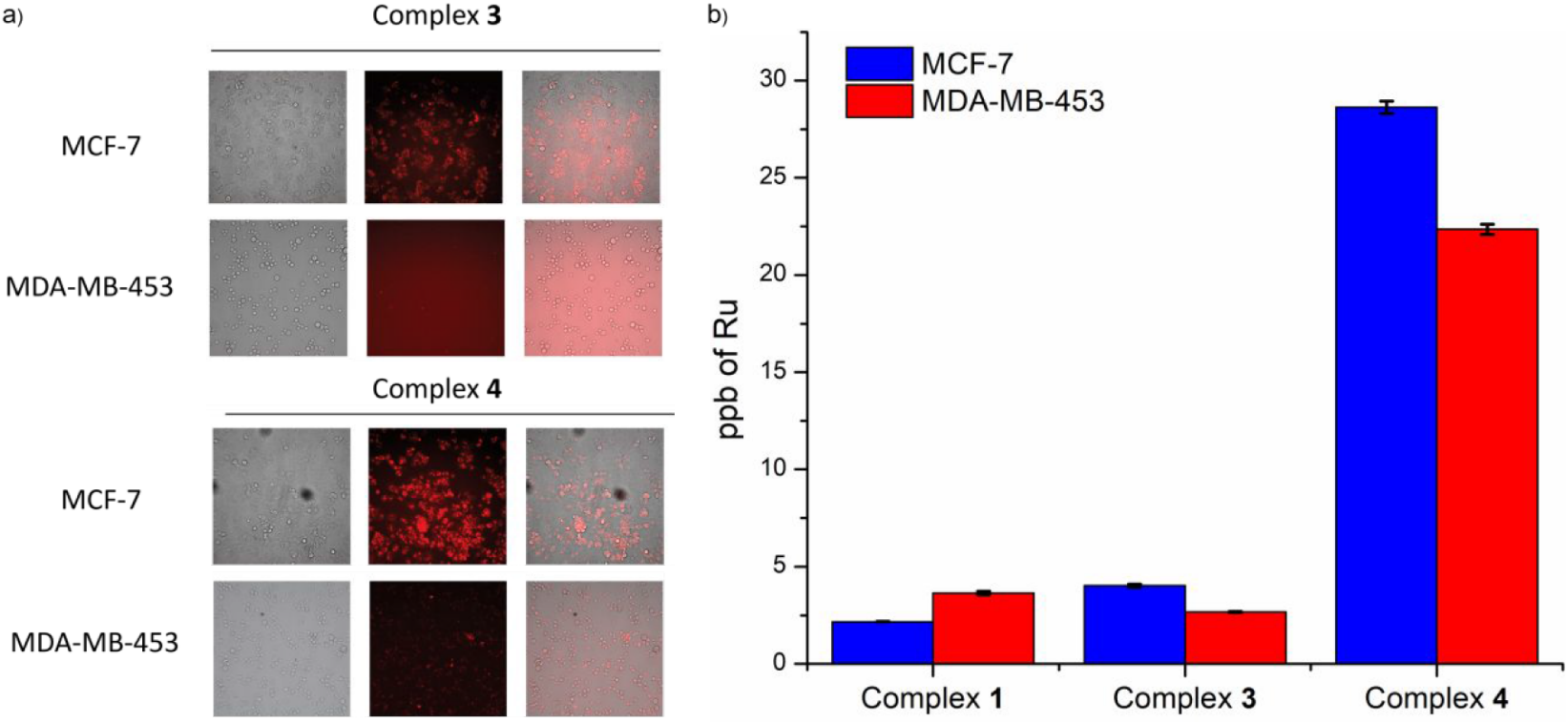
Microscopy imaging of cells incubated with complex **3** (20 μΜ) and complex **4** (10 μΜ) for 6 h: brightfield (left), photosensitizer luminescence (middle), and overlaid (right) images. (b) Cellular uptake by ICP-MS of complexes **1**, **3**, and **4** (20 μM, 6 h) in MCF-7 (ER+) and MDA-MB-453 (ER−) cells.

Given that complexes **3** and **4** demonstrated the most promising phototoxic profiles, it was of interest to quantify the amount of ruthenium taken up by the treated cells. Thus, the cellular uptake of complexes **3** and **4** was evaluated and compared to the control complex **1** in ER+ (MCF-7) and ER– (MDA-MB-453) cell lines using inductively coupled plasma mass spectrometry (ICP-MS) (Figure 3b). Control complex **1** exhibited slightly higher accumulation in ER– cells than in ER+ cells, as determined by ICP-MS, underscoring its lack of ER-targeting. In contrast, complex **3** demonstrated 1.5-fold greater uptake in ER+ cells compared to ER– cells, corroborating both imaging and cytotoxicity data, which indicated enhanced intracellular luminescence (Figures S62 and 3a) and increased phototoxicity in ER+ cells. Complex **4** exhibited higher overall cellular uptake than both complexes **1** and **3** in both cell lines while showing greater uptake in ER+ versus ER– cells by a factor of 1.3, as determined by ICP-MS. This trend is consistent with its increased lipophilicity and the brighter red luminescence observed by microscopy for this complex. It is well established that lipophilicity can enhance membrane permeability and cellular internalization.^83^ It is worth mentioning that, complex **4** showed a slightly reduced accumulation in ER+ cells versus ER– cells by ICP-MS (1.3-fold) compared to complex **3** (1.5-fold), mirroring the trend observed in phototoxicity assays and suggesting a trade-off between total uptake and selectivity. Overall, the ICP-MS results support the imaging and phototoxicity data, reinforcing that complexes **3** and **4** exhibit preferential accumulation in ER+ cells, with complex **3** displaying the highest selectivity across all tested parameters. To the best of our knowledge, this work is the first to quantitatively demonstrate the use of estrogen-functionalized Ru­(II) polypyridyl complexes for targeting ER+ cancer cells.^25,34,41^

While these findings are highly promising, further studies are required to fully elucidate the cellular uptake mechanisms and specific modes of action of these complexes. The observed estrogen-dependent phototoxicity and preferential accumulation of complexes **3** and **4** in ER+ cells may not be directly mediated by estrogen receptors, particularly considering the limited accessibility of the estrogen moiety within the self-assembled aggregates. Nevertheless, despite this potential shielding effect in the micelle-like structures, our results from three independent methods—cytotoxicity assays, fluorescence microscopy, and ICP-MS—demonstrate that estrogen functionalization enhances selectivity toward ER+ cells. This is particularly evident when comparing **3** and **4** with their estrogen-free analogue, complex **1**. ICP-MS measurements reveal that complex **1** accumulates slightly more in ER– cells than in ER+ cells, whereas complexes **3** and **4** display the opposite trend, with higher accumulation in ER+ cells. These findings underscore the role of the estrogen moiety in promoting preferential internalization, even when its availability may be partially restricted by aggregation. Given that complexes **3** and **4** form micelle-like self-assemblies of suitable size for cellular uptake in FBS-containing medium, their entry into cancer cells may be facilitated by aggregation, possibly through mechanisms such as endocytosis.^65^

## Conclusions

In this study, we successfully synthesized and characterized a series of estrogen-functionalized Ru­(II) polypyridyl complexes designed to selectively target ER+ cancer cells for PDT. The incorporation of estrogen derivatives into the Ru­(II) complexes was achieved via CuAAC reactions for the synthesis of the ligands, followed by complexation with Ru­(II) centers. Partition coefficient studies revealed that the estrogen-functionalized complexes display a range of lipophilicities depending on both the ancillary ligands and the specific estrogen derivative. In addition, based on our DLS studies, the complexes display aggregation in aqueous media, suggesting that self-assembly into micelle-like structures is driven by the amphiphilic nature of the Ru­(II) complexes, as well as interaction with serum proteins when FBS-containing cell culture medium is utilized. This aggregation behavior is hypothesized to contribute to the enhanced emission lifetimes we reported in Tris-HCl buffer and is also consistent with the increased emission lifetimes observed in the FBS-containing cell culture medium. Subsequent assays (ABDA and DHR-123) confirmed that all of the complexes efficiently generate ROS upon irradiation with blue light. Phototoxicity studies revealed that complexes **3** and **4** exhibit a degree of cell-type-dependent cytotoxicity, with the mestranol-functionalized complex **3** demonstrating a remarkable 9.6-fold phototoxic effect in ER+ cells compared with ER- cells. Cellular uptake studies by ICP-MS are consistent with the phototoxicity trends, with complex **3** displaying a 1.5-fold higher accumulation in ER+ cells compared to ER- cells. ICP-MS results for complex **4** showed greater total uptake but reduced selectivity in comparison to complex **3**, which is in line with the phototoxicity and imaging results. Overall, the data are consistent across three orthogonal methods, all of which show that the estrogen-containing derivatives **3** and **4** display a degree of selectivity for ER+ cell lines.

The observed [IC_50_]­light values, in combination with DLS and emission lifetime data, strongly indicate that the complexes exist primarily in their aggregated form in the cell culture medium. Although the availability of the estrogen moiety may be partially restricted within aggregates, cytotoxicity assays, luminescence microscopy, and ICP-MS consistently show that complexes **3** and **4** preferentially accumulate in ER+ cells, unlike the estrogen-free analogue. These results highlight the potential of estrogen functionalization as a strategy to improve PDT selectivity. Furthermore, our results suggest that the formation of aggregates of appropriate size in biologically relevant media may contribute to the preferential uptake of complexes **3** and **4**. To the best of our knowledge, this study represents the first demonstration of ER+ cancer cell targeting for PDT using estrogen-functionalized Ru­(II) polypyridyl complexes.

In conclusion, this study highlights the potential of estrogen-decorated Ru­(II) polypyridyl complexes as photosensitizers and establishes a strong foundation for further optimization. The results underscore the importance of ligand design in enhancing selective uptake and phototoxicity, as well as advancing the development of targeted PDT agents. While these results are promising, further investigations are necessary to elucidate the exact mechanisms of internalization and action of these complexes. Future work could include receptor-blocking experiments or low-temperature cellular uptake evaluations to determine whether internalization is receptor-mediated.

## Experimental Section

### Materials

All commercially available starting materials and solvents were purchased from commercial suppliers (Sigma-Aldrich, TCI Chemicals, Thermo Fisher Scientific, Fluorochem, Honeywell, Chem-Lab) and were used without further purification. 5-Azido-1,10-phenanthroline^84,85^ and Ru­(DIP)_2_Cl_2_ ^86,87^ were prepared as previously described, and spectroscopic data were in accordance with the literature.

### Instrumentation and Methods

^1^H, ^1^H–^1^H COSY, and ^13^C NMR spectra were recorded on a 500 MHz NMR spectrometer (Agilent). Absorption spectra were acquired by using a JASCO V-750 spectrophotometer. For recording the emission spectra, we used an Edinburgh Instruments FS5 spectrofluorometer equipped with a red-sensitive Hamamatsu R13456 photomultiplier tube (PMT) and a 150 W xenon arc lamp as the light source. All spectra were corrected for detector response using the correction files and software (Fluoracle) provided by the manufacturer. The luminescence quantum yields of the complexes were calculated using the optically dilute method^88^ with an air-equilibrated aqueous solution of [Ru­(bpy)_3_]­Cl_2_ (Φ_em_ = 0.049, λ_ex_ = 436 nm) as the standard solution.^55,89^ Emission lifetime measurements were performed using an Edinburgh Instruments mini-τ lifetime spectrometer with a bandpass filter (±40 nm bandpass) at 650 nm. The excitation source was an Edinburgh Instruments picosecond pulsed LED (EPLED-320) with a peak wavelength of 326.8 nm and a pulse width of 910 ps. The detector was a thermoelectrically cooled, high-speed, red-sensitive photomultiplier tube (Hamamatsu H10720-01). The data were analyzed by using the software provided by the manufacturer (Fluoracle). For emission lifetime calculations, the experiments were performed in triplicate, yielding similar results. The MS spectra were acquired using an LC20AD Shimadzu connected to Shimadzu LCMS-2010EV, with a flow rate of 0.4 mL/min MeOH or MeCN + 0.1% formic acid. LCMS analysis was carried out on an Agilent 1260 Infinity with a Raptor C18 column (50 mm × 2.1 mm, 2.7 μm particle size). A two-minute gradient from 5% to 95% MeCN in water was used, supplemented with 0.1% formic acid.

#### Synthesis

##### General Procedure A

Ligand synthesis via click chemistry. 5-Azido-1,10-phenanthroline was dissolved in DCM (30 mL) under an argon atmosphere, and each terminal alkyne was added. A solution of CuSO_4_·5H_2_O (132 mg, 0.5 mmol, 2.5 equiv) in water (5 mL) was prepared and added to the reaction mixture. Then, a solution of sodium ascorbate (210 mg, 1 mmol, 5 equiv) in water (5 mL) was prepared and added to the reaction mixture as well. The reaction mixture was degassed for 10 min and then stirred for 4 h at room temperature. The organic layer was separated and washed with an EDTA solution (0.01 M) until the EDTA solution no longer turned blue. Next, the organic phase was washed three times with brine, dried over Mg_2_SO_4_, filtered, and concentrated *in vacuo*. The crude product was purified by flash chromatography using EtOAc/iPrOH/NH_3_ (5:0.5:0.25) as a solvent system. The combined product-containing fractions were concentrated and dried.

##### General Procedure B

Synthesis of the [Ru­(bpy)_2_(L-1)]­Cl_2_ type. A mixture of *cis*-[Ru­(bpy)_2_Cl_2_] and each **L-1** ligand in 4 mL of EtOH/H_2_O (1:1) was refluxed overnight under an argon atmosphere. The color of the solution changed from purple to deep red. The reaction mixture was allowed to cool to room temperature, and the solvent was evaporated. The crude product was purified by flash chromatography using MeCN/KNO_3(aq)_ (8:2 (0.05 M)) as the solvent system. The combined product-containing fractions were concentrated and redissolved in water with a few drops of acetone. A saturated aqueous solution of NH_4_PF_6_ was added to precipitate a red solid. The solid was collected by filtration, washed with water, and dried. Half of this PF_6_ salt was converted to the chloride salt using Amberlite IRA 410.

##### General Procedure C

Synthesis of the [Ru­(DIP)_2_(**L-1**)]­Cl_2_ type. A mixture of *cis*-[Ru­(DIP)_2_Cl_2_] and each **L-1** ligand in 4 mL of EtOH/H_2_O (1:1) was refluxed overnight under an argon atmosphere. The color of the solution changed from purple to deep red. The reaction mixture was allowed to cool to room temperature, and the solvent was evaporated. The crude product was purified by flash chromatography using MeCN/KNO_3(aq)_ (10:1 (0.05 M)) as the solvent system. The combined product-containing fractions were concentrated and redissolved in an EtOH/H_2_O mixture. A saturated aqueous solution of NH_4_PF_6_ was added to precipitate a red solid. The mixture was concentrated until all ethanol was removed, and the solid was collected by filtration, washed with water, and dried. Half of this PF_6_ salt was converted to the chloride salt using Amberlite IRA 410.

5-(4-Butyl-1*H*-1,2,3-triazol-1-yl)-1,10-phenanthroline (**L-1a**). According to General Procedure A, 5-azido-1,10-phenanthroline (44 mg, 0.2 mmol) and 1-hexyne (22 μL, 0.19 mmol) were utilized. **L-1a** was obtained as a pale yellow solid (35 mg, 0.12 mmol, 58%). ^1^H NMR (500 MHz, CDCl_3_) δ 9.32–9.23 (m, 2H), 8.32 (dd, *J* = 8.1, 1.5 Hz, 1H), 8.15 (dd, *J* = 8.4, 1.4 Hz, 1H), 7.94 (s, 1H), 7.76 (s, 1H), 7.73 (dd, *J* = 8.1, 4.3 Hz, 1H), 7.68 (dd, *J* = 8.4, 4.3 Hz, 1H), 2.93–2.87 (m, 2H), 1.84–1.77 (m, 2H), 1.53–1.45 (m, 2H), 1.00 (t, *J* = 7.4 Hz, 3H). ^13^C NMR (126 MHz, CDCl_3_) δ 151.9, 151.5, 149.1, 146.6, 146.3, 136.7, 132.4, 132.0, 127.2, 124.8, 124.1, 123.9, 123.5, 123.4, 31.6, 25.6, 22.3, 13.7. ESI-MS calculated for *m*/*z* [M + H]^+^ 304.16; found: 304.00.

Ethinyl estradiol-functionalized phenanthroline (**L-1b**). 5-Azido-1,10-phenanthroline (44 mg, 0.2 mmol) and ethinyl estradiol (56 mg, 0.19 mmol) were dissolved in a mixture of t-butanol/H_2_O (1:1) under an argon atmosphere. CuSO_4_·5H_2_O (132 mg, 0.5 mmol, 2.5 equiv) and sodium ascorbate (210 mg, 1 mmol, 5 equiv) were added to the reaction mixture. The reaction mixture was degassed for 10 min and then refluxed overnight. The next day, the reaction mixture was extracted using ethyl acetate (80 mL) and an EDTA solution (0.01 M) until the EDTA solution did not turn blue. Next, the organic phase was washed three times with brine, dried over MgSO_4_, filtered, and concentrated *in vacuo*. The crude product was obtained as a pale yellow solid and was used without any further purification (77 mg, 0.15 mmol, 74%). ^1^H NMR (500 MHz, CD_3_OD) δ 9.20–9.16 (m, 2H), 8.56 (dd, *J* = 8.2, 1.5 Hz, 1H), 8.35 (s, 1H), 8.22 (d, *J* = 7.3 Hz, 1H), 8.17 (dd, *J* = 8.3, 1.4 Hz, 1H), 7.83 (ddd, *J* = 16.0, 8.0, 4.1 Hz, 2H), 7.01 (d, *J* = 8.5 Hz, 1H), 6.45 (dd, *J* = 7.4, 2.5 Hz, 2H), 2.74 (d, *J* = 3.6 Hz, 2H), 2.63–2.59 (m, 1H), 2.25–2.21 (m, 2H), 2.09–2.05 (m, 1H), 1.93 (d, *J* = 4.7 Hz, 1H), 1.84 (t, *J* = 9.1 Hz, 1H), 1.78–1.72 (m, 3H), 1.47–1.41 (m, 2H), 1.32–1.26 (m, 2H). ^13^C NMR (126 MHz, DMSO) δ 154.9, 137.2, 137.2, 137.1, 130.4, 130.3, 126.1, 126.0, 126.0, 125.5, 124.2, 124.2, 114.9, 112.7, 109.6, 105.8, 102.5, 89.0, 81.3, 78.2, 49.0, 47.9, 47.0, 46.7, 43.3, 32.9, 32.6, 31.3, 29.1, 26.1, 14.5, 12.8. ESI-MS calculated for *m*/*z* [M + H]^+^ 518.26; found: 518.20.

Mestranol-functionalized phenanthroline (**L-1c**). According to General Procedure A, 5-azido-1,10-phenanthroline (44 mg, 0.2 mmol) and mestranol (59 mg, 0.19 mmol) were used. **L-1c** was obtained as a pale yellow solid (87 mg, 0.16 mmol, 82%). ^1^H NMR (500 MHz, CDCl_3_) δ 9.30 (ddd, *J* = 6.3, 4.3, 1.6 Hz, 2H), 8.35 (dd, *J* = 8.1, 1.6 Hz, 1H), 8.18 (dd, *J* = 8.4, 1.6 Hz, 1H), 8.01 (s, 1H), 7.95 (s, 1H), 7.75 (dd, *J* = 8.0, 4.3 Hz, 1H), 7.71 (dd, *J* = 8.4, 4.3 Hz, 1H), 7.16 (d, *J* = 8.6 Hz, 1H), 6.69 (dd, *J* = 8.6, 2.7 Hz, 1H), 6.63 (d, *J* = 2.6 Hz, 1H), 3.77 (s, 3H), 2.86 (dd, *J* = 12.7, 7.9 Hz, 2H), 2.64–2.58 (m, 1H), 2.28 – 2.21 (m, 2H), 2.06 (d, *J* = 12.2 Hz, 1H), 1.99–1.95 (m, 1H), 1.81–1.75 (m, 2H), 1.59–1.46 (m, 4H), 1.41 (dd, *J* = 12.2, 6.4 Hz, 1H), 1.25 (s, 1H), 1.12 (s, 3H). ^13^C NMR (126 MHz, CDCl_3_) δ: 157.4, 154.2, 151.7, 151.3, 146.4, 146.1, 137.9, 136.5, 132.3, 132.0, 131.7, 126.9, 126.1, 124.5, 123.9, 123.9, 123.8, 123.4, 113.7, 111.4, 82.6, 55.1, 48.6, 47.5, 43.4, 39.5, 38.2, 33.1, 29.8, 27.3, 26.2, 23.5, 14.2. ESI-MS calculated for *m*/*z* [M + Na]^+^ 554.25; found: 554.00

Quinestrol-functionalized phenanthroline (**L-1d**). According to General Procedure A, 5-azido-1,10-phenanthroline (44 mg, 0.2 mmol) and quinestrol (69 mg, 0.19 mmol) were used. **L-1d** was obtained as a pale yellow solid (94 mg, 0.16 mmol, 82%). ^1^H NMR (500 MHz, CDCl_3_) δ 9.31–9.27 (m, 2H), 8.34 (d, *J* = 7.7 Hz, 1H), 8.17 (d, *J* = 8.3 Hz, 1H), 8.00 (s, 1H), 7.96 (s, 1H), 7.74 (dd, *J* = 8.0, 4.3 Hz, 1H), 7.71 (dd, *J* = 8.4, 4.3 Hz, 1H), 7.12 (d, *J* = 8.6 Hz, 1H), 6.66–6.63 (m, 1H), 6.59 (s, 1H), 4.70 (dd, *J* = 5.4, 3.0 Hz, 1H), 2.84 (dt, *J* = 16.6, 8.2 Hz, 2H), 2.62–2.56 (m, 1H), 2.24 (dd, *J* = 16.6, 8.1 Hz, 2H), 1.87–1.75 (m, 9H), 1.57 (ddd, *J* = 29.8, 15.5, 4.9 Hz, 5H), 1.44–1.36 (m, 2H), 1.12 (s, 3H). ^13^C NMR (126 MHz, CDCl_3_) δ: 156.2, 154.5, 152.1, 151.6, 146.7, 146.4, 138.1, 136.9, 132.3, 132.2, 132.1, 127.2, 126.4, 124.8, 124.3, 124.2, 124.2, 123.8, 115.8, 113.1, 82.9, 79.2, 49.0, 47.8, 43.8, 39.8, 38.5, 33.5, 33.1, 30.1, 30.1, 27.7, 26.5, 24.2, 23.8, 22.9, 14.6. ESI-MS calculated for *m*/*z* [M + H]^+^ 586.32; found: 586.20.

[Ru­(bpy)_2_(**L-1a**)]­Cl_2_, (**1**). According to General Procedure B, *cis*-[Ru­(bpy)_2_Cl_2_] (44 mg, 0.09 mmol) and **L-1a** (31 mg, 0.1 mmol) were utilized. [**1**]­(PF_6_)_2_ was obtained as a red solid (80 mg, 0.08 mmol, 90%). ^1^H NMR (500 MHz, CD_3_CN) δ 8.75 (d, *J* = 8.1 Hz, 1H), 8.72 (d, *J* = 8.2 Hz, 2H), 8.68 (d, *J* = 8.2 Hz, 2H), 8.56 (s, 1H), 8.52 (d, *J* = 8.3 Hz, 1H), 8.25 (s, 1H), 8.20 (d, *J* = 5.1 Hz, 2H), 8.13 (t, *J* = 7.9 Hz, 2H), 8.03 (t, *J* = 7.8 Hz, 2H), 7.86 (d, *J* = 5.4 Hz, 2H), 7.82 (dd, *J* = 8.2, 5.3 Hz, 1H), 7.77 (dd, *J* = 8.6, 5.2 Hz, 1H), 7.61 (d, *J* = 5.6 Hz, 2H), 7.47 (t, *J* = 6.4 Hz, 2H), 7.27 (t, *J* = 6.6 Hz, 2H), 2.87 (t, *J* = 7.7 Hz, 2H), 1.83–1.73 (m, 2H), 1.49 (dt, *J* = 14.9, 7.4 Hz, 2H), 0.99 (t, *J* = 7.4 Hz, 2H).^13^C NMR (126 MHz, CD_3_CN) δ 158.2, 158.2, 157.9, 157.9, 157.8, 155.6, 154.9, 154.6, 154.4, 153.0, 152.9, 152.2, 150.5, 149.7, 149.1, 148.4, 138.9, 138.8, 138.2, 134.6, 134.3, 130.6, 128.5, 128.4, 128.1, 127.7, 127.7, 127.5, 125.5, 125.5, 125.4, 125.4, 125.2, 125.1, 32.0, 25.8, 23.0, 14.0. ES-HRMS calculated for *m*/*z* [M]^2+^ = 358.5956; found 358.5940.

[Ru­(bpy)_2_(**L-1b**)]­Cl_2_, (**2**). According to General Procedure B, *cis*-[Ru­(bpy)_2_Cl_2_] (44 mg, 0.09 mmol) and **L-1b** (52 mg, 0.1 mmol) were utilized. [**2**]­(PF_6_)_2_ was obtained as a red solid (84 mg, 0.07 mmol, 82%).^1^H NMR (500 MHz, CD_3_CN) δ 8.69 (d, *J* = 7.8 Hz, 1H), 8.58–8.49 (m, 6H), 8.26 (s, 1H), 8.20 (d, *J* = 5.2 Hz, 2H), 8.13 (dd, *J* = 11.2, 4.6 Hz, 2H), 8.03 (t, *J* = 7.9 Hz, 2H), 7.87 (d, *J* = 5.5 Hz, 2H), 7.84–7.78 (m, 2H), 7.61 (d, *J* = 5.6 Hz, 2H), 7.48 (t, *J* = 6.0 Hz, 2H), 7.31–7.26 (m, 2H), 7.07 (d, *J* = 8.3 Hz, 1H), 6.62 (s, 1H), 6.56–6.51 (m, 2H), 3.60 (d, *J* = 12.6 Hz, 3H), 2.86–2.76 (m, 3H), 2.59–2.52 (m, 2H), 1.86–1.81 (m, 2H), 1.71–1.62 (m, 3H), 1.54 (dd, *J* = 22.1, 10.9 Hz, 2H), 1.45 (d, *J* = 11.4 Hz, 1H), 1.36 (dd, *J* = 12.0, 5.7 Hz, 2H). ^13^C NMR (126 MHz, CD_3_CN) δ 157.2, 157.2, 157.0, 154.5, 153.7, 153.5, 152.1, 152.0, 148.2, 147.6, 140.2, 139.2, 138.1, 138.0, 138.0, 137.9, 137.9, 137.2, 131.6, 129.6, 127.6, 127.6, 127.5, 127.5, 126.8, 126.8, 126.7, 126.3, 124.8, 124.4, 124.4, 124.3, 124.3, 124.3, 124.3, 124.3, 124.3, 124.2, 115.0, 112.5, 48.4, 47.4, 43.5, 43.5, 39.5, 37.6, 33.0, 29.3, 27.4, 26.3, 23.4, 13.9. ES-HRMS calculated for *m*/*z* [M]^2+^ = 465.6455; found 465.6437.

[Ru­(bpy)_2_(**L-1c**)]­Cl_2_, (**3**). According to General Procedure B, *cis*-[Ru­(bpy)_2_Cl_2_] (44 mg, 0.09 mmol) and **L-1c** (53 mg, 0.1 mmol) were used. [**3**]­(PF_6_)_2_ was obtained as a red solid (88 mg, 0.07 mmol, 87%). ^1^H NMR (500 MHz, CD_3_CN) δ 8.69 (d, *J* = 7.5 Hz, 1H), 8.53 (ddd, *J* = 12.0, 8.3, 4.9 Hz, 6H), 8.26 (s, 1H), 8.20 (d, *J* = 5.2 Hz, 2H), 8.15–8.11 (m, 2H), 8.03 (t, *J* = 7.9 Hz, 2H), 7.87 (d, *J* = 5.6 Hz, 2H), 7.81 (ddd, *J* = 15.9, 8.4, 5.2 Hz, 2H), 7.61 (d, *J* = 5.6 Hz, 2H), 7.50–7.46 (m, 2H), 7.30–7.25 (m, 2H), 7.16 (d, *J* = 7.9 Hz, 1H), 6.69–6.64 (m, 2H), 3.74 (s, 3H), 2.90–2.80 (m, 2H), 2.58–2.52 (m, 1H), 2.29–2.21 (m, 2H), 2.05–1.99 (m, 3H), 1.90–1.82 (m, 2H), 1.73–1.34 (m, 8H), 1.29 (s, 1H), 1.10 (s, 3H). ^13^C NMR (126 MHz, CD_3_CN) δ 158.1, 158.1, 157.8, 156.8, 156.1, 156.1, 154.6, 154.4, 154.4, 153.0, 153.0, 152.9, 151.1, 149.1, 148.4, 138.9, 138.8, 138.1, 134.5, 134.2, 133.1, 130.6, 128.5, 128.4, 128.3, 128.0, 127.7, 127.6, 127.1, 125.9, 125.9, 125.9, 125.3, 125.3, 125.2, 125.2, 125.2, 125.2, 116.3, 113.8, 83.0, 48.3, 44.4, 40.4, 33.9, 33.4, 30.4, 30.3, 28.3, 27.1, 24.6, 24.4, 14.8. ES-HRMS calculated for *m*/*z* [M]^2+^ = 472.6534; found 472.6510.

[Ru­(bpy)_2_(**L-1d**)]­Cl_2_, (**4**). According to General Procedure B, *cis*-[Ru­(bpy)_2_Cl_2_] (44 mg, 0.09 mmol) and **L-1d** (59 mg, 0.1 mmol) were used. [**4**]­(PF_6_)_2_ was obtained as a red solid (90 mg, 0.07 mmol, 83%). ^1^H NMR (500 MHz, CD_3_CN) δ 8.68 (d, *J* = 8.2 Hz, 1H), 8.55 (d, *J* = 8.2 Hz, 2H), 8.50 (d, *J* = 8.5 Hz, 3H), 8.48 (d, *J* = 1.5 Hz, 1H), 8.24 (s, 1H), 8.19 (d, *J* = 4.8 Hz, 2H), 8.11 (tdd, *J* = 8.1, 3.2, 1.3 Hz, 2H), 8.01 (dd, *J* = 11.5, 4.4 Hz, 2H), 7.85 (d, *J* = 5.6 Hz, 2H), 7.81 (dd, *J* = 8.2, 5.3 Hz, 1H), 7.79–7.75 (m, 1H), 7.60 (d, *J* = 5.4 Hz, 2H), 7.49–7.44 (m, 2H), 7.26 (t, *J* = 6.6 Hz, 2H), 7.10 (dd, *J* = 8.5, 2.0 Hz, 1H), 6.60 (d, *J* = 6.8 Hz, 1H), 6.57 (d, *J* = 2.4 Hz, 1H), 4.73 (dt, *J* = 8.3, 4.3 Hz, 1H), 2.82 (qd, *J* = 17.0, 8.4 Hz, 2H), 2.60 – 2.49 (m, 1H), 2.26–2.19 (m, 2H), 2.03–1.97 (m, 2H), 1.85 (ddd, *J* = 11.2, 10.4, 5.1 Hz, 3H), 1.73 – 1.67 (m, 4H), 1.61 (ddd, *J* = 8.5, 7.7, 4.0 Hz, 3H), 1.52 (d, *J* = 10.9 Hz, 1H), 1.45–1.30 (m, 3H), 1.07 (s, 3H). ^13^C NMR (126 MHz, CD_3_CN) δ 158.2, 158.1, 157.9, 156.8, 156.0, 156.0, 154.7, 154.4, 153.0, 153.0, 152.9, 149.1, 148.5, 138.9, 138.8, 138.1, 134.5, 134.2, 134.2, 134.2, 133.1, 130.6, 128.6, 128.5, 128.4, 128.1, 127.7, 127.6, 127.1, 125.8, 125.7, 125.3, 125.3, 125.3, 125.2, 125.2, 116.4, 113.8, 83.1, 79.8, 49.4, 48.3, 44.4, 44.4, 40.4, 38.5, 33.9, 33.4, 31.9, 30.4, 28.3, 27.2, 24.6, 24.3, 14.8. ES-HRMS calculated for *m*/*z* [M]^2+^ = 499.6769; found 499.6770.

[Ru­(DIP)_2_(**L-1b**)]­Cl_2_, (**5**). According to General Procedure C, *cis*-[Ru­(DIP)_2_Cl_2_] (75 mg, 0.09 mmol) and **L-1b** (52 mg, 0.1 mmol) were utilized. [**5**]­(PF_6_)_2_ was obtained as a red solid (105 mg, 0.07 mmol, 87%). ^1^H NMR (500 MHz, CD_3_CN) δ 8.73 (d, *J* = 8.1 Hz, 1H), 8.57 (d, *J* = 7.6 Hz, 2H), 8.39–8.17 (m, 13H), 7.84–7.73 (m, 4H), 7.65 (d, *J* = 3.7 Hz, 20H), 7.06 (d, *J* = 5.9 Hz, 1H), 6.55–6.49 (m, 2H), 2.85–2.78 (m, 2H), 2.60–2.53 (m, 1H), 2.39–2.30 (m, 1H), 1.92–1.81 (m, 2H), 1.72–1.63 (m, 2H), 1.54 (d, *J* = 11.5 Hz, 1H), 1.47–1.41 (m, 1H), 1.40–1.33 (m, 2H), 1.28 (s, 3H), 1.24–1.21 (m, 1H), 0.91 (d, *J* = 6.7 Hz, 2H). ^13^C NMR (126 MHz, CD_3_CN) δ 155.1, 154.7, 154.5, 153.2, 153.2, 153.1, 153.0, 149.8, 149.1, 149.1, 149.1, 149.1, 149.1, 149.0, 149.0, 149.0, 148.5, 138.6, 137.8, 136.2, 134.1, 133.9, 132.1, 130.5, 130.4, 130.3, 130.2, 130.2, 130.2, 130.2, 129.8, 129.7, 129.6, 129.6, 129.6, 127.8, 127.3, 127.1, 127.1, 126.8, 126.7, 126.6, 126.5, 125.4, 125.4, 125.0, 117.9, 115.6, 113.1, 110.5, 44.0, 40.1, 34.2, 33.6, 32.2, 29.9, 29.6, 27.9, 26.8, 25.2, 23.0, 14.5, 14.0. ES-HRMS calculated for *m*/*z* [M]^2+^ = 641.7086; found 641.7085.

[Ru­(DIP)_2_(**L-1c**)]­Cl_2_, (**6**). According to General Procedure C, *cis*-[Ru­(DIP)_2_Cl_2_] (75 mg, 0.09 mmol) and **L-1c** (53 mg, 0.1 mmol) were utilized. [**6**]­(PF_6_)_2_ was obtained as a red solid (112 mg, 0.07 mmol, 87%). ^1^H NMR (500 MHz, CD_3_CN) δ 8.71 (d, *J* = 8.3 Hz, 1H), 8.55 (d, *J* = 8.6 Hz, 2H), 8.32 (d, *J* = 5.0 Hz, 2H), 8.28 (s, 2H), 8.25 (d, *J* = 3.6 Hz, 2H), 8.20 (dd, *J* = 13.7, 6.0 Hz, 7H), 7.81–7.72 (m, 4H), 7.63 (s, 20H), 7.13 (d, *J* = 7.0 Hz, 1H), 6.64 (d, *J* = 11.3 Hz, 2H), 3.71 (s, 3H), 2.83 (s, 2H), 2.54 (s, 1H), 2.33 (d, *J* = 19.9 Hz, 1H), 2.08 (d, *J* = 6.0 Hz, 1H), 1.54 (ddd, *J* = 74.8, 30.8, 17.9 Hz, 8H), 1.22–1.17 (m, 2H), 1.08 (s, 3H). ^13^C NMR (126 MHz, CD_3_CN) δ 158.4, 158.3, 156.0, 156.0, 155.1, 154.9, 153.5, 153.4, 150.2, 150.2, 149.5, 149.4, 149.4, 149.4, 148.8, 148.8, 144.4, 138.9, 138.1, 136.6, 134.5, 134.5, 134.2, 134.2, 133.3, 130.7, 130.7, 130.6, 130.1, 129.9, 129.9, 129.9, 128.1, 127.6, 127.5, 127.1, 127.0, 126.9, 126.9, 126.9, 125.8, 125.7, 125.3, 125.3, 125.3, 114.5, 114.5, 112.2, 110.9, 83.1, 55.6, 48.3, 40.4, 38.5, 33.9, 30.8, 30.4, 30.3, 29.7, 28.3, 27.2, 24.3, 14.8. ES-HRMS calculated for *m*/*z* [M]^2+^ = 648.7164; found 648.7151.

[Ru­(DIP)_2_(**L-1d**)]­Cl_2_, (**7**). According to General Procedure C, *cis*-[Ru­(DIP)_2_Cl_2_] (75 mg, 0.09 mmol) and **L-1d** (59 mg, 0.1 mmol) were utilized. [**7**]­(PF_6_)_2_ was obtained as a red solid (135 mg, 0.08 mmol, 88%). ^1^H NMR (500 MHz, CD_3_CN) δ 8.73 (d, *J* = 7.8 Hz, 1H), 8.57 (d, *J* = 8.7 Hz, 2H), 8.34 (d, *J* = 5.3 Hz, 2H), 8.31 (d, *J* = 6.6 Hz, 2H), 8.28 (dd, *J* = 5.5, 2.3 Hz, 2H), 8.22 (ddd, *J* = 14.7, 9.0, 5.3 Hz, 7H), 7.79 (ddd, *J* = 17.4, 8.4, 5.6 Hz, 4H), 7.67–7.63 (m, 21H), 7.12 (s, 1H), 6.65–6.58 (m, 2H), 4.75 (s, 1H), 2.84 (s, 2H), 2.56 (d, *J* = 4.7 Hz, 1H), 2.38 (d, *J* = 7.6 Hz, 1H), 2.11–2.09 (m, 1H), 1.84 (dd, *J* = 10.7, 8.2 Hz, 2H), 1.77–1.35 (m, 14H), 1.23 (t, *J* = 7.6 Hz, 2H), 1.10 (s, 3H). ^13^C NMR (126 MHz, CD_3_CN) δ 156.8, 156.0, 156.0, 155.1, 155.1, 154.9, 153.5, 153.5, 153.4, 150.2, 150.2, 149.5, 149.4, 149.4, 149.4, 149.4, 138.9, 138.1, 136.6, 134.7, 134.5, 133.0, 130.7, 130.6, 130.1, 129.9, 129.9, 127.6, 127.5, 127.1, 127.0, 127.0, 126.9, 125.8, 125.3, 116.3, 113.8, 110.9, 83.1, 79.7, 79.1, 55.3, 48.3, 44.4, 40.4, 38.5, 34.6, 33.4, 32.6, 32.5, 30.8, 30.4, 30.3, 30.3, 30.2, 30.1, 29.9, 29.7, 28.3, 27.2, 27.1, 25.6, 24.6, 24.3, 23.3, 14.8, 14.3. ES-HRMS calculated for *m*/*z* [M]^2+^ = 675.7399; found 675.7400.

### Partition Coefficients

The logP values for compounds **1**–**7** were determined using the shake-flask method.^53^ The octanol phase used in this experiment was presaturated with Tris-HCl buffer (0.05 M, pH = 7.4) by overnight stirring of the biphasic mixture of the two at room temperature, and similarly, the aqueous phase was presaturated with octanol. For complexes **1**–**4** and for [Ru­(bpy)_3_]­Cl_2_, 5 mL of a 50 μM solution of each complex in the aqueous phase was added to 5 mL of the octanol phase. For complexes **5**–**7**, 5 mL of a 50 μM solution of each complex in the octanol phase was added to 5 mL of the aqueous phase. Each mixture was agitated for 24 h in the dark. The layers were then separated, and the absorbance at 450 nm in each phase was measured. The logP values were calculated based on the following equation:

$$\log P=\log\left(\frac{{\mathrm{Absorbance}}_{450}\left(\mathrm{Octanol}\right)}{{\mathrm{Absorbance}}_{450}\left(\mathrm{Tris}‐\mathrm{HCl}\;\mathrm{buffer}\right)}\right)$$

### DLS Analysis

The particle size distribution of the complexes was determined by dynamic light scattering (DLS) using a Particle Analyzer Litesizer 500 (Anton Paar, Austria) at 25 °C. 20 μM solutions of complexes **1**–**4** and of [Ru­(bpy)_3_]­Cl_2_ in Tris-HCl buffer, as well as 20 μM solutions of complexes **1**–**4** in Gibco DMEM (Dulbecco’s Modified Eagle Medium with high glucose, l-glutamine, pyruvate, 10% FBS, and without phenol red), were analyzed by DLS. The experiments were performed in triplicate, yielding similar results.

### ROS Generation Studies

a) ABDA assay: Stock solutions of 9,10-anthracenediyl-bis­(methylene)­dimalonic acid (ABDA) and the respective complex were combined in PBS in a 96-well plate so that the final concentrations were 100 and 10 μM, respectively. The absorbance of the whole plate was measured using a BMG LabTech Clariostar plate reader. The plate was then irradiated for 30 s using a Lumidox 457 nm 96-LED array set to 8 mA (2 mW/cm²), shaken for 30 s, and the absorbance was remeasured. This process was repeated for the following time points: 30, 60, 120, and 180 s. The experiment was repeated in triplicate and averaged. The absorbance at 380 nm corresponding to the ABDA maxima was extracted and fitted to a linear regression (Figure S56). The gradients were then normalized to that of [Ru­(bpy)_3_]^2+^. b) DHR-123: A 20 μM solution of each complex in PBS was prepared in each plate, before being diluted 1:10 in a new plate to obtain 200 μL of a 2 μM solution. 50 μL of this solution was combined with 50 μL of a 20 μM DHR-123 stock solution so that the final concentration in each well was 1 μM complex and 10 μM DHR-123. The plate was irradiated under the same conditions used for the ABDA assay, but this time the DHR-123 emission in each well was measured using λ_ex_ = 505 nm and λ_em_ = 535 nm at various time points (0–3 min). The experiment was repeated in triplicate and averaged. The DHR-123 emission at 535 nm was plotted over time and fitted to a linear regression (Figure S57). The gradients were then normalized to that of [Ru­(bpy)_3_]^2+^.

### Tissue Culture

MDA-MB-453 and MCF-7 cells were grown in high-glucose Dulbecco’s Modified Eagle Medium (DMEM) containing 10% fetal bovine serum at 37 °C with 5% CO_2_ in humidified air.

### Viability Experiments

For viability experiments, cells were seeded at a density of 20,000 cells per well in Greiner-Bio black μClear plates. After 24 h, the corresponding ruthenium complexes were added at the appropriate concentrations (30 nM to 100 μM for complexes **1**-**4** and 30 nM to 20 μM for **5**–**7**, < 1% DMSO) and allowed to incubate. For the phototoxicity experiments, the plate was removed at the six-hour time point and irradiated for 12 min using a Lumidox 457 nm 96-LED array set to 8 mA (2 mW/cm^2^, a total of 1.2 J/s). After an overall 24 h incubation period, the media were replaced with fresh media containing an MTS/PMS mixture as per the Promega protocol (MTS: 3-(4,5-dimethylthiazol-2-yl)-5-(3-carboxymethoxyphenyl)-2-(4-sulfophenyl)-2H-tetrazolium; PMS: phenazine methosulfate). After an additional 4 h had passed, the absorbance at 490 nm (MTS) and 635 nm (background) was measured. Cell viability was calculated from the dose-response curve of the absorbance (MTS – background).

### Microscopy

For imaging experiments, cells were seeded at a density of 20,000 cells per well in Greiner-Bio black μClear plates. After 24 h, the corresponding ruthenium complexes were added at the appropriate concentrations. After 6 h, the cells were washed 3 times with media, and the media were replaced with phenol-red-free media. Compounds were imaged on an Eclipse-Ti2 Widefield system (Nikon), equipped with a Prime 95B sCMOS camera (Teledyne), pE-400 LED light source (CoolLED), a 20× or 40× objective, and emission filters for green (515/30 nm), orange (600/50 nm), red (641/75 nm), and far-red (705/72 nm). The bright-field channel was used to autofocus each well, after which the complexes were excited using a 450 nm laser and the red emission channel collected.

### ICP-MS Analysis

Cells were seeded at the appropriate density in 6-well plates (1 × 10^6^ cells per well for MDA-MB-453 and 400,000 cells per well for MCF-7) and allowed to adhere for 24 h. Ruthenium complexes were added to the wells by direct dilution of a 10 mM DMSO stock solution to yield a final concentration of 20 μM in culture medium. After 6 h of incubation, the medium was removed, and the cells were washed three times with phosphate-buffered saline (PBS) to eliminate extracellular ruthenium. Cells were subsequently detached using trypsin, collected by centrifugation, and counted. Cell pellets were digested in 1 mL of 70% trace metal-grade nitric acid by incubation at 60 °C overnight. After digestion, 0.5 mL of the sample was diluted with 9.5 mL of deionized water and passed through a polytetrafluoroethylene (PTFE) syringe filter. All ICP-MS measurements were performed on an 8900 ICP-MS Triple Quad. An indium internal standard was injected after inline mixing with the samples to correct for signal drift and matrix effects. The monitored isotope was 101Ru. A set of ruthenium calibration standards was used to establish and model the linear relationship between signal and concentration before the samples were injected, signal measured, and averaged across three consecutive signal acquisitions. The amount of metal detected in the cell samples was normalized to ppb per 1,000,000 cells.

## Supplementary Material



## Notes

Additional experimental details including general synthetic pathways, NMR and MS analysis of the synthesized compounds, detailed photophysical properties, LC-MS data, ROS generation, cytotoxicity (dark and upon irradiation) results, microscopy images (PDF)
