# Supplementary material for: Estrogen-Functionalized Ru(II) Polypyridyl Complexes Self-Assemble into Aggregates and Exhibit Selective Phototoxicity against Breast Cancer Cells
Source: Inorg Chem. 2025 Nov 5;64(45):22340–54. doi: 10.1021/acs.inorgchem.5c03244 (PMC12628291; doi:10.1021/acs.inorgchem.5c03244)
Supplement: Supplementary file 1 [file ic5c03244_si_001.pdf]

Supporting Information

Estrogen-functionalized Ru(II) polypyridyl complexes self-assemble into aggregates and exhibit selective phototoxicity against breast cancer cells

Sofia Alexandra Tsoni,[a] Timothy Kench,[b] Ramon Vilar, *[b] and Theodore Lazarides*[a]

[a] Department of Chemistry, Aristotle University of Thessaloniki, 54124 Thessaloniki, Greece E-mail: tlazarides@chem.auth.gr

[b] Department of Chemistry, Imperial College London, White City Campus, W120BZ London, UK E-mail: r.vilar@imperial.ac.uk

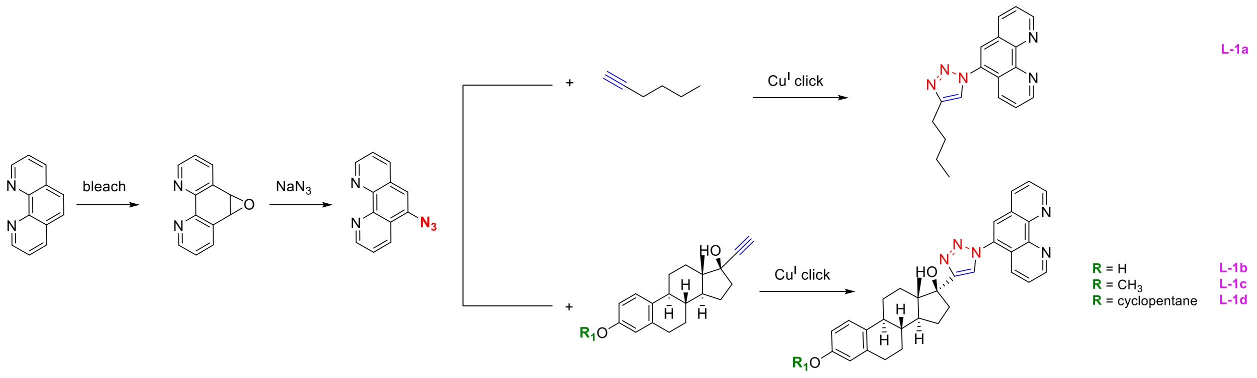

Scheme S 1. Synthetic route for ligands **L-1a**–**L-1d**.

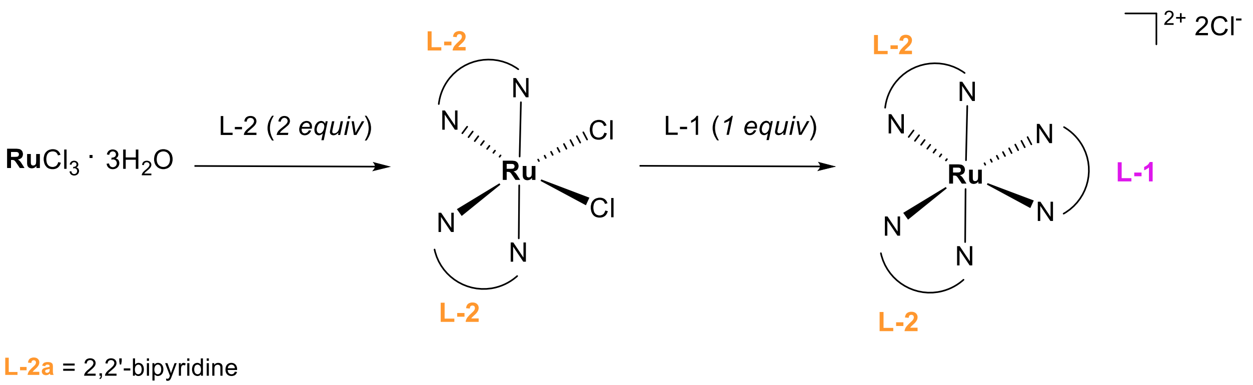

L-2a = 2,2'-bipyridine
L-2b = 4,7-diphenyl-1,10-phenanthroline

Scheme S 2. Synthetic route for Complexes **1**–**7**.

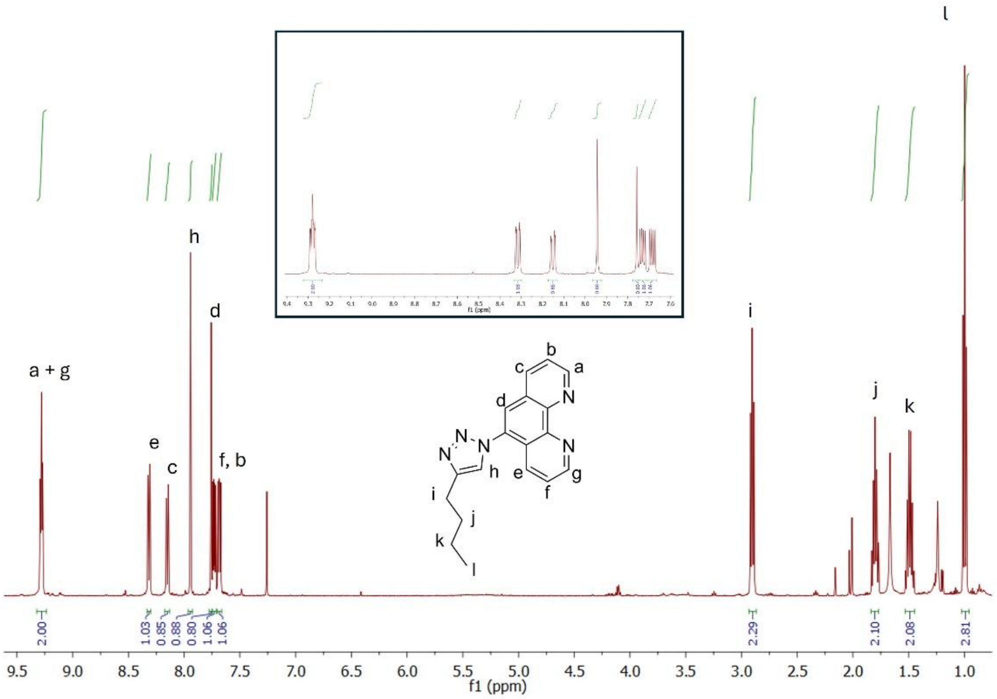

Figure S 1. ^{1}H-NMR spectrum of **L-1a**; inset: expanded aromatic region.

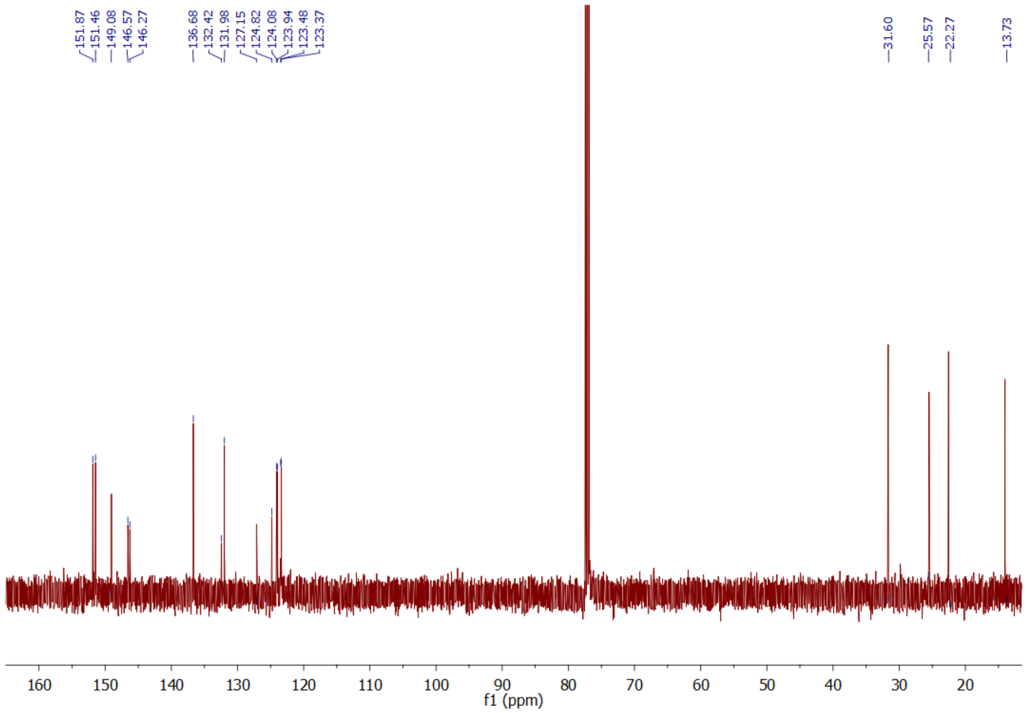

Figure S 2. ^{13}C-NMR spectrum of **L-1a**.

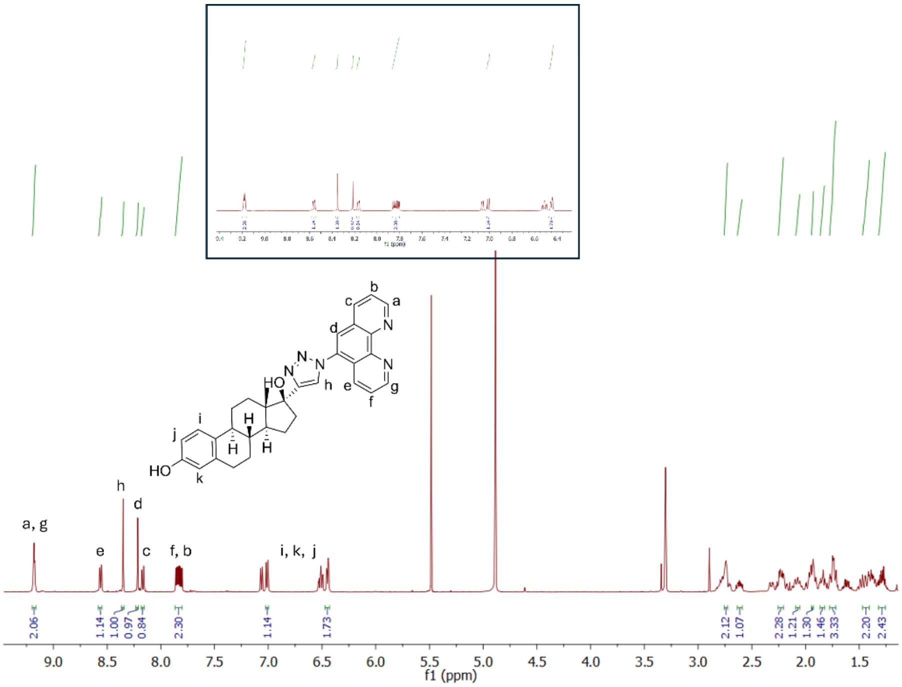

Figure S 3 . ^{1}H-NMR spectrum of **L-1b**; inset: expanded aromatic region.

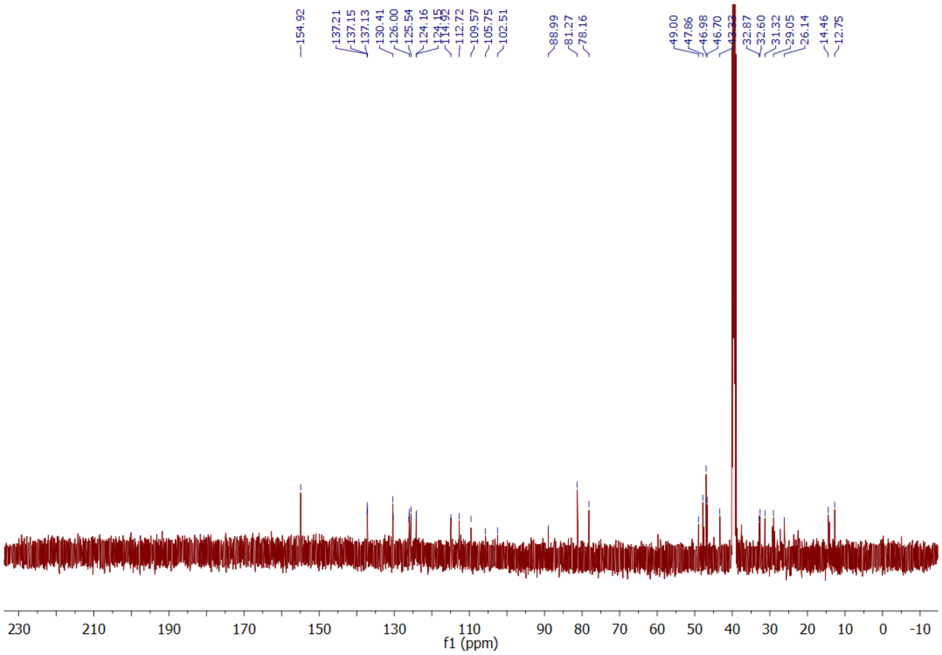

Figure S 4. ^{13}C-NMR spectrum of **L-1b**.

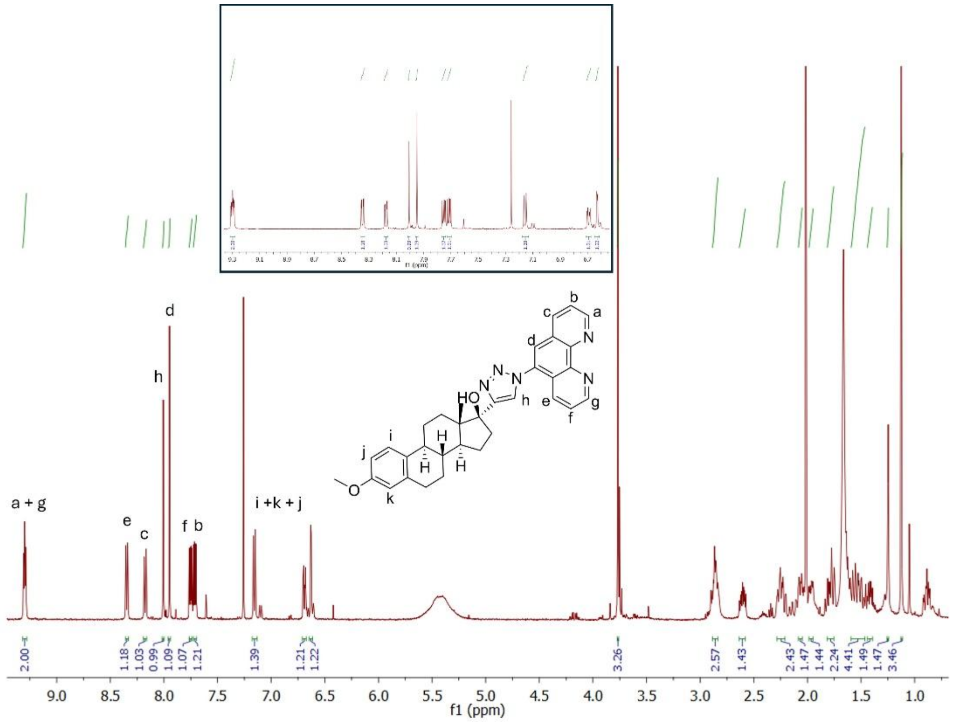

Figure S 5. ^{1}H-NMR spectrum of **L-1c**; inset: expanded aromatic region.

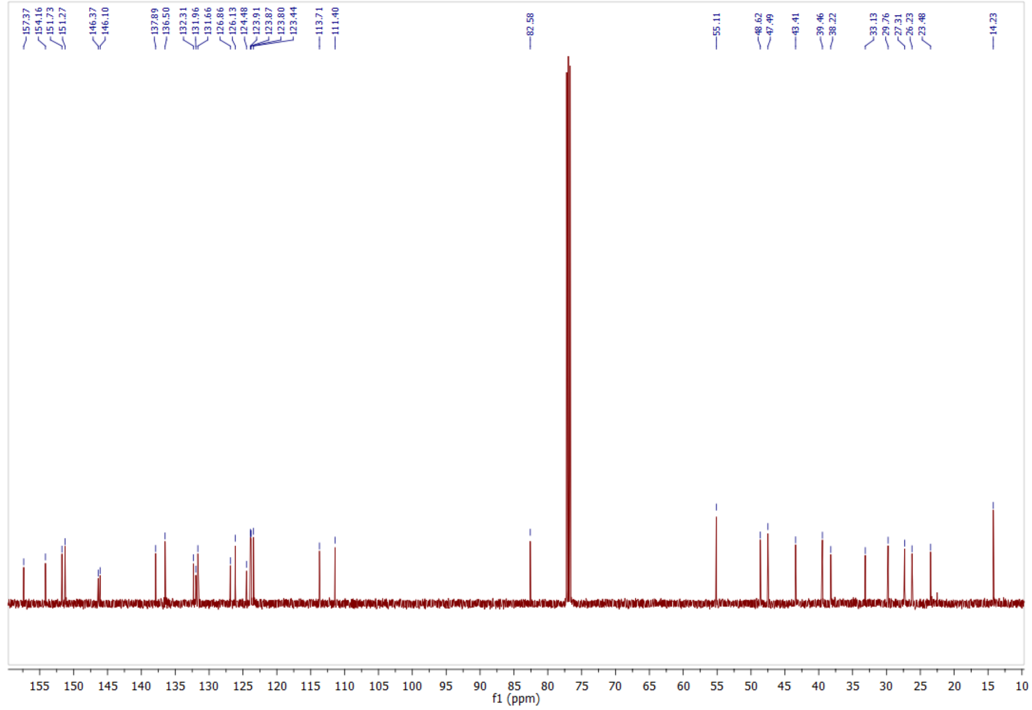

Figure S 6. ^{13}C-NMR spectrum of **L-1c**.

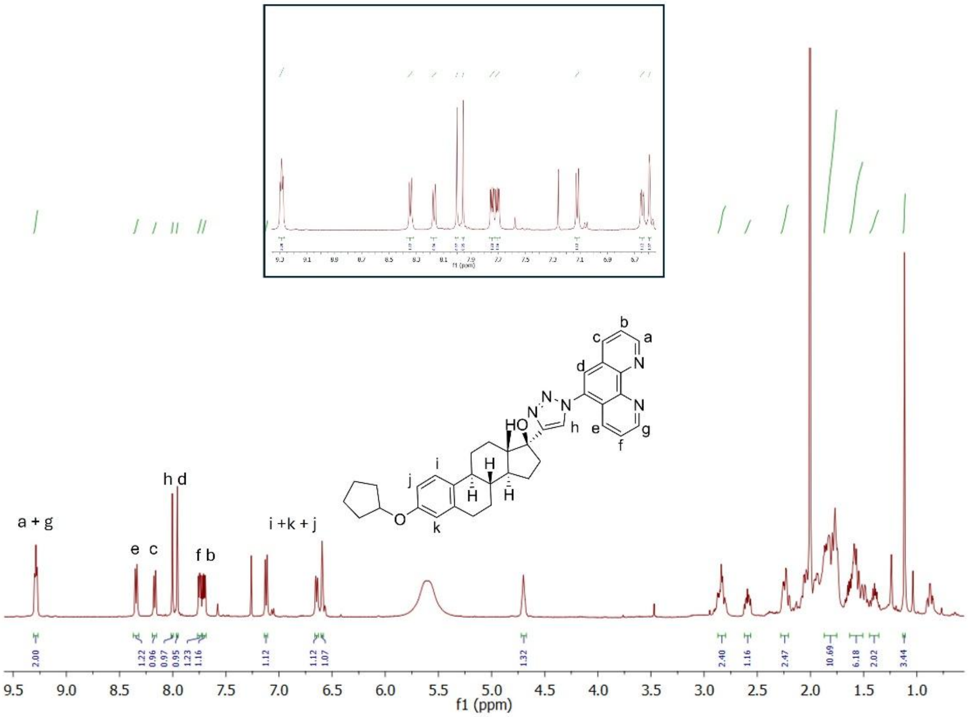

Figure S 7. ^{1}H-NMR spectrum of **L-1d**; inset: expanded aromatic region.

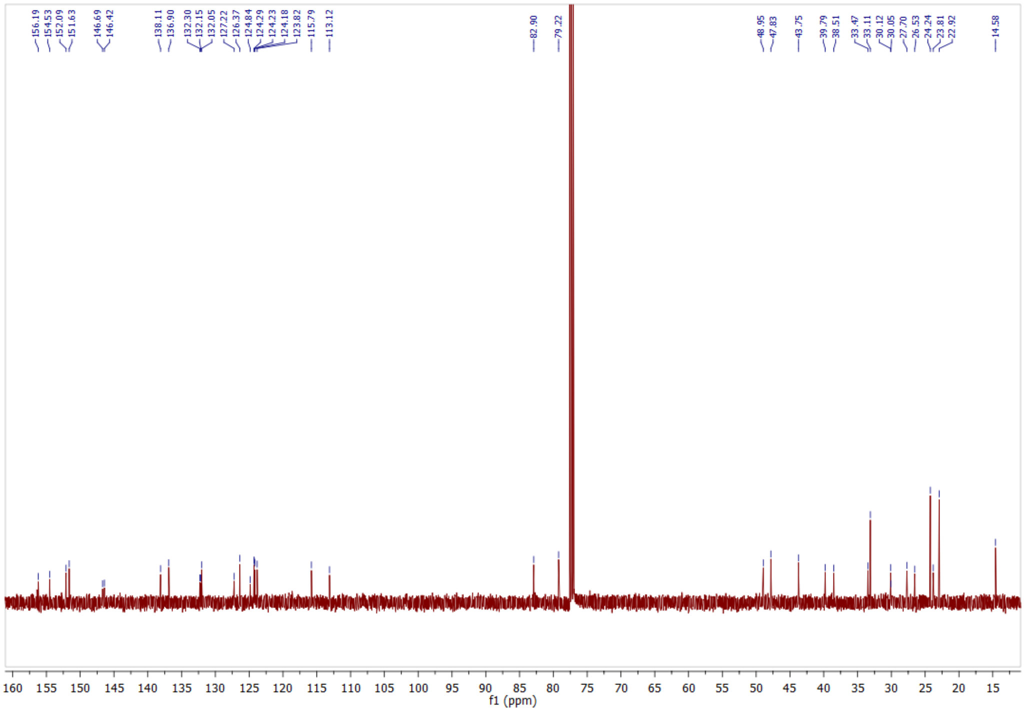

Figure S 8. ^{13}C-NMR spectrum of **L-1d**.

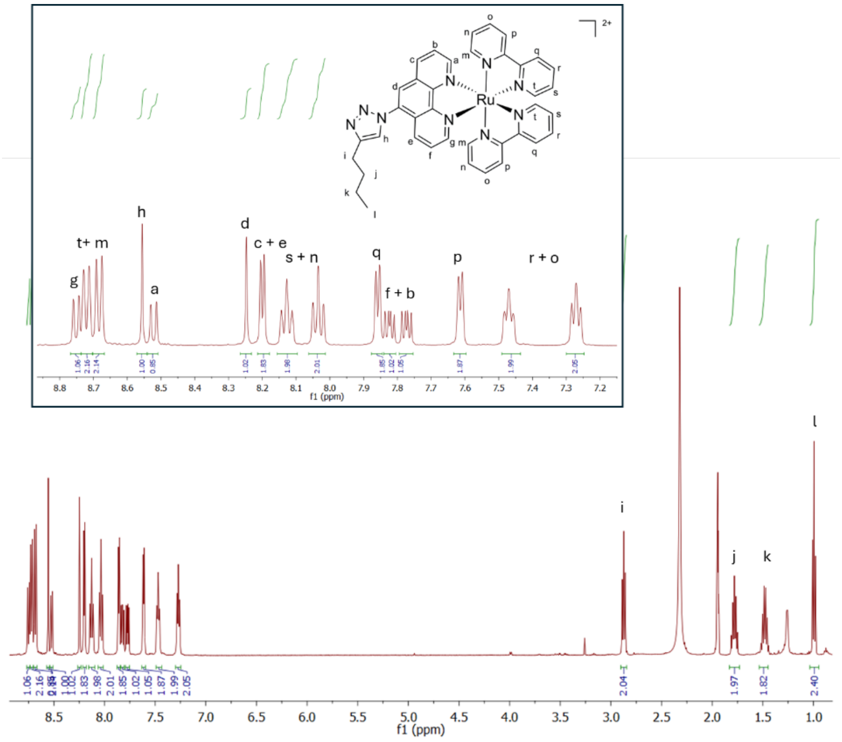

Figure S 9. ^{1}H-NMR spectrum of Complex **1**; inset: expanded aromatic region.

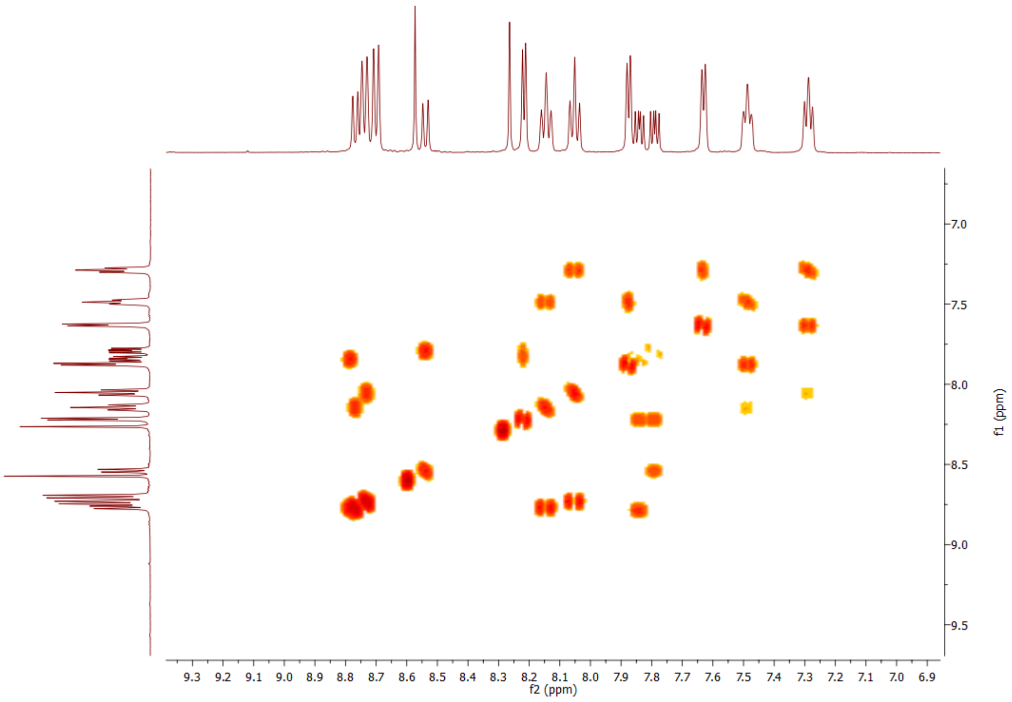

Figure S 10. ^{1}H-^{1}H COSY NMR spectrum of Complex **1**, zoomed in the aromatic region.

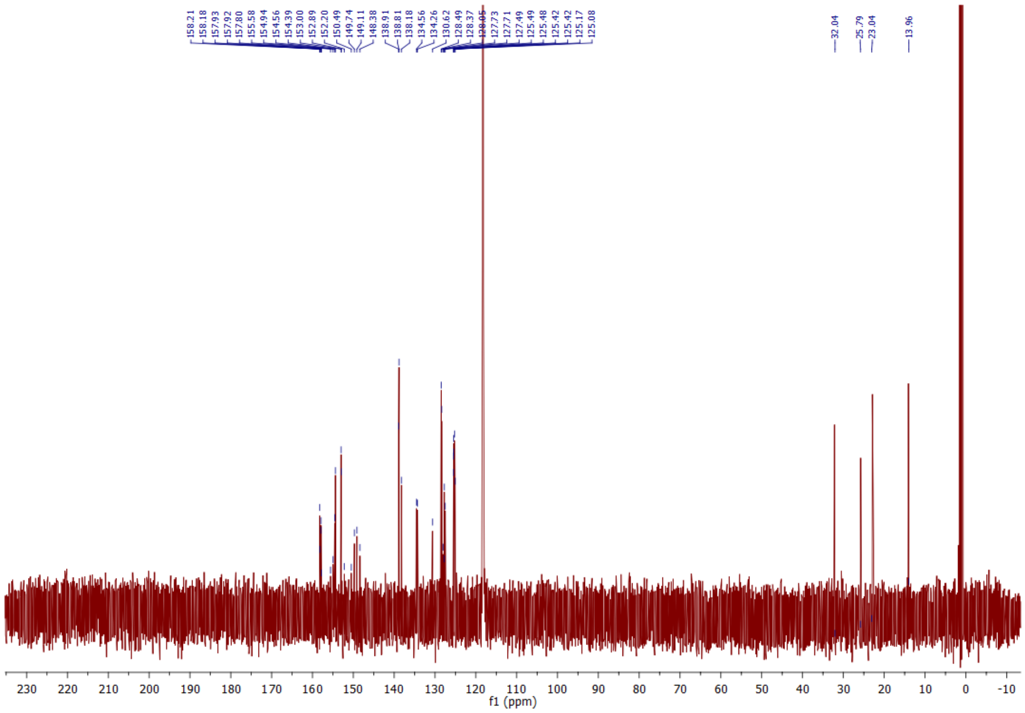

Figure S 11. ^{13}C-NMR spectrum of Complex **1**.

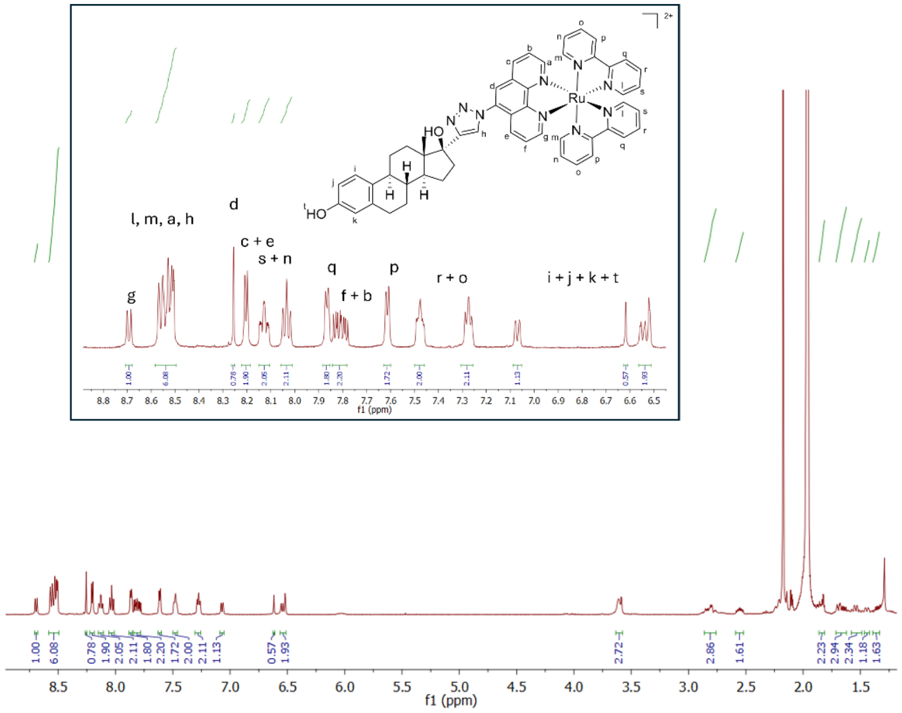

Figure S 12. ^{1}H-NMR spectrum of Complex **2**; inset: expanded aromatic region.

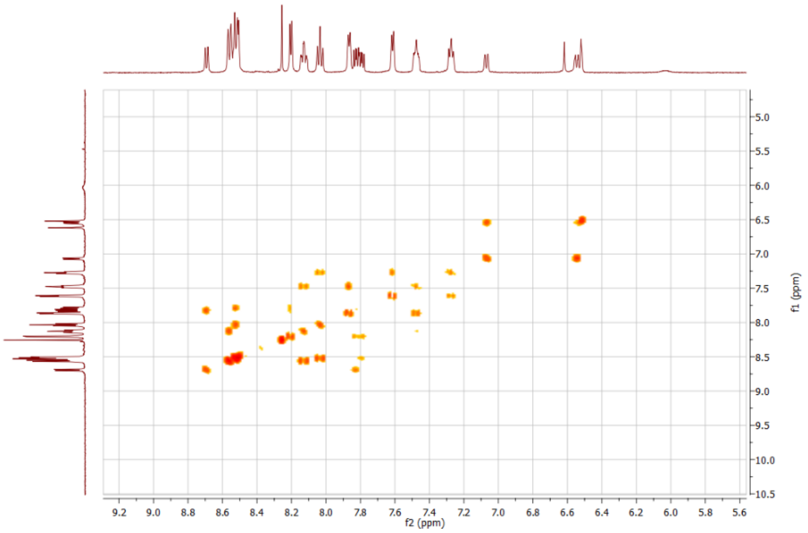

Figure S 13. ^{1}H-^{1}H COSY NMR spectrum of Complex **2**, zoomed in the aromatic region.

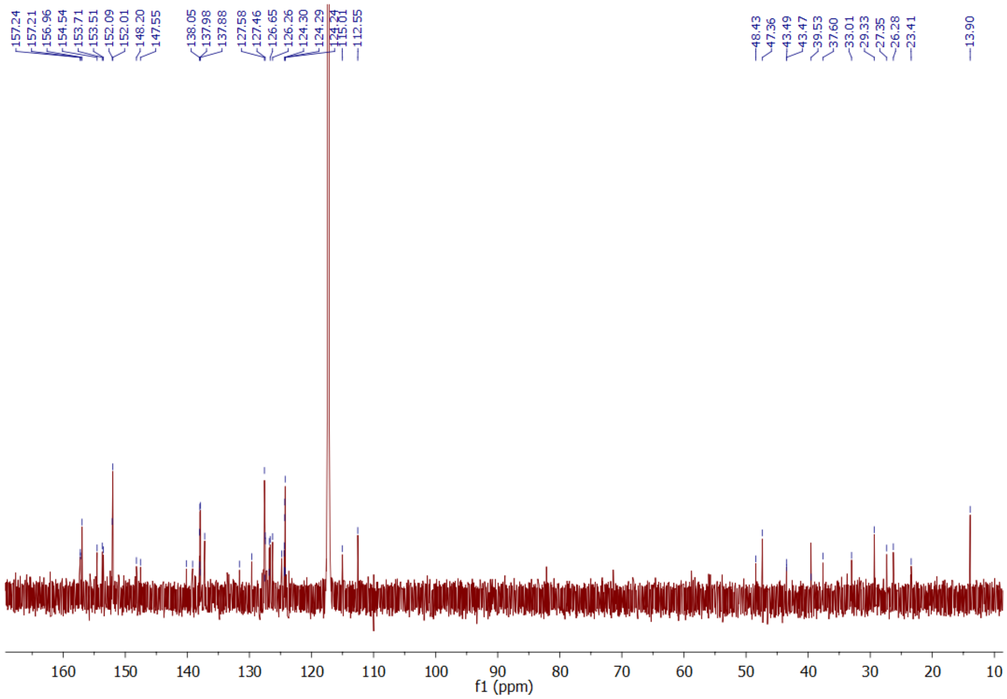

Figure S 14. ^{13}C-NMR spectrum of Complex **2**.

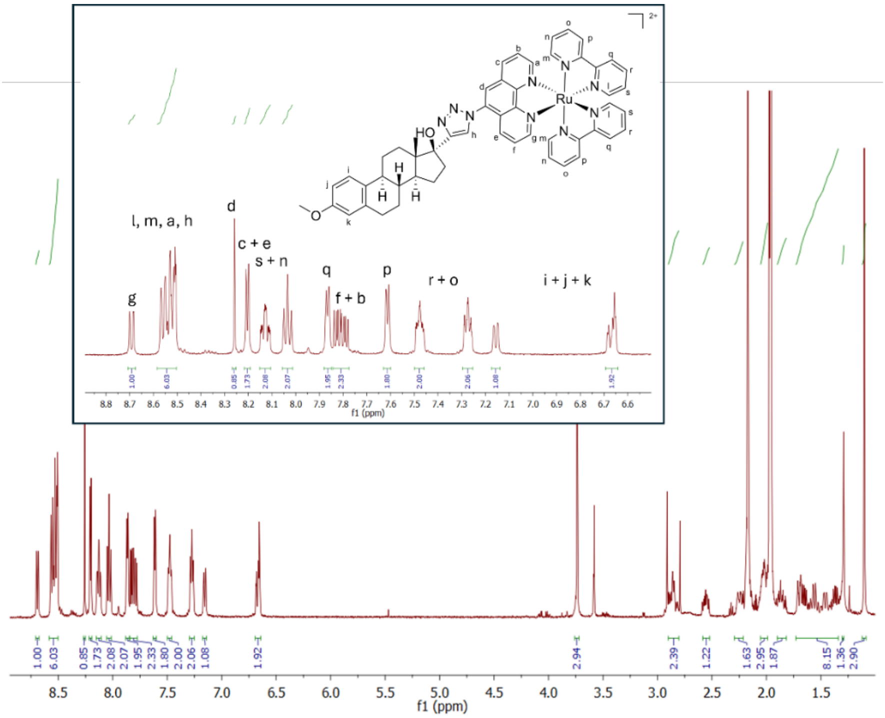

Figure S 15. ^{1}H-NMR spectrum of Complex **3**; inset: expanded aromatic region.

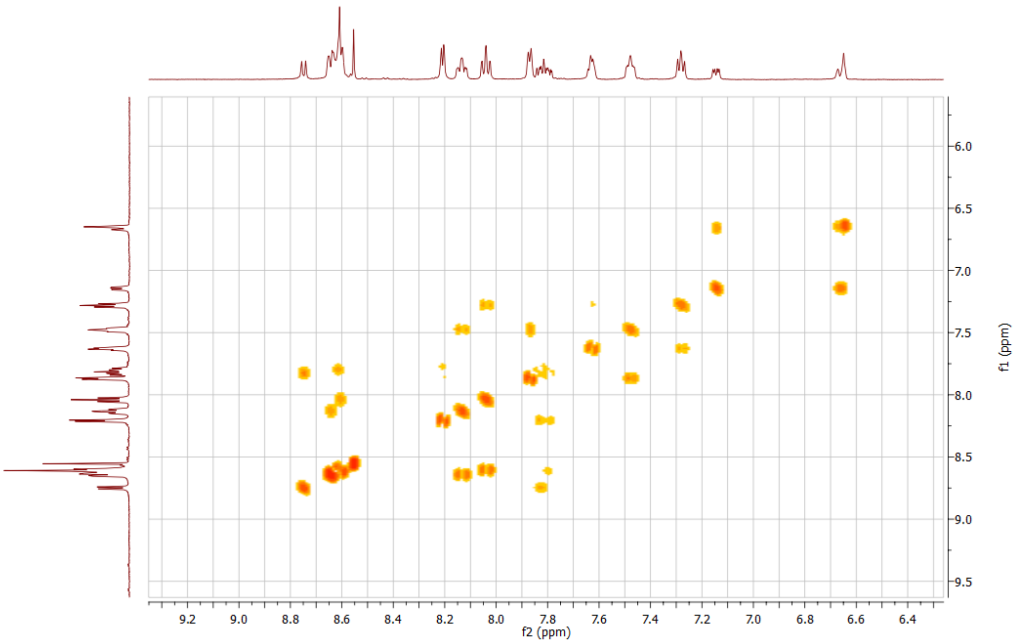

Figure S 16. ^{1}H-^{1}H COSY NMR spectrum of Complex **3**, zoomed in the aromatic region.

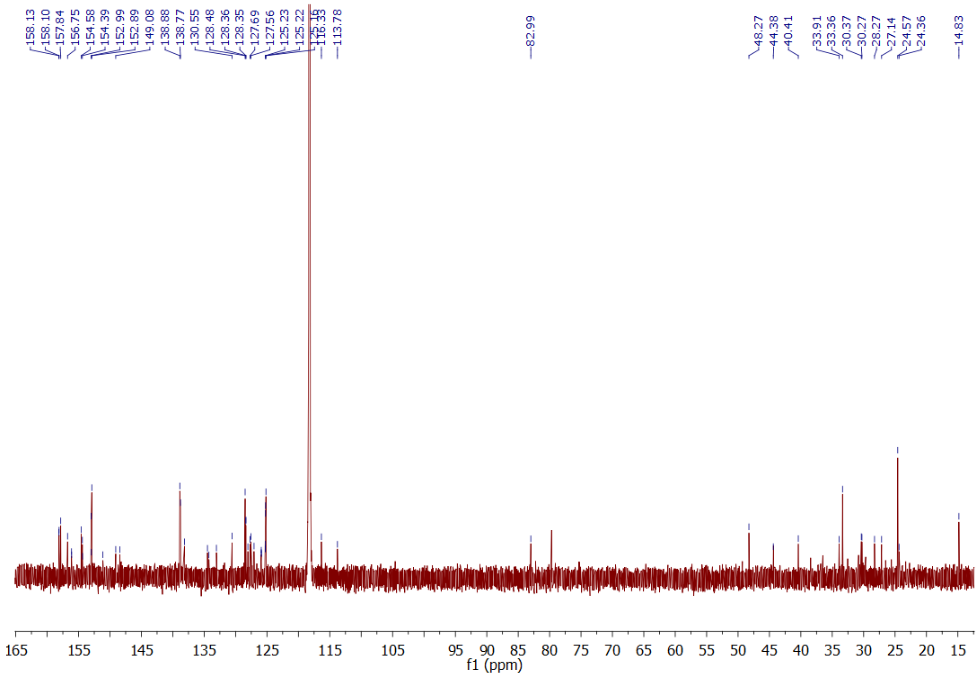

Figure S 17. ^{13}C-NMR spectrum of Complex **3**.

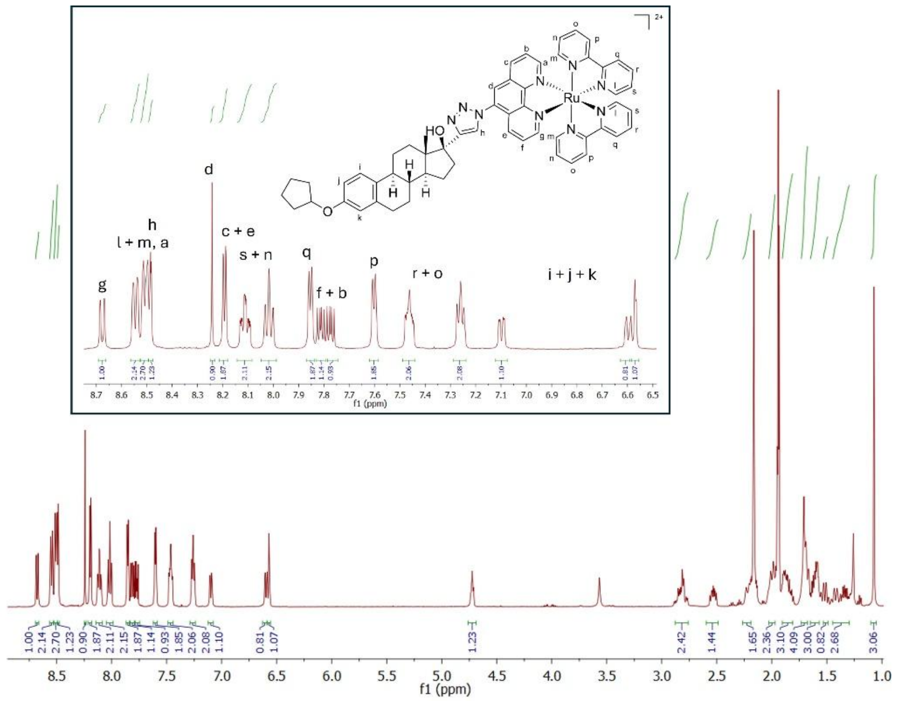

Figure S 18. ^{1}H-NMR spectrum of Complex **4**; inset: expanded aromatic region.

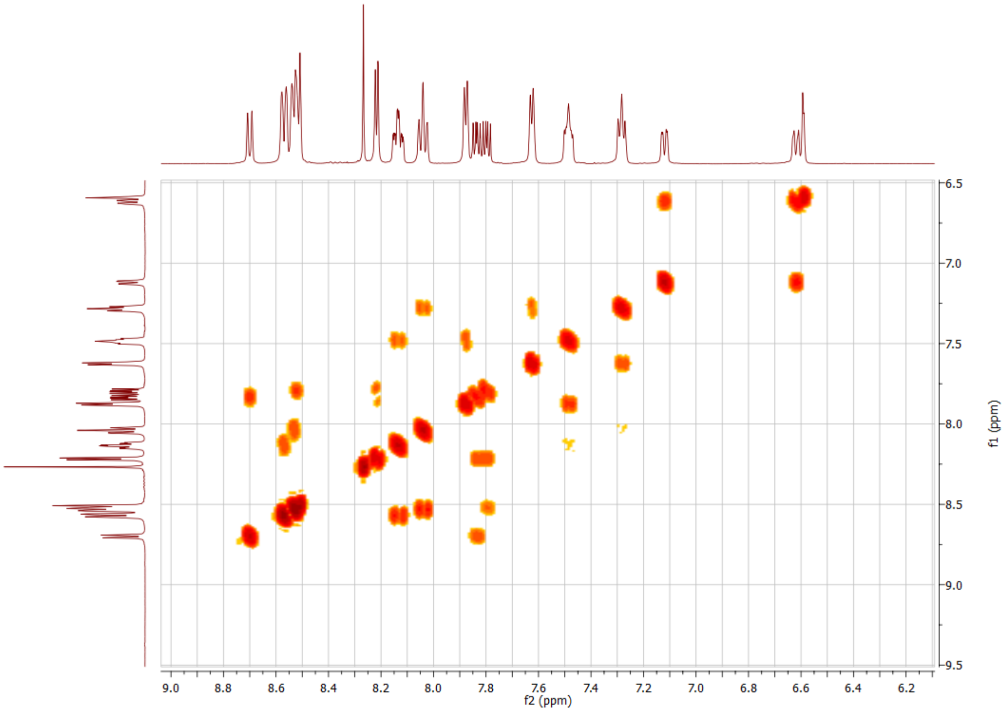

Figure S 19. ^{1}H-^{1}H COSY NMR spectrum of Complex **4**, zoomed in the aromatic region.

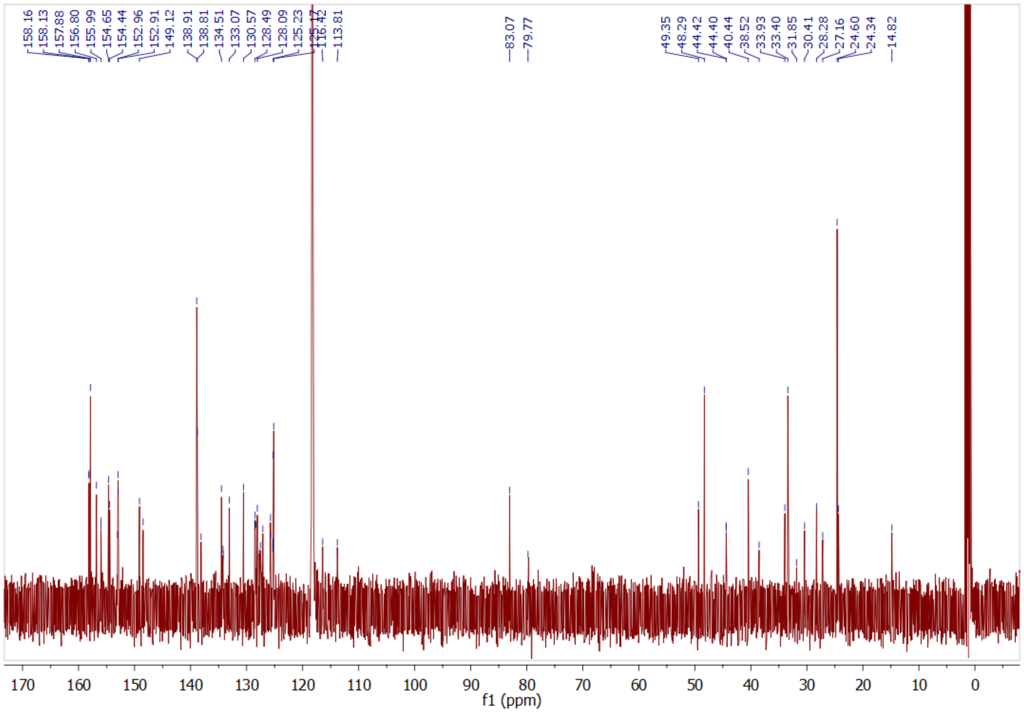

Figure S 20. *^{13}C-NMR spectrum of Complex **4**.*

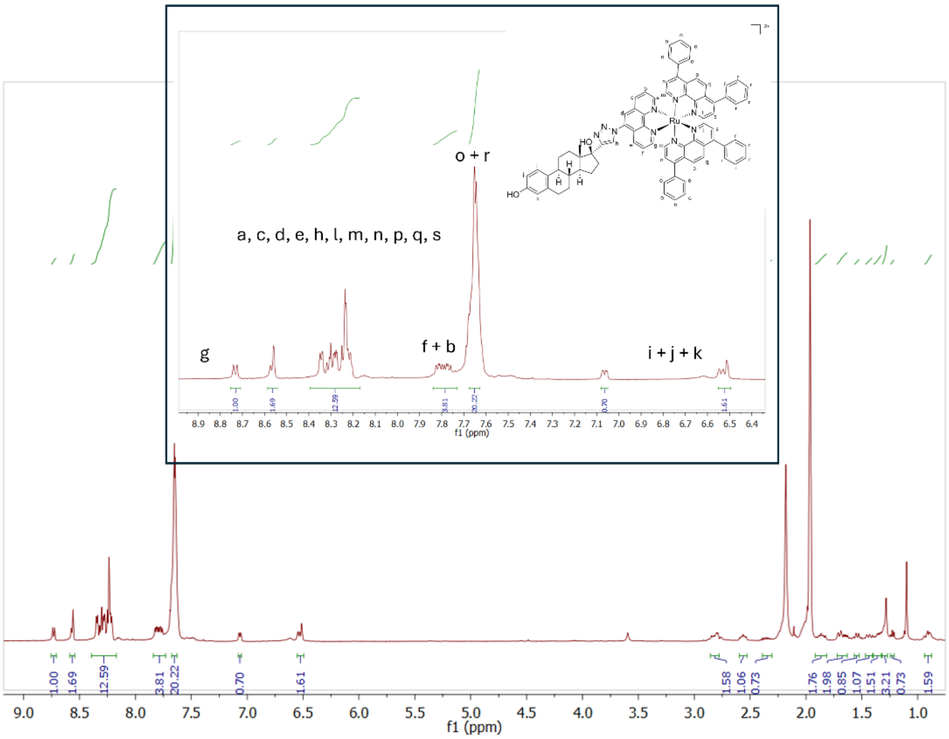

Figure S 21. ^{1}H-NMR spectrum of Complex **5**; inset: expanded aromatic region.

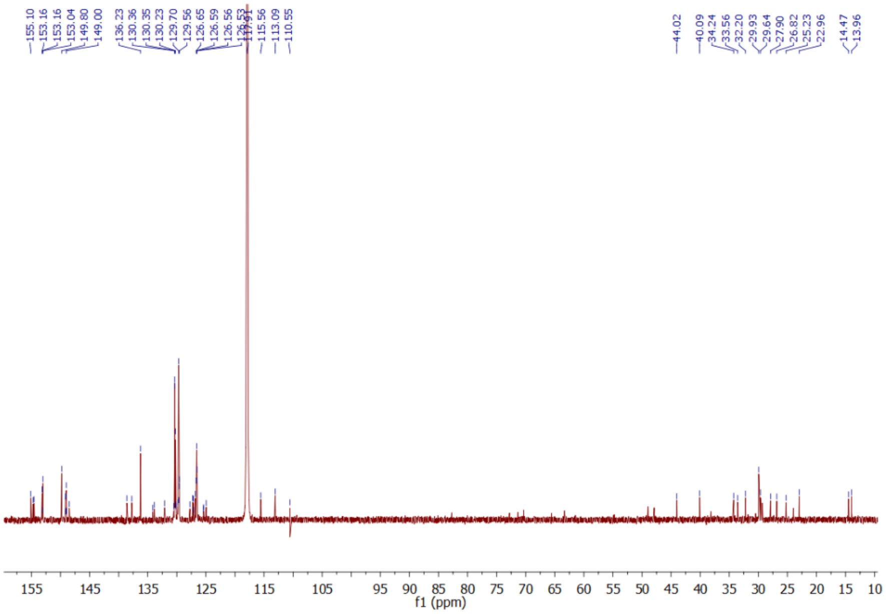

Figure S 22. *^{13}C-NMR spectrum of Complex* ***5***

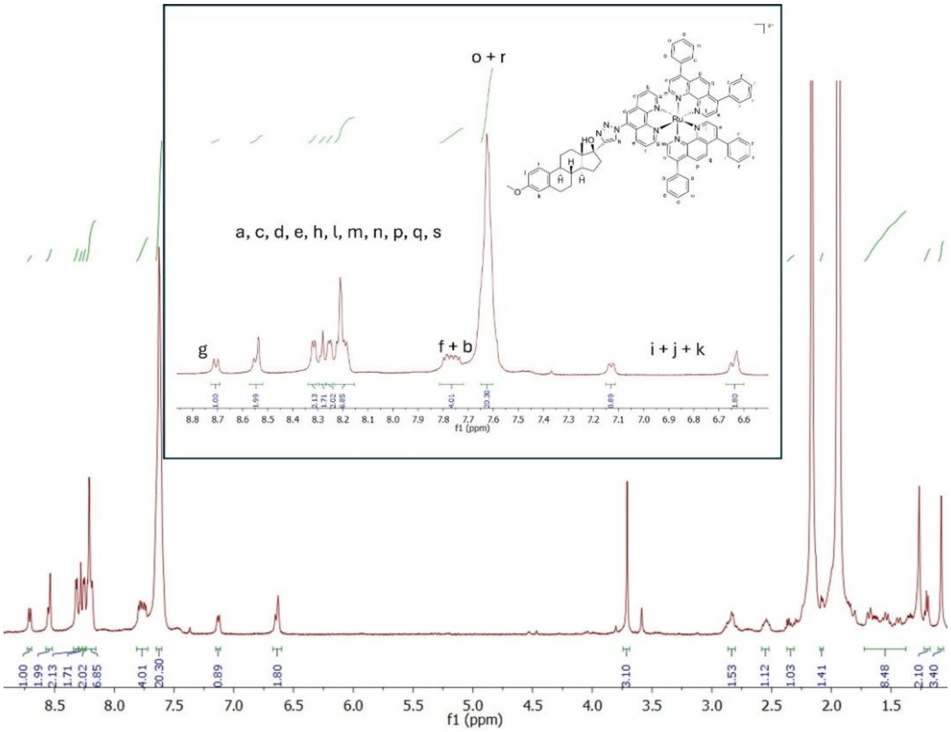

Figure S 23. ^{1}H-NMR spectrum of Complex **6**; inset: expanded aromatic region.

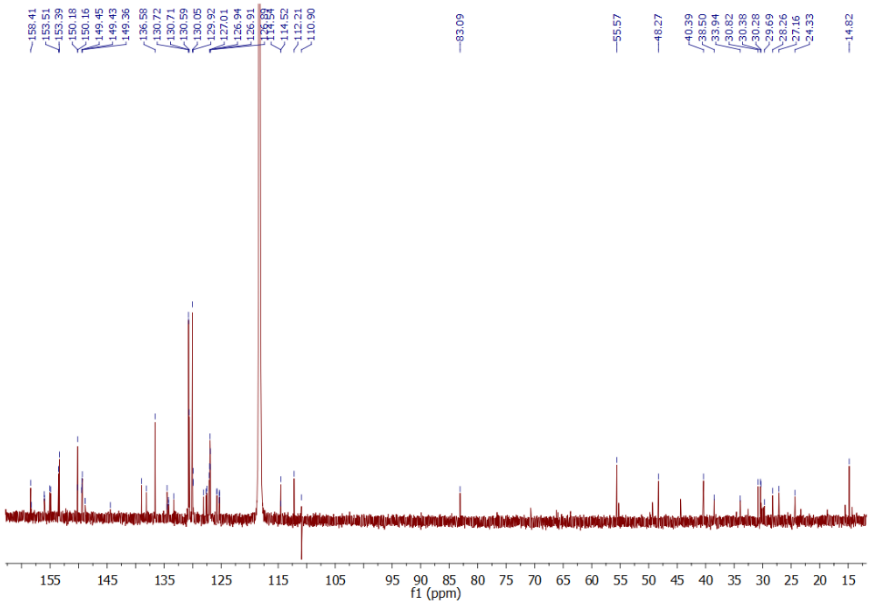

Figure S 24. *^{13}C-NMR spectrum of Complex* ***6***

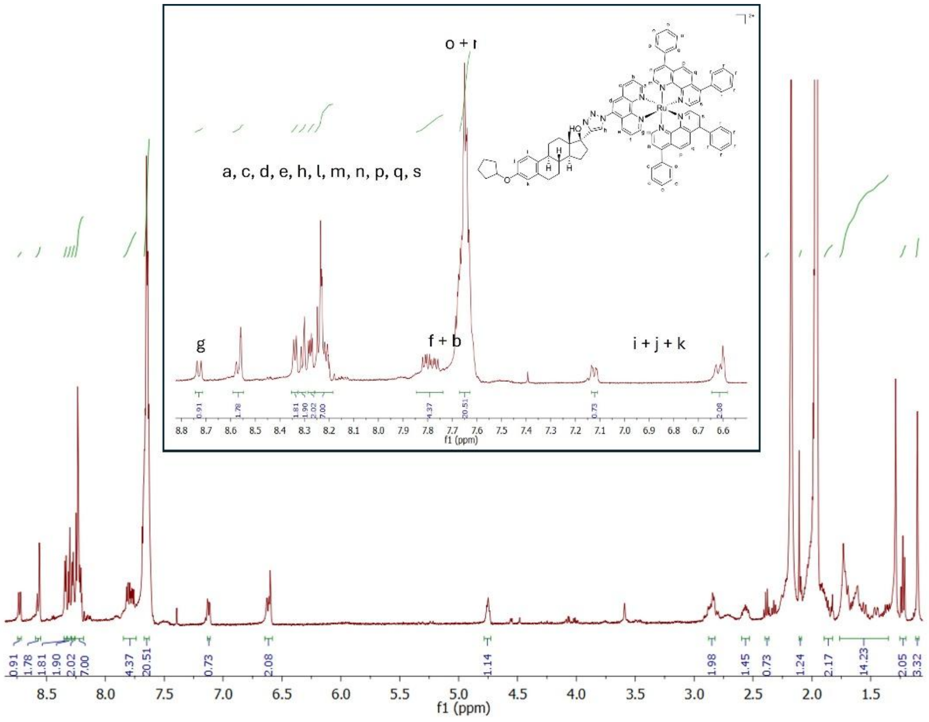

Figure S 25. ^{1}H-NMR spectrum of Complex **7**; inset: expanded aromatic region.

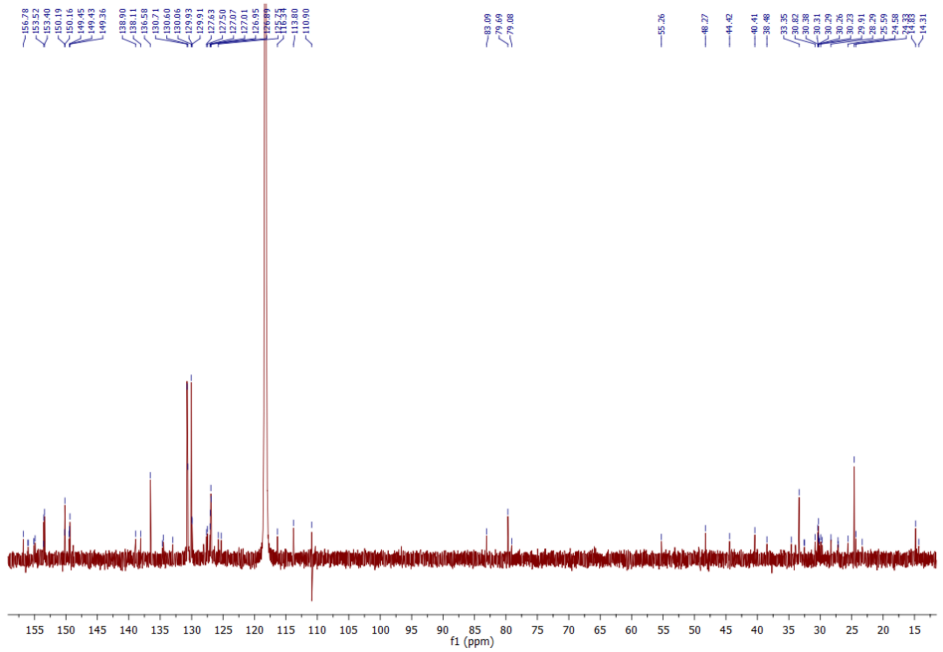

Figure S 26. *^{13}C-NMR spectrum of Complex **7***

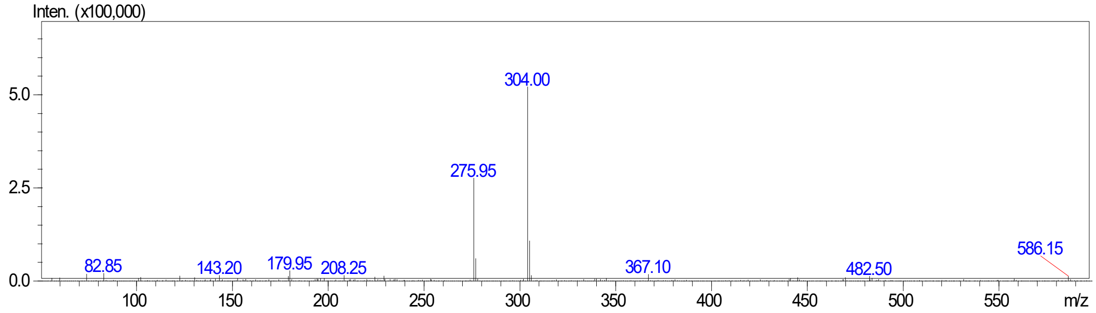

Figure S 27. ESI-MS spectrum of **L-1a** (positive detection mode).

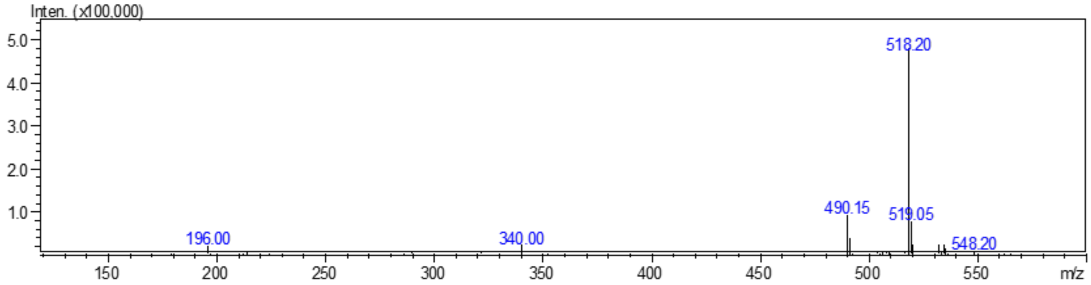

Figure S 28. ESI-MS spectrum of **L-1b** (positive detection mode).

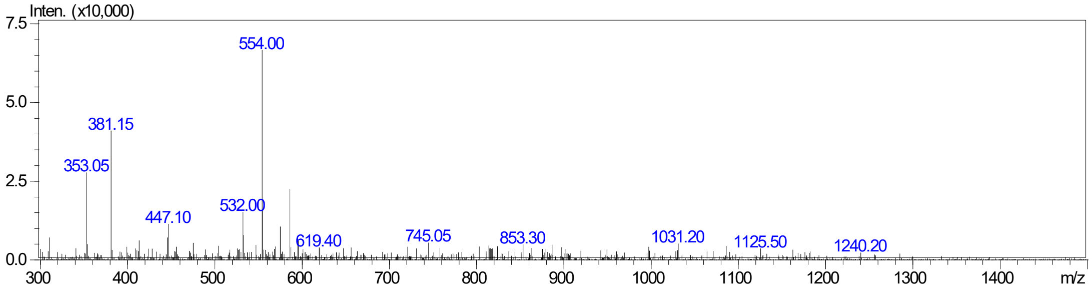

Figure S 29. ESI-MS spectrum of **L-1c** (positive detection mode).

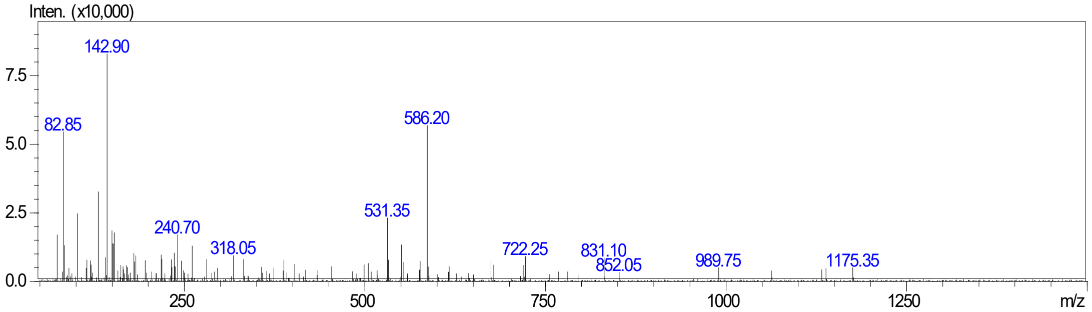

Figure S 30. ESI-MS spectrum of **L-1d** (positive detection mode).

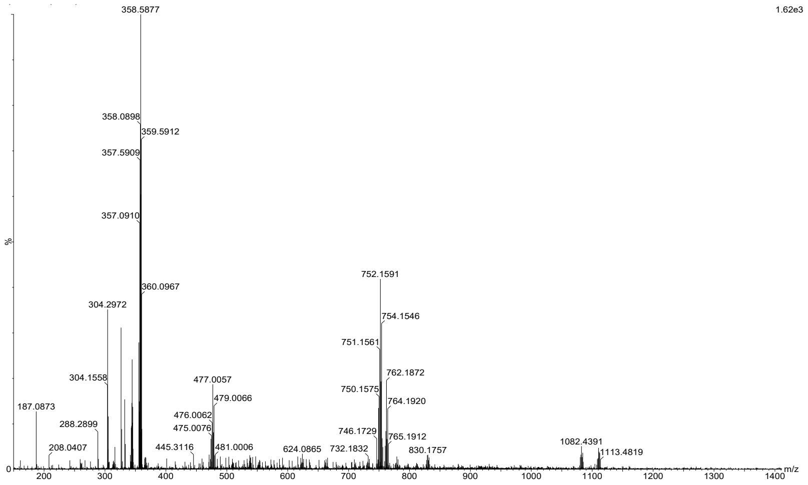

Figure S 31. TOF ESI-MS spectrum for Complex **1** (positive detection mode).

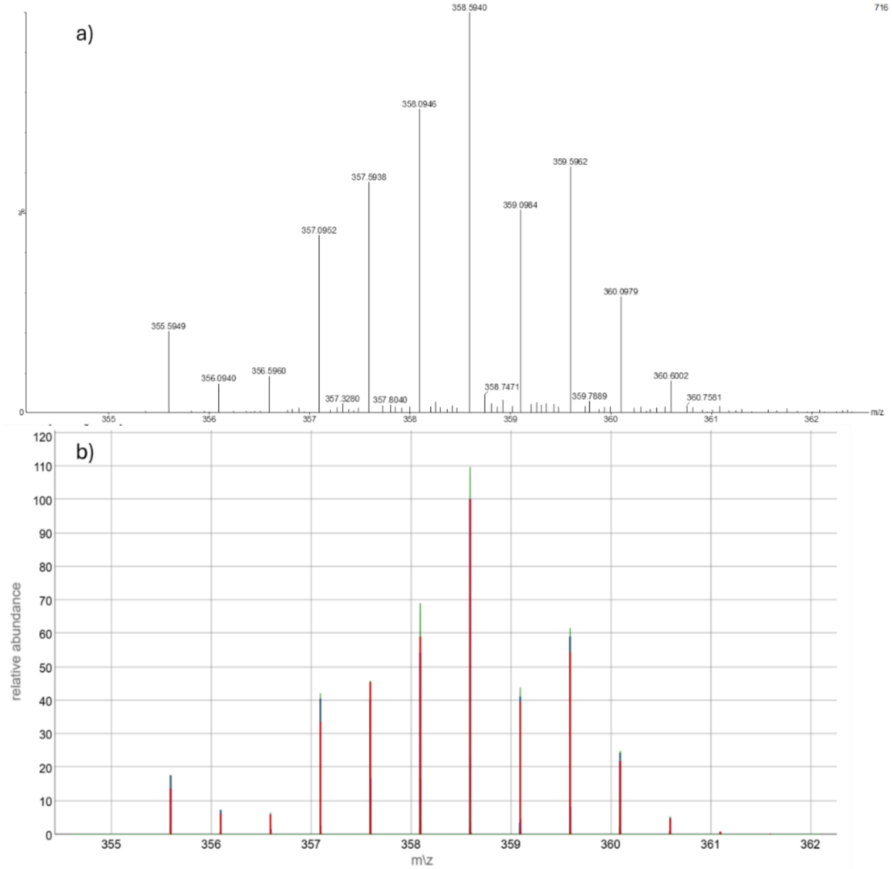

Figure S 32. Expanded a) experimental and b) theoretical ESI-HRMS spectrum of Complex **1** (positive detection mode).

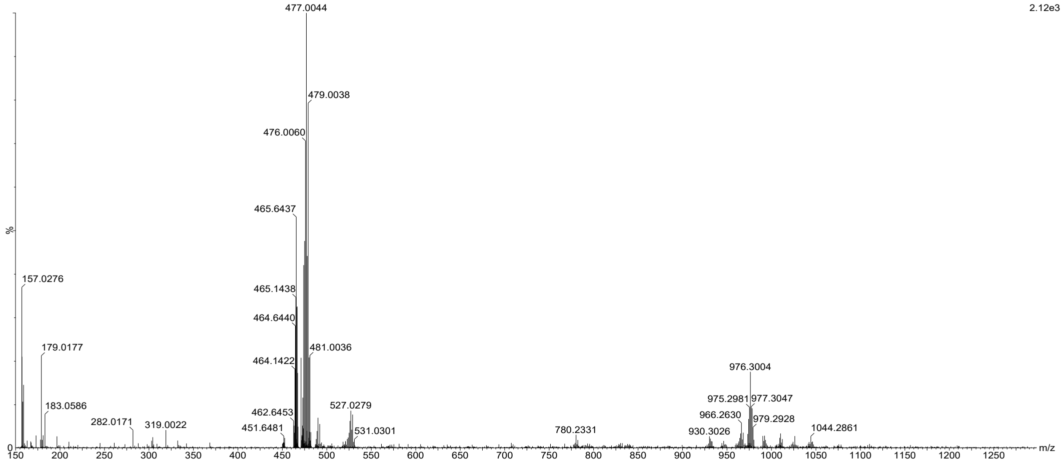

Figure S 33. TOF-ESI-MS spectrum for Complex **2** (positive detection mode).

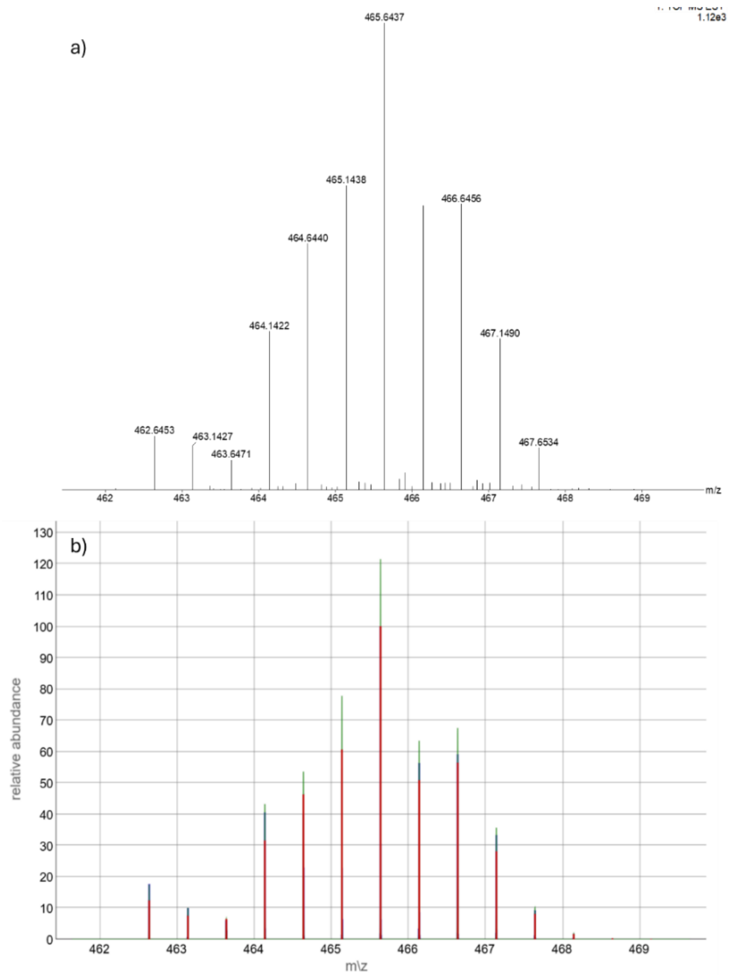

Figure S 34. Expanded a) experimental and b) theoretical ESI-HRMS spectrum of Complex **2** (positive detection mode).

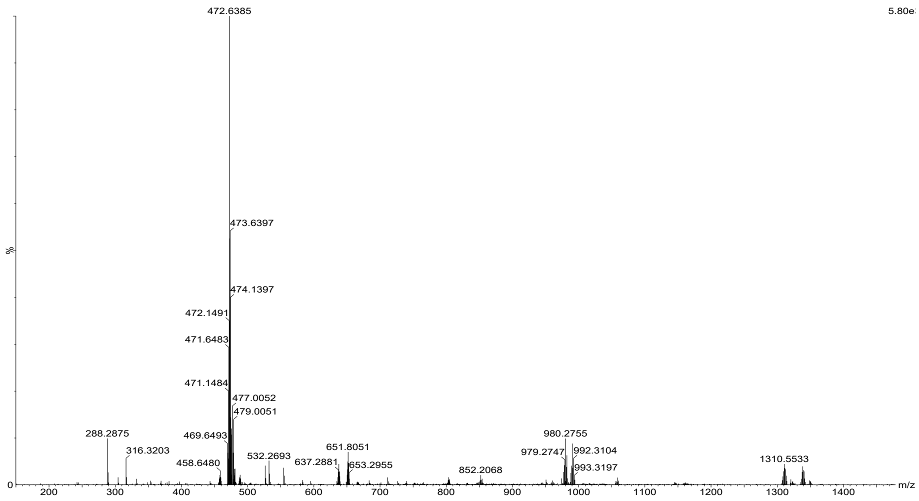

Figure S 35. TOF ESI-MS spectrum for Complex **3** (positive detection mode).

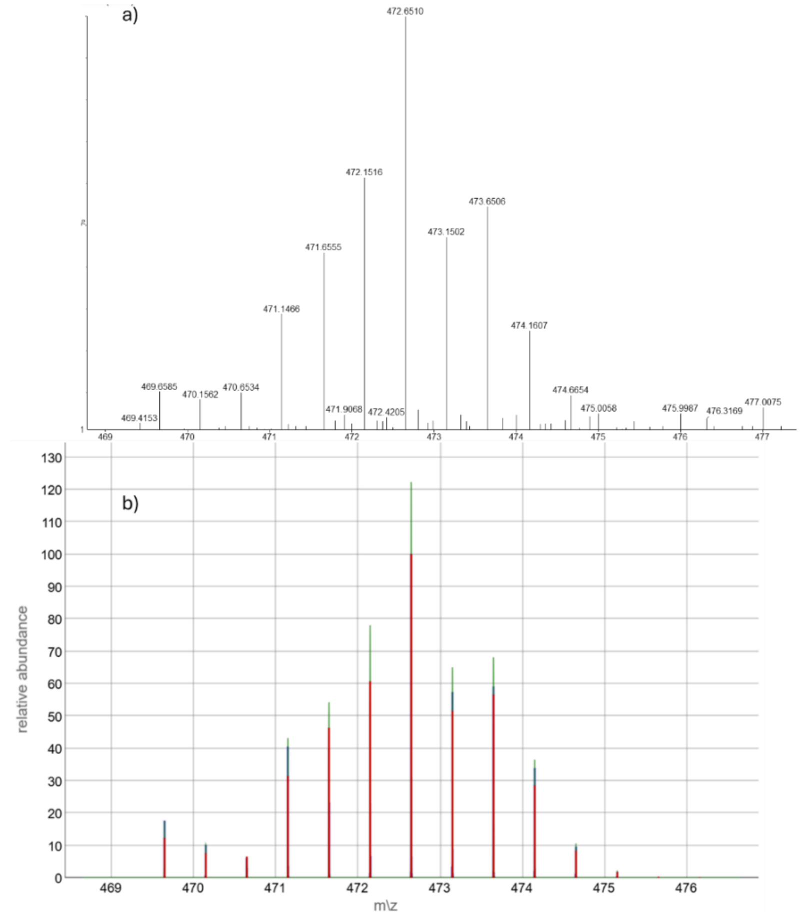

Figure S 36. Expanded a) experimental and b) theoretical ESI-HRMS spectrum of Complex **3** (positive detection mode).

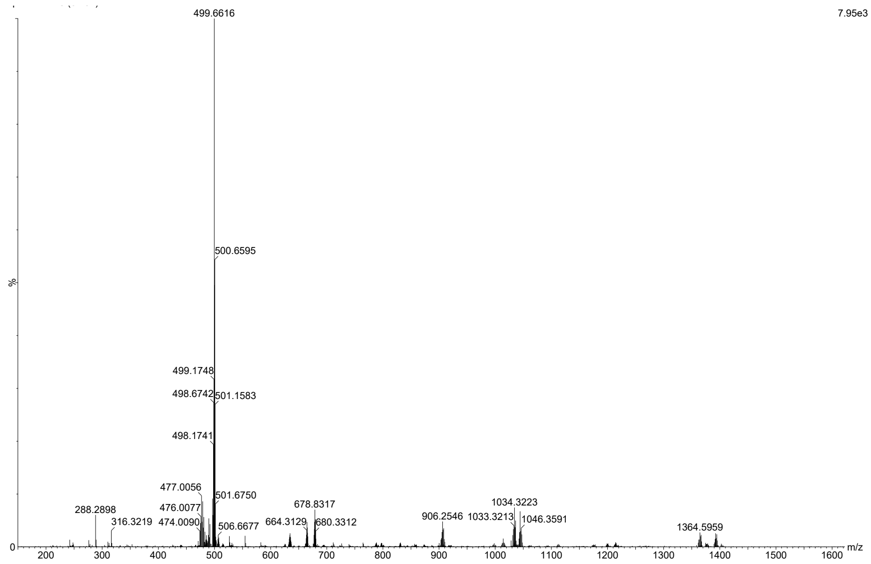

Figure S 37. TOF ESI-MS spectrum for Complex **4** (positive detection mode).

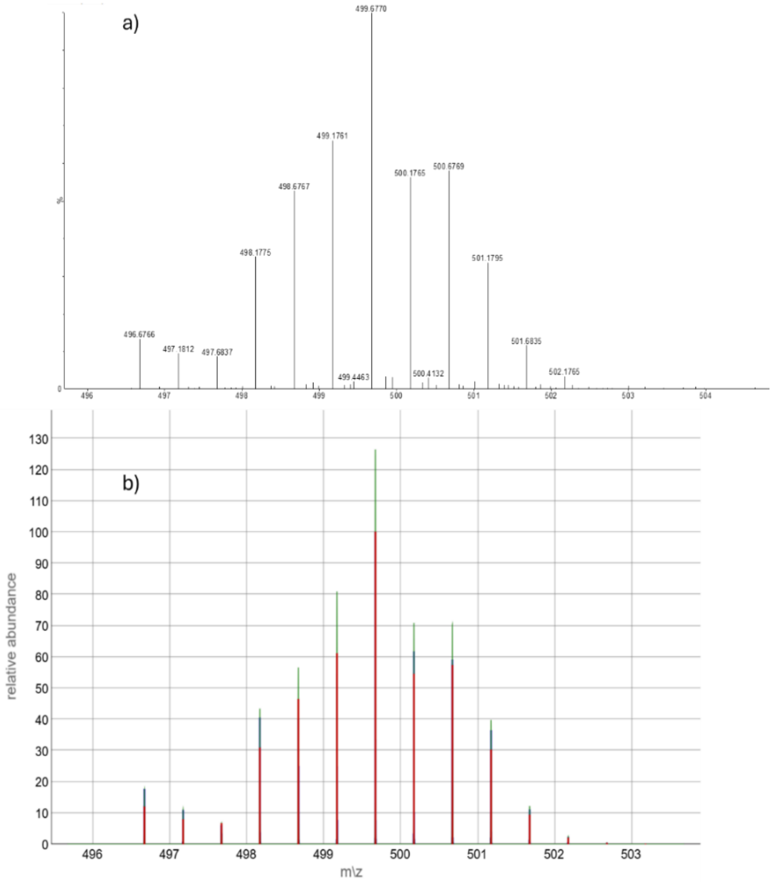

Figure S 38. Expanded a) experimental and b) theoretical ESI-HRMS spectrum of Complex **4** (positive detection mode).

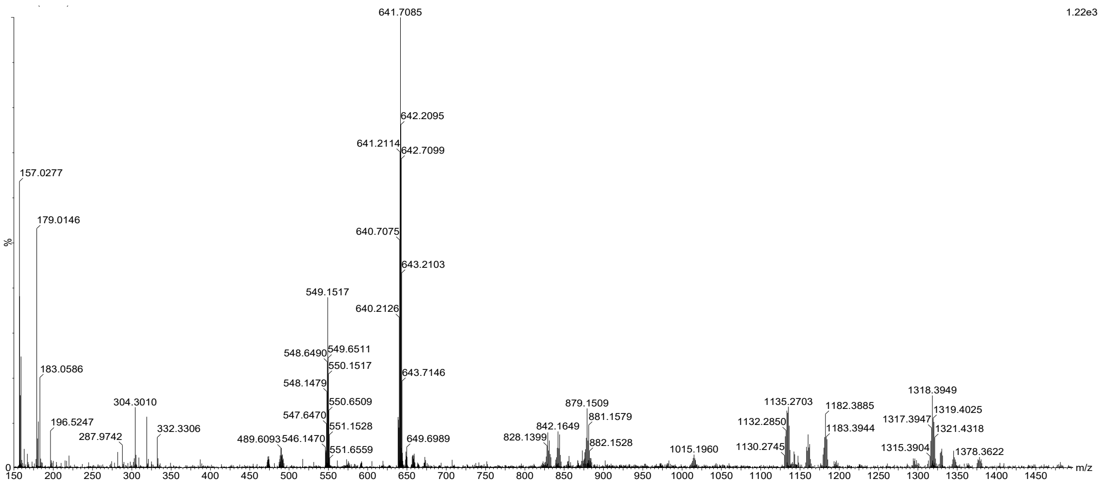

Figure S 39. TOF ESI-MS spectrum for Complex **5** (positive detection mode).

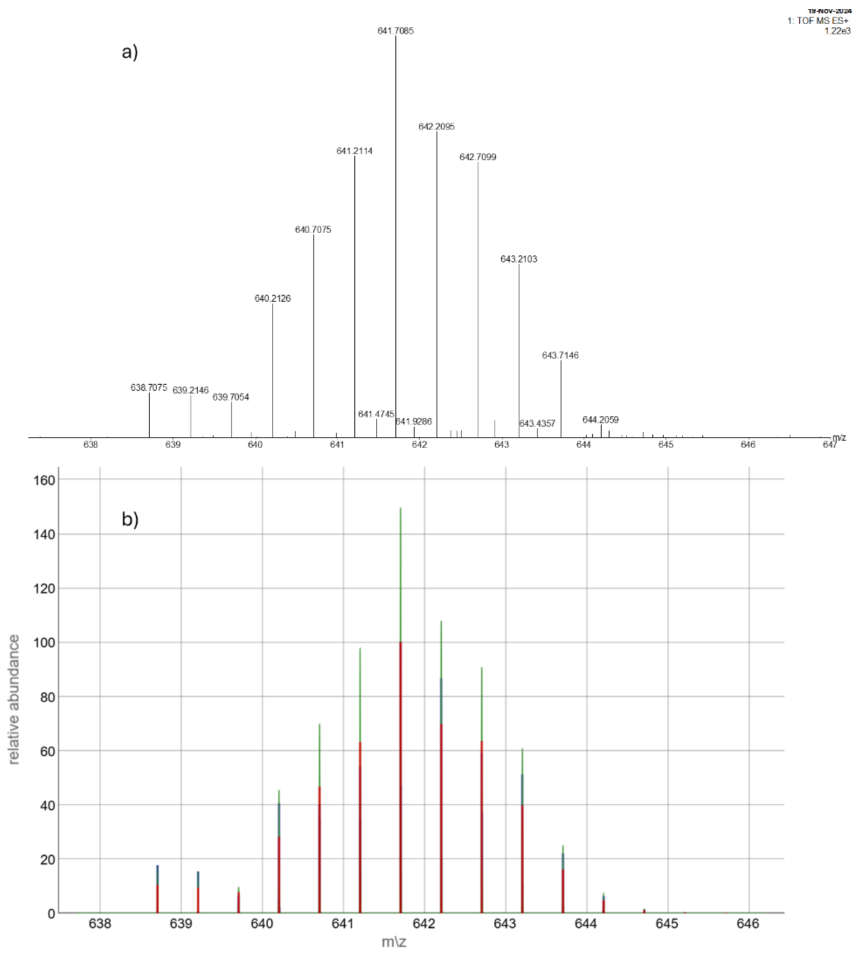

Figure S 40. Expanded a) experimental and b) theoretical ESI-HRMS spectrum of Complex **5** (positive detection mode).

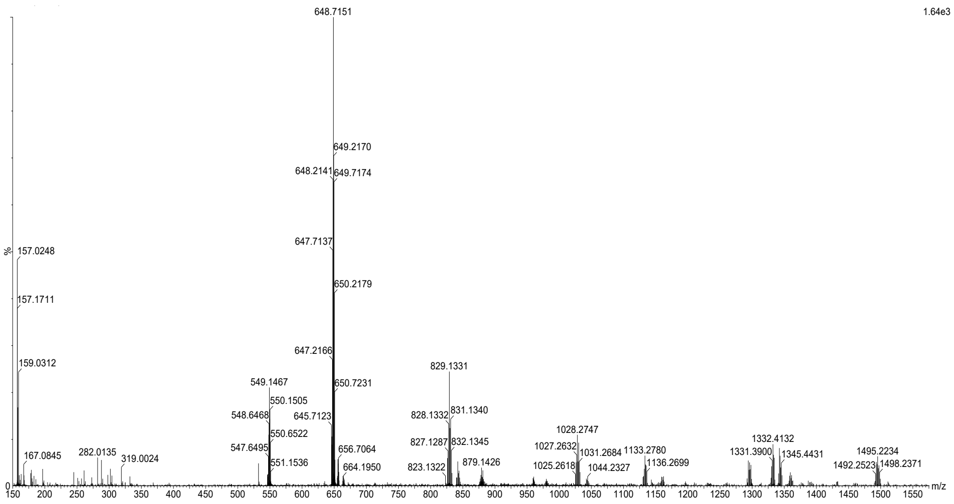

Figure S 41. TOF ESI-MS spectrum for Complex **6** (positive detection mode).

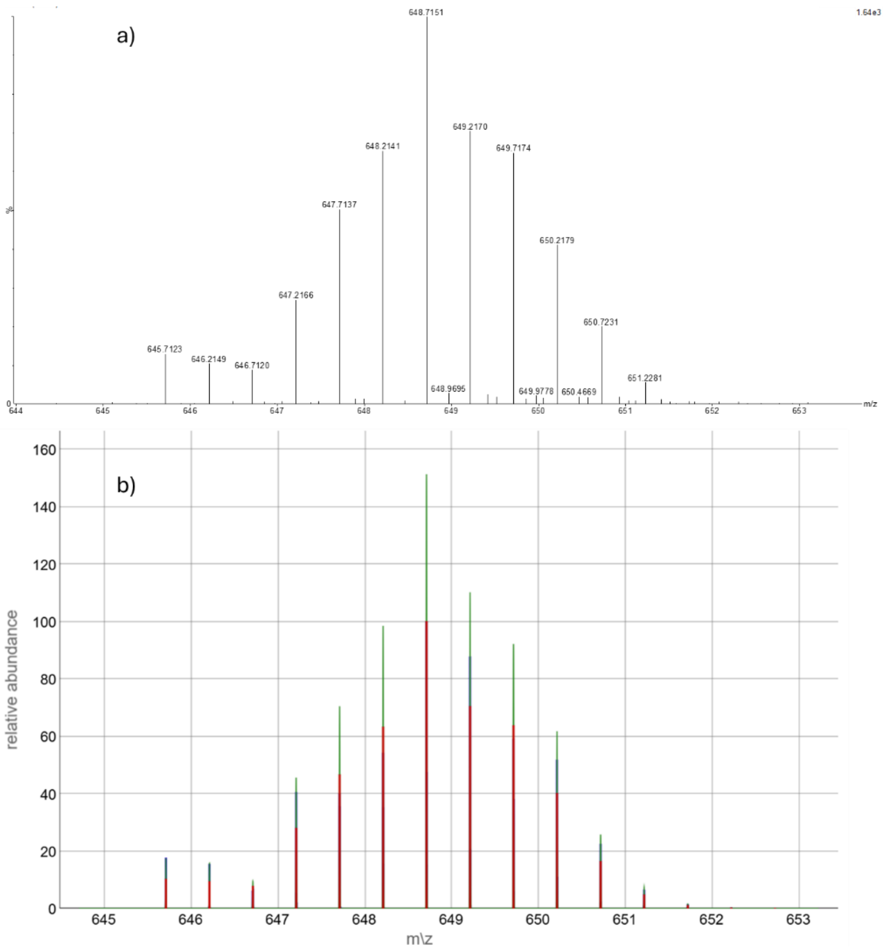

Figure S 42. . Expanded a) experimental and b) theoretical ESI-HRMS spectrum of Complex **6** (positive detection mode).

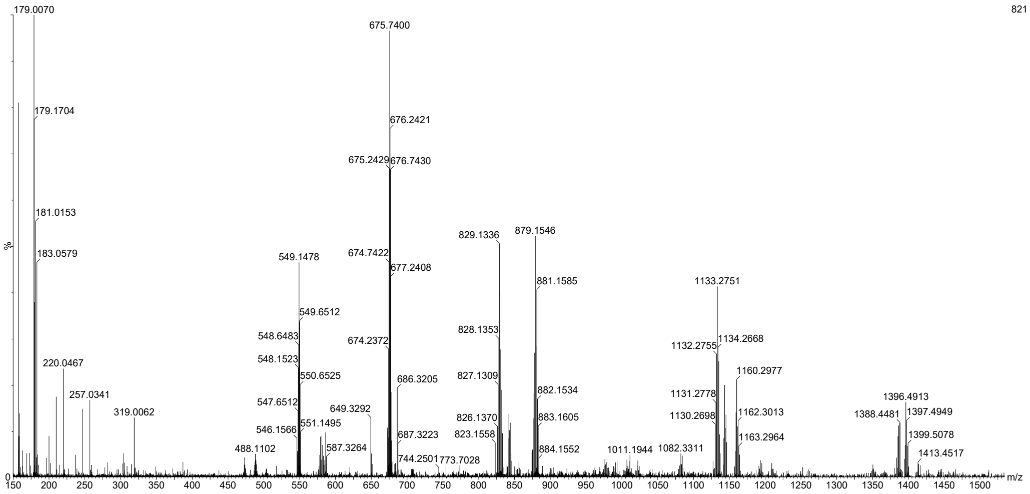

Figure S 43. TOF ESI-MS spectrum for Complex **7** (positive detection mode).

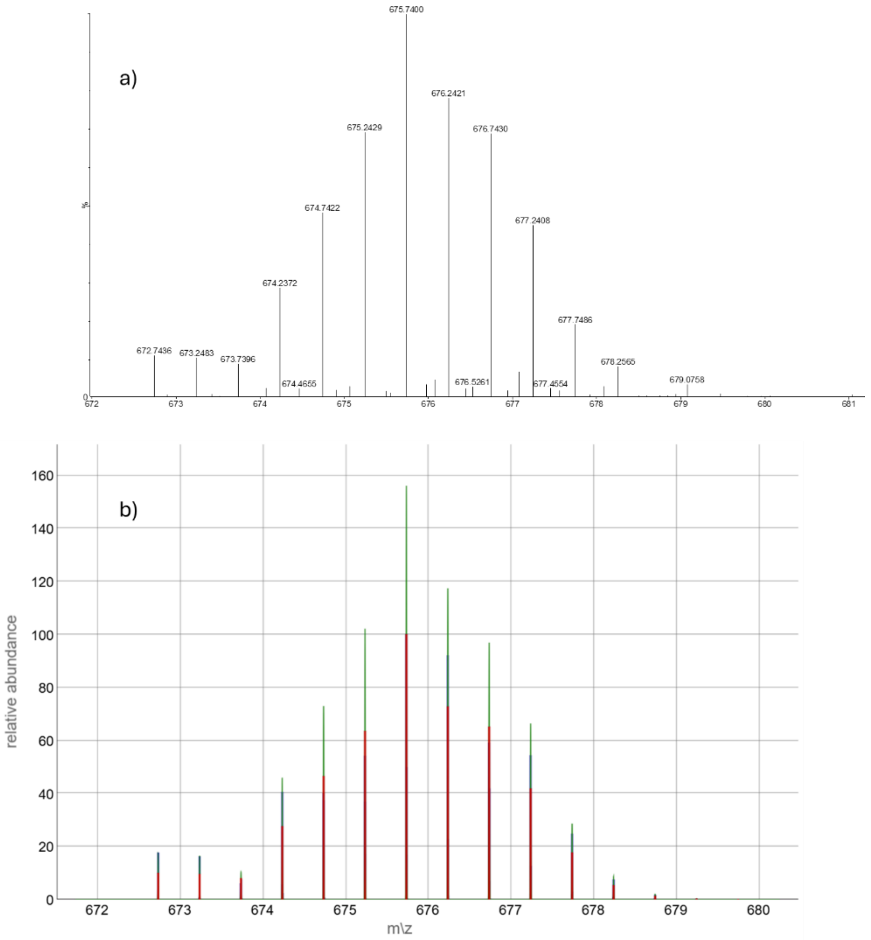

Figure S 44. Expanded a) experimental and b) theoretical ESI-HRMS spectrum of Complex **7** (positive detection mode).

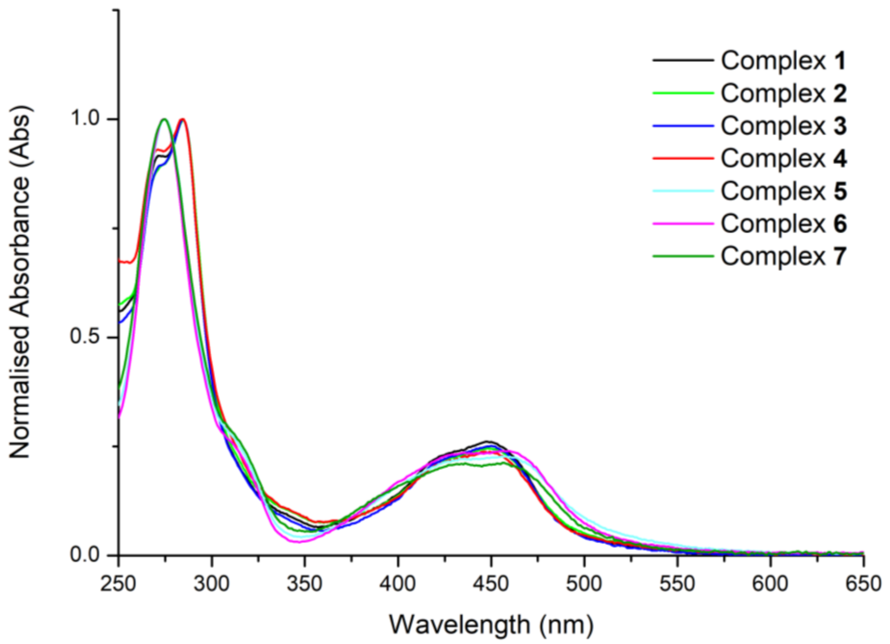

Figure S 45. Absorption spectra of complexes **1**—**7** in MeCN.

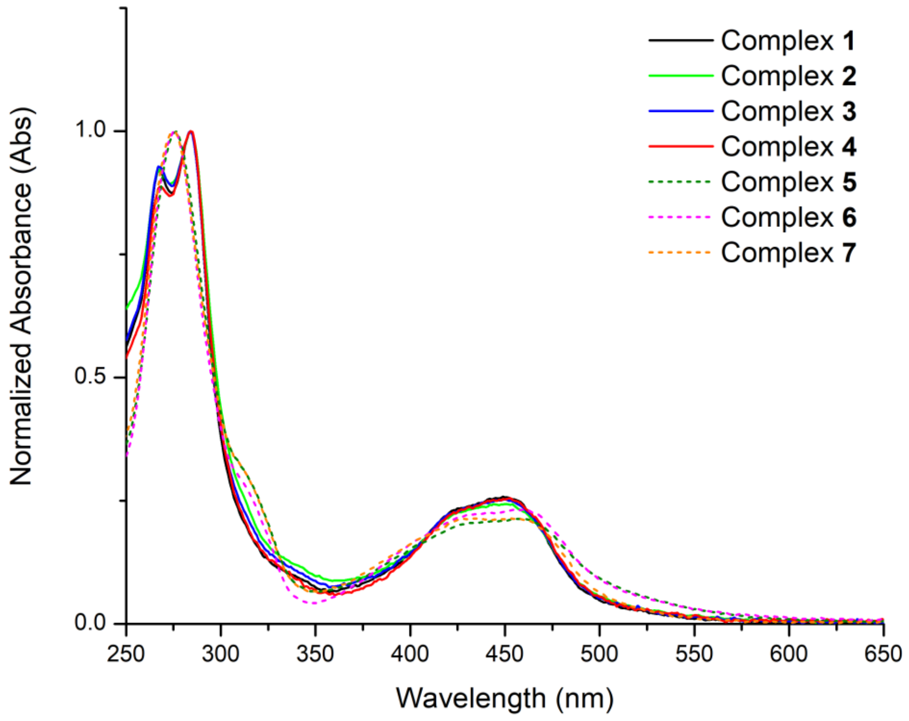

Figure S 46. Absorption spectra of complexes **1**—**7** in Tris-HCl buffer (solid lines) and MeOH (dashed lines).

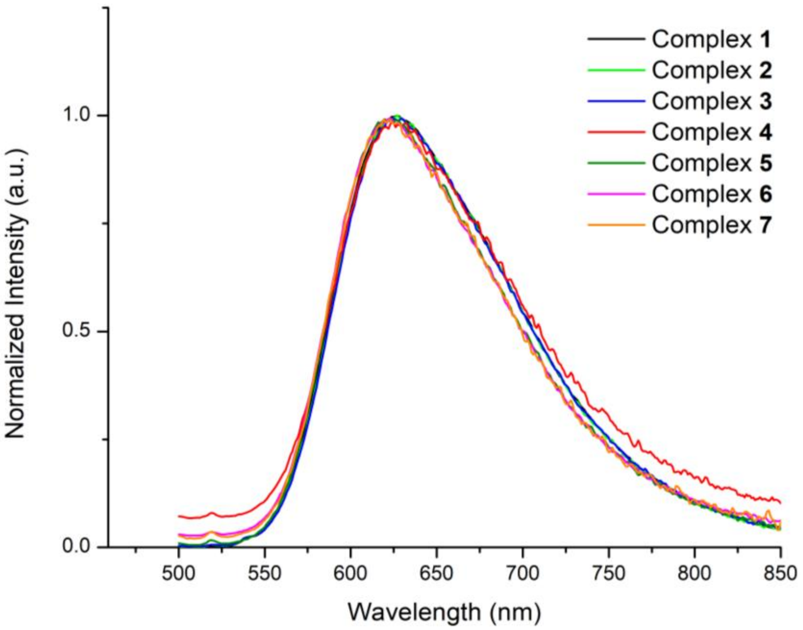

Figure S 47. Emission spectra of complexes **1**—**7** in MeCN; excitation wavelength = 450 nm

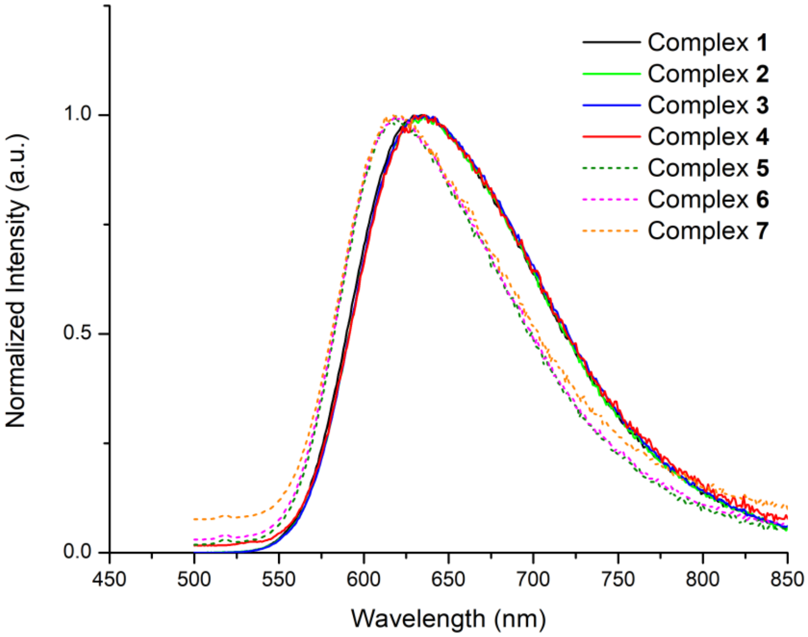

Figure S 48. Emission spectra of complexes **1**—**7** in Tris-HCl buffer (solid lines) and MeOH (dashed lines); excitation wavelength = 450 nm.

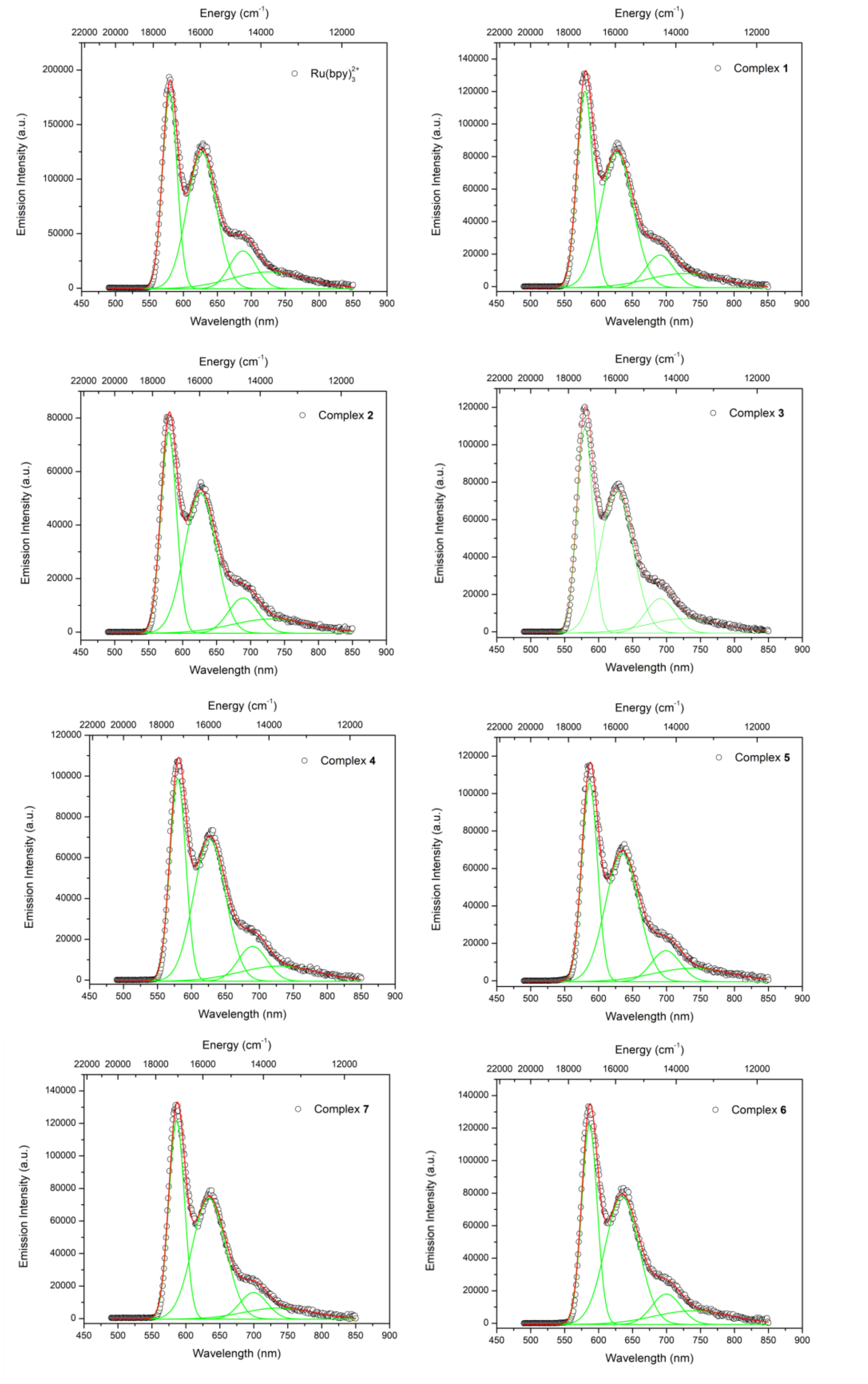

Figure S 49. Low temperature emission spectra of complexes **1**—**7** EtOH/MeOH (4:1 v/v); excitation wavelength = 450 nm.

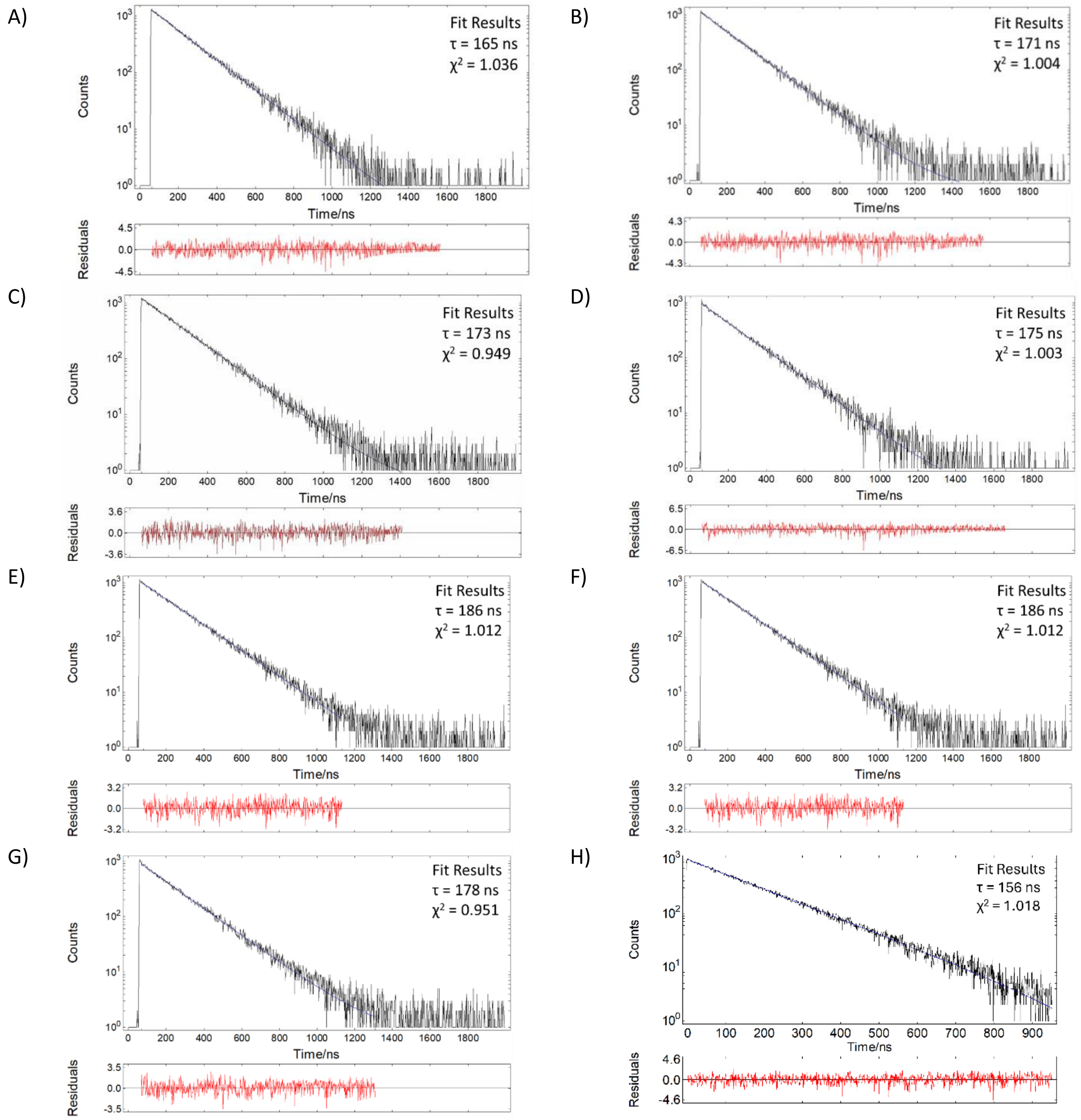

Figure S 50. Experimental decay data and exponential fittings in MeCN for A) Complex **1**, B) Complex **2**, C) Complex **3**, D) Complex **4**, E) Complex **5**, F) Complex **6**, G) Complex **7**, H) $[Ru(bpy)_3]Cl_2$.

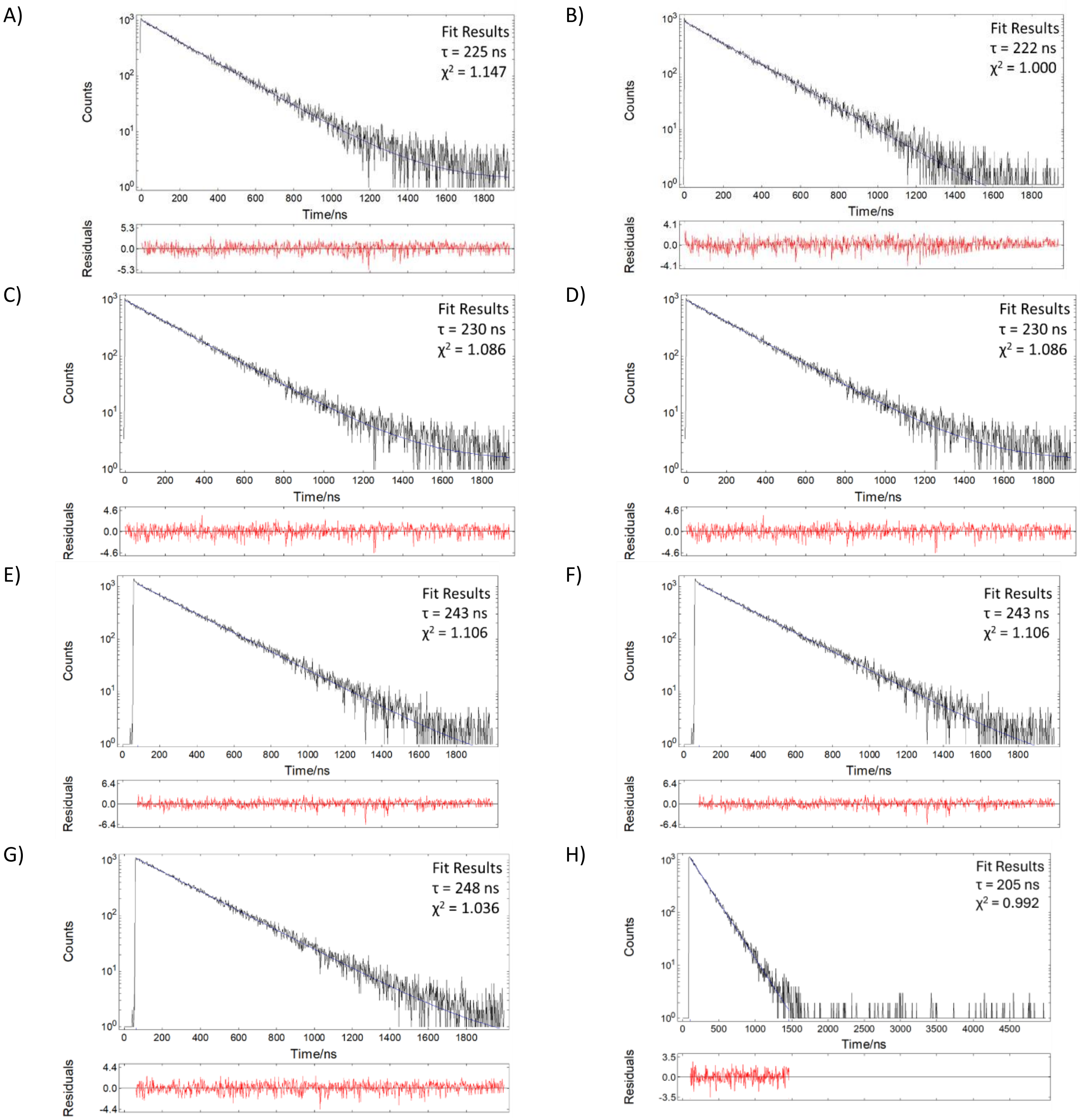

Figure S 51. Experimental decay data and exponential fittings in MeOH for A) Complex **1**, B) Complex **2**, C) Complex **3**, D) Complex **4**, E) Complex **5**, F) Complex **6**, G) Complex **7**, H) $[Ru(bpy)_3]Cl_2$.

A)

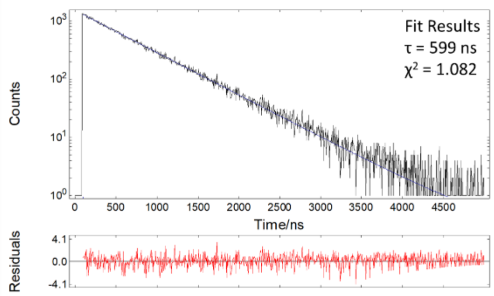
Fit Results
τ = 599 ns
χ^2 = 1.082
Counts
Time/ns
Residuals

B)

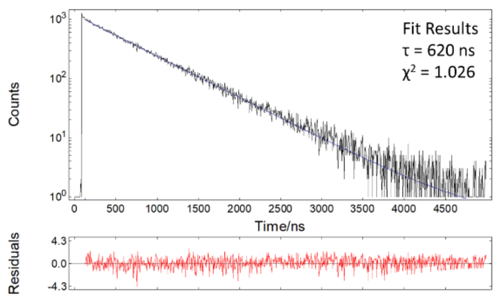
Fit Results
τ = 620 ns
χ^2 = 1.026
Counts
Time/ns
Residuals

C)

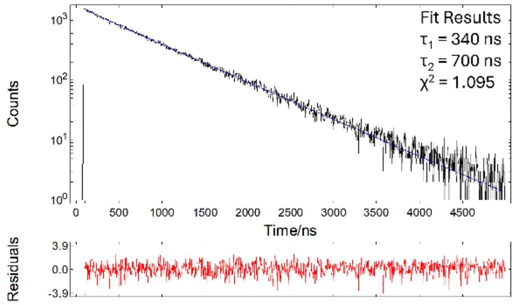
Fit Results
τ_1 = 340 ns
τ_2 = 700 ns
χ^2 = 1.095
Counts
Time/ns
Residuals

D)

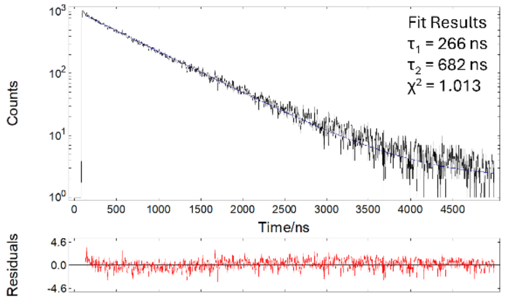
Fit Results
τ_1 = 266 ns
τ_2 = 682 ns
χ^2 = 1.013
Counts
Time/ns
Residuals

E)

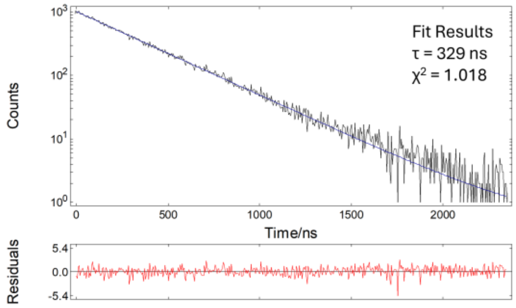
Fit Results
τ = 329 ns
χ^2 = 1.018
Counts
Time/ns
Residuals

F)

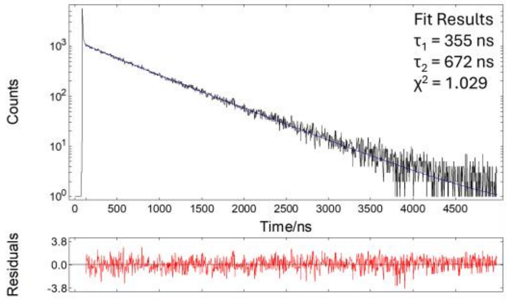
Fit Results
τ_1 = 355 ns
τ_2 = 672 ns
χ^2 = 1.029
Counts
Time/ns
Residuals

G)

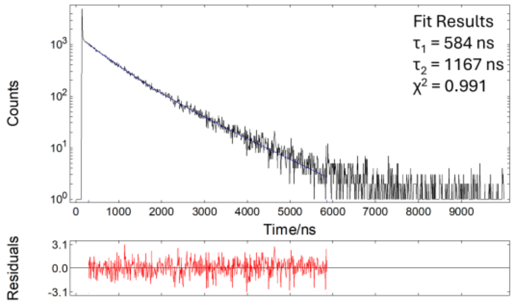
Fit Results
τ_1 = 584 ns
τ_2 = 1167 ns
χ^2 = 0.991
Counts
Time/ns
Residuals

H)

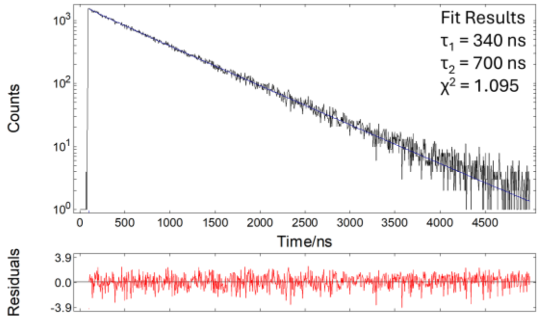
Fit Results
τ_1 = 340 ns
τ_2 = 700 ns
χ^2 = 1.095
Counts
Time/ns
Residuals

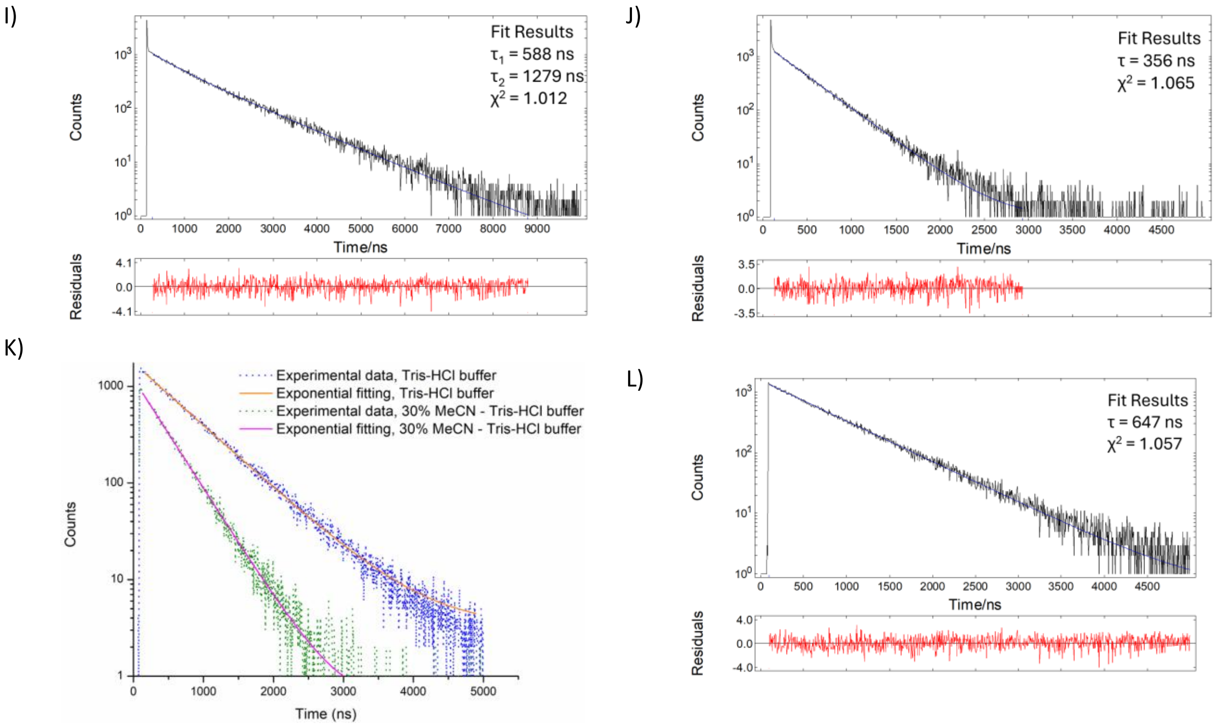

Figure S 52. Experimental decay data and exponential fittings in Tris-HCl buffer for A) Complex **1**, B) Complex **2**, C) Complex **3**, D) Complex **4**, E) $[Ru(bpy)_3]Cl_2$. Exponential decay data and exponential fittings in Tris-HCl buffer for F) Complex **1**, G) Complex **2**, H) Complex **3**, I) Complex **4**, J) $[Ru(bpy)_3]Cl_2$. K) Experimental decay data and exponential fittings of complex **3** in pure Tris-HCl buffer and in 30% MeCN –Tris-HCl buffer. The emission lifetime in 30% MeCN –Tris-HCl buffer is visibly smaller in comparison to pure Tris-HCl buffer. L) Exponential decay data and exponential fittings in Tris-HCl buffer for complex **3** (13 µM)

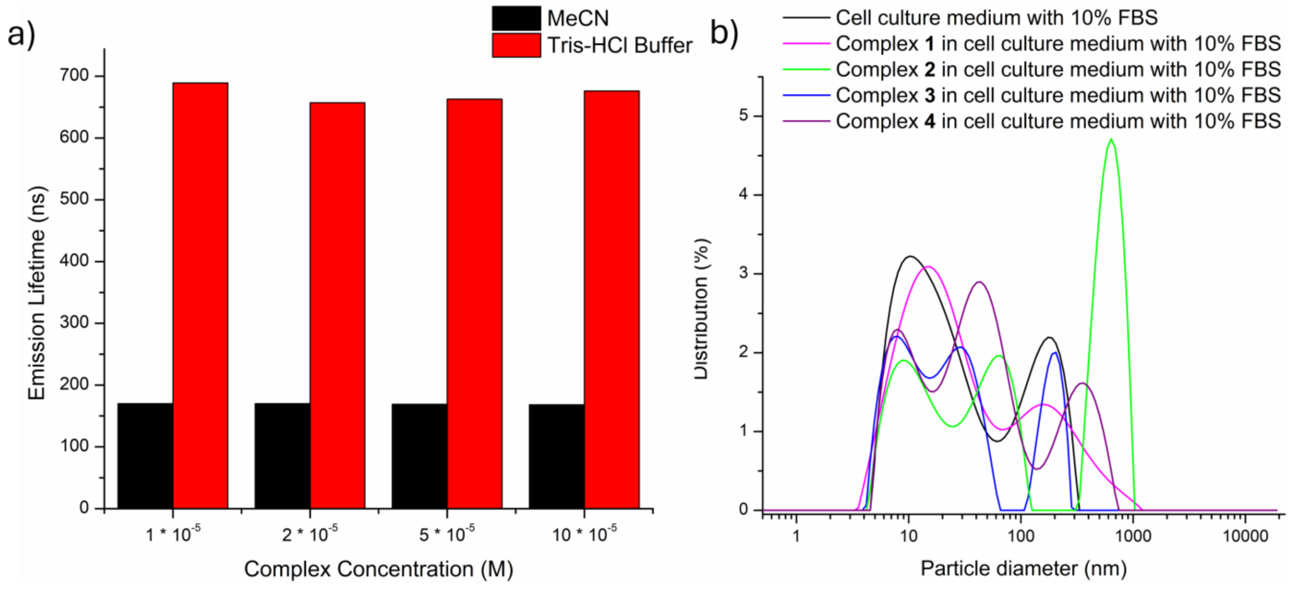

Figure S 53. a) Measured emission lifetime for complex **3** in MeCN and Tris-HCl buffer using different complex concentrations. b) DLS data: particle size distribution by the intensity for complexes **1**–**4** (20 µM) in cell culture medium containing 10% FBS.

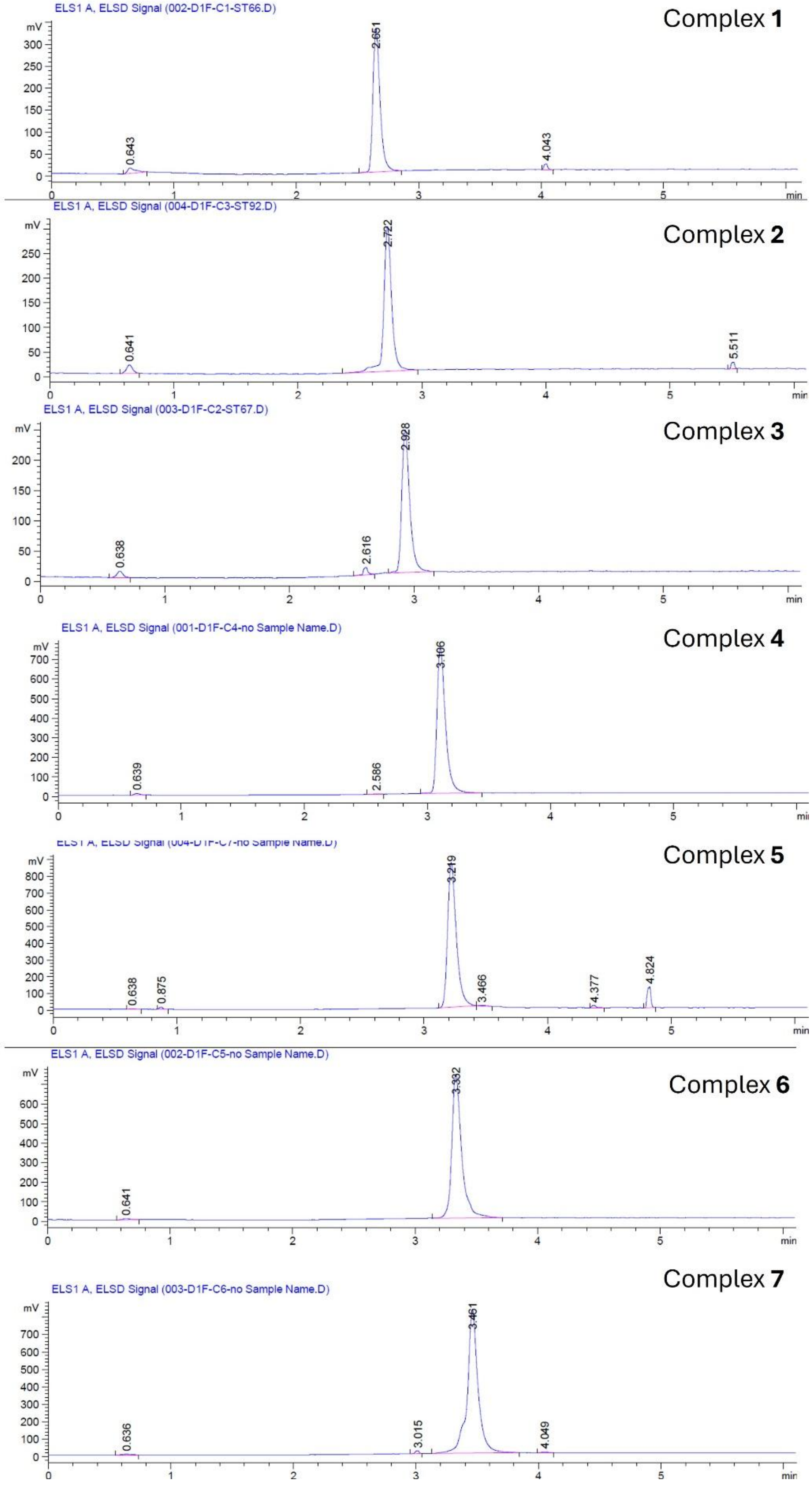

Figure S 54. LC data for Complexes **1**—**7**.

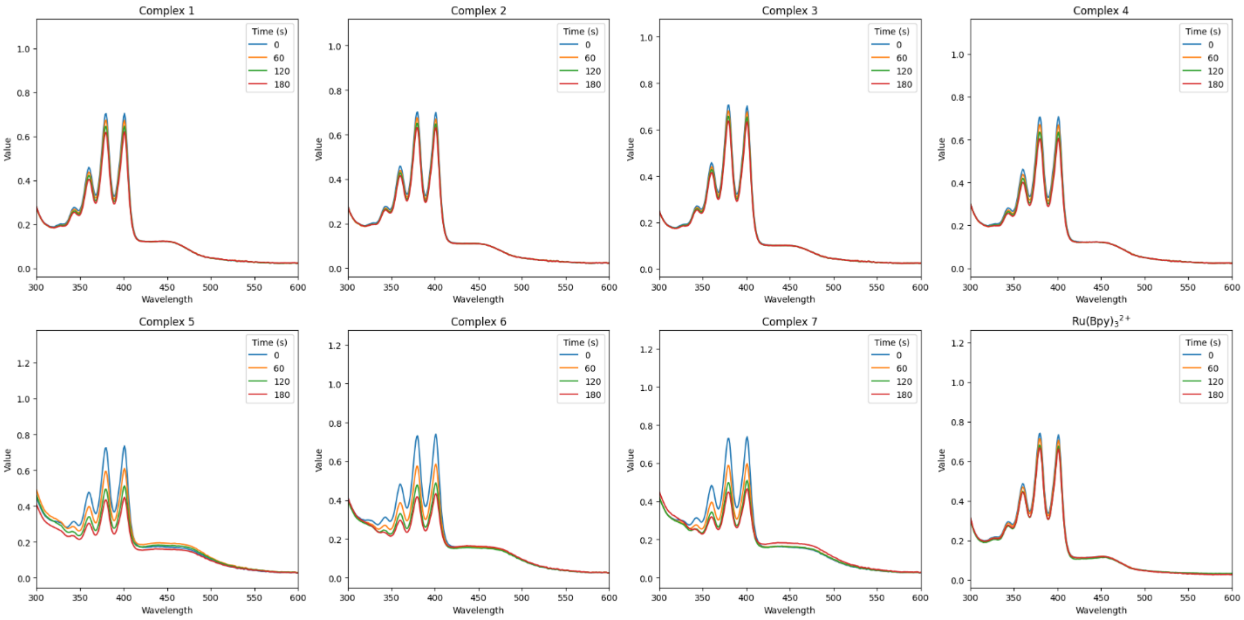

Figure S *55*. Plots showing the decrease in absorbance of ABDA for each complex

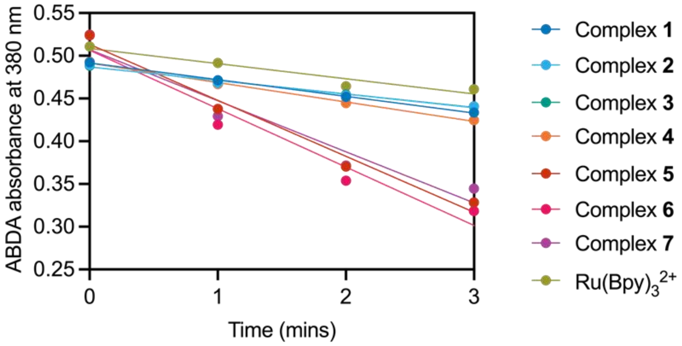

Figure S 56. The change over time in ABDA absorbance (380 nm) fitted to a linear regression for complexes **1**-**7** and [Ru(bpy)3]$^{2+}$.

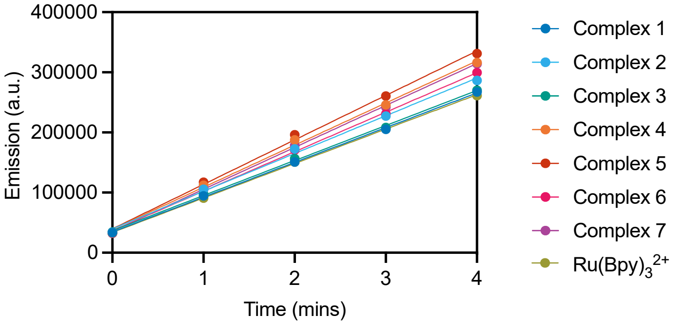

Figure S 57. The change over time in DHR-123 emission (535 nm) fitted to a linear regression for complexes **1**-**7** and [Ru(bpy)3]$^{2+}$.

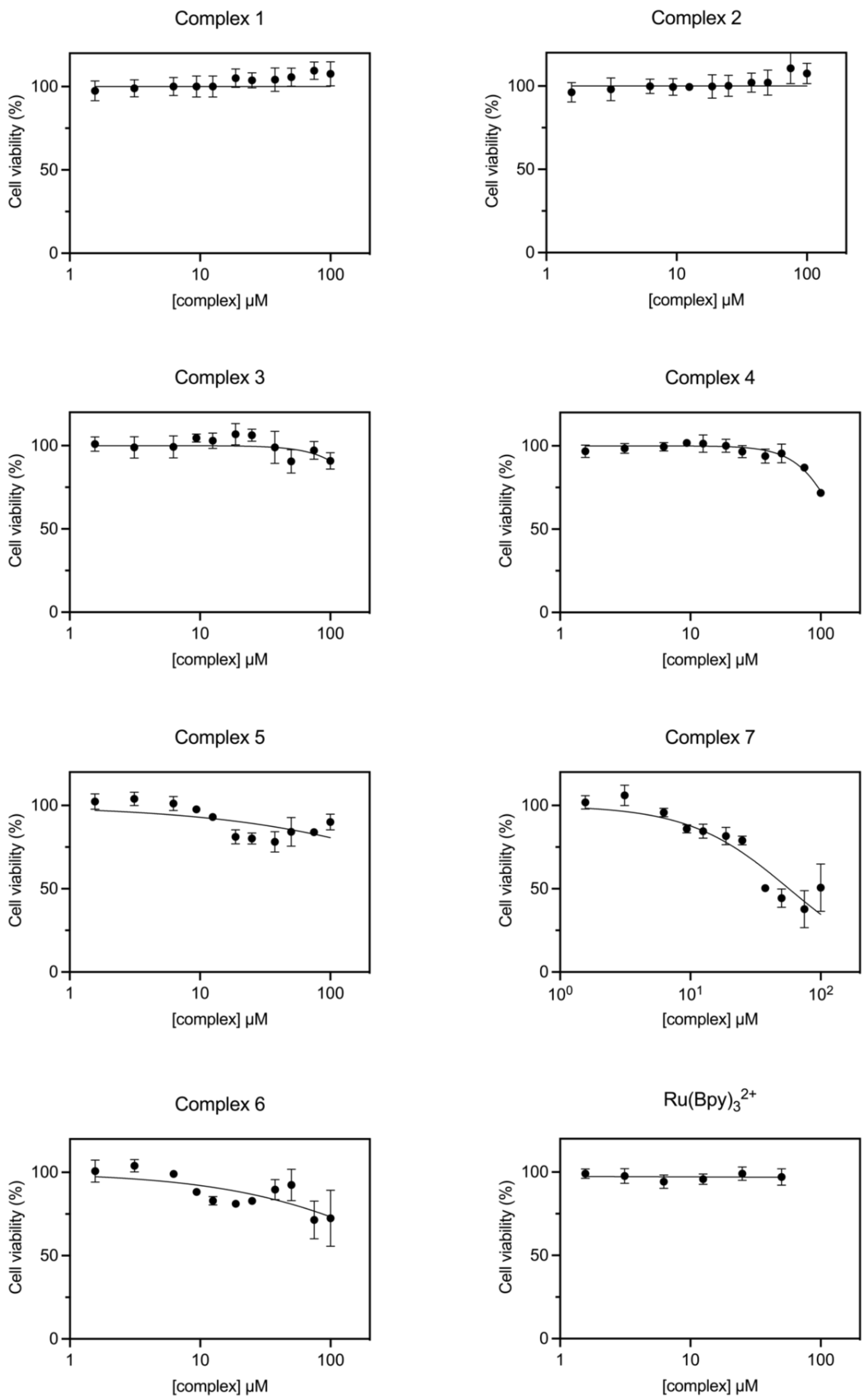

Figure S 58. Plotted cytotoxicity results for Complexes **1**-**7** and $Ru(bpy)_3^{2+}$ in MCF-7 cells in the dark.

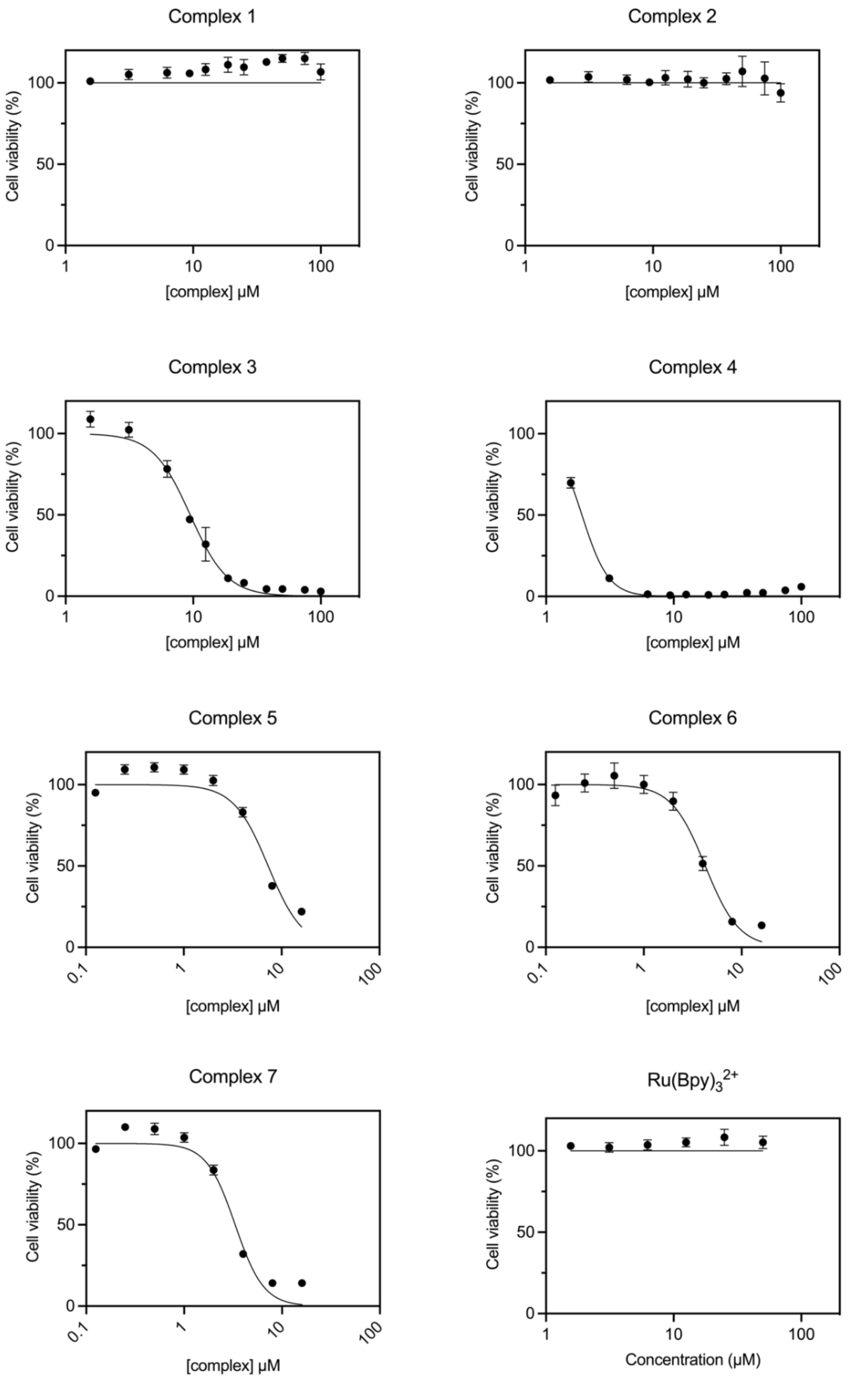

Figure S 59. Plotted cytotoxicity results for Complexes **1**-**7** and $Ru(bpy)_3^{2+}$ in MCF-7 cells upon irradiation (457 nm).

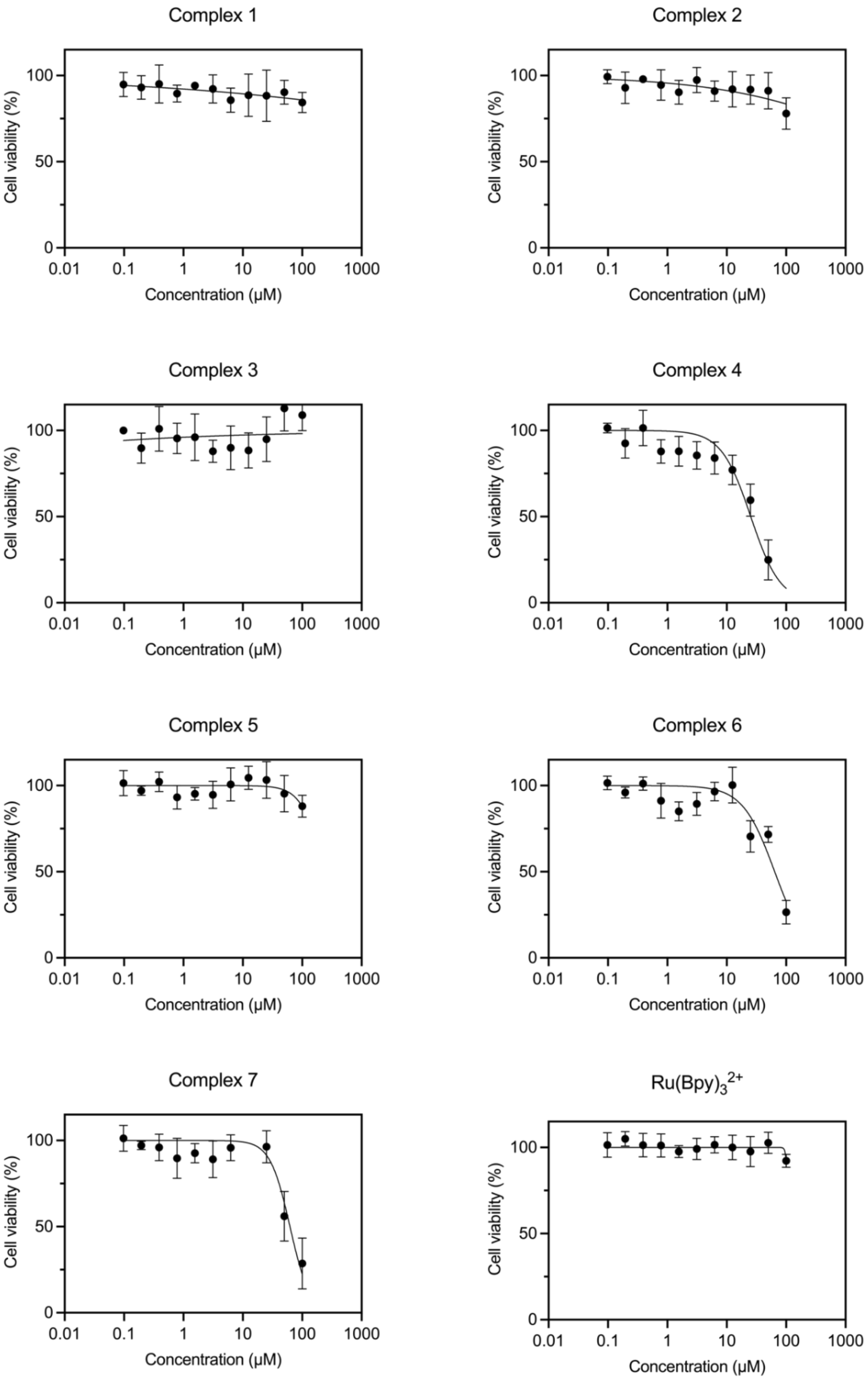

Figure S 60. Plotted cytotoxicity results for Complexes **1**-**7** and $Ru(bpy)_3^{2+}$ in MDA-MB-453 cells in the dark.

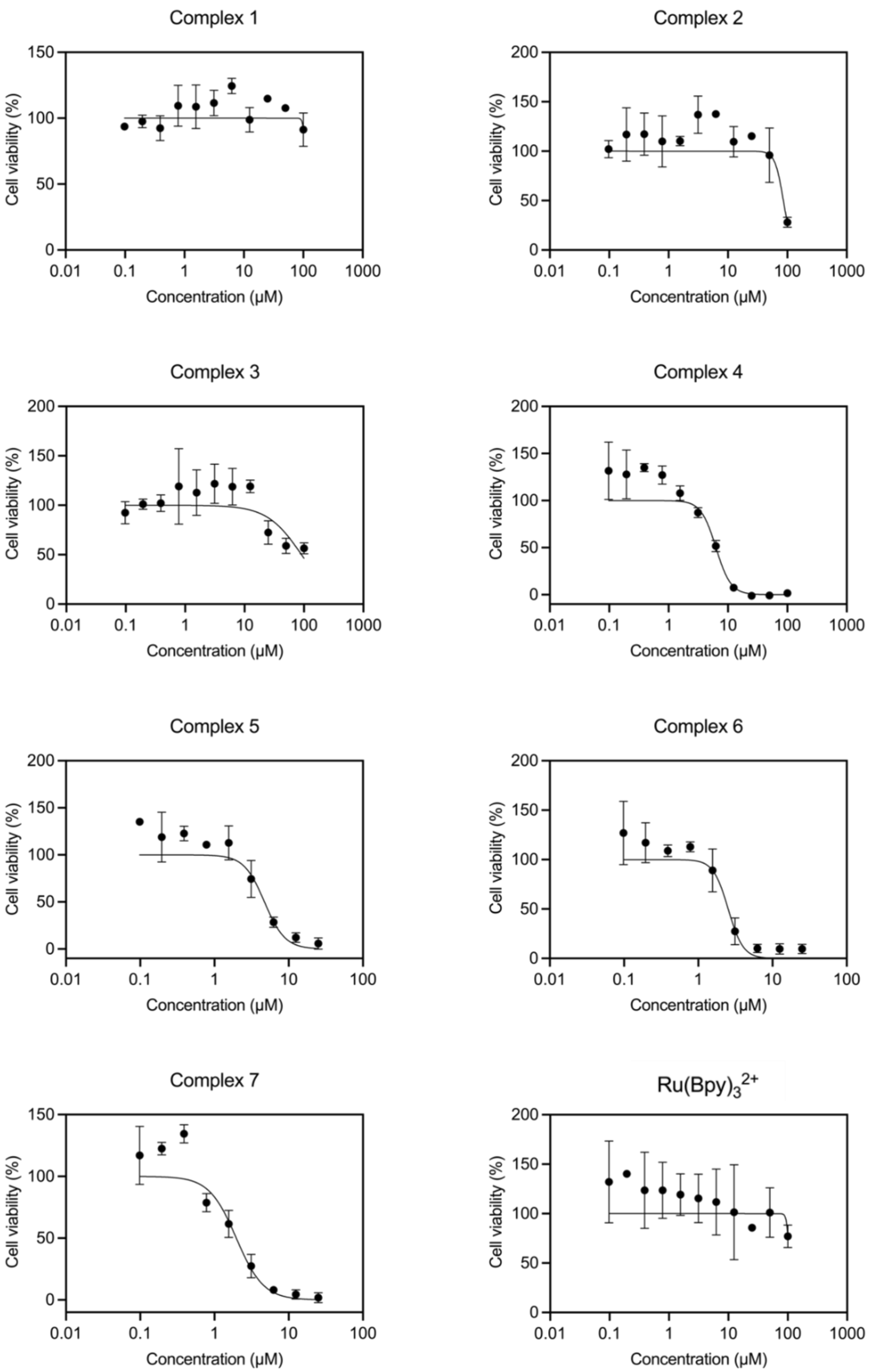

Figure S 61. Plotted cytotoxicity results for Complexes **1**-**7** and $Ru(bpy)_3^{2+}$ in MDA-MB-453 cells upon irradiation (457 nm).

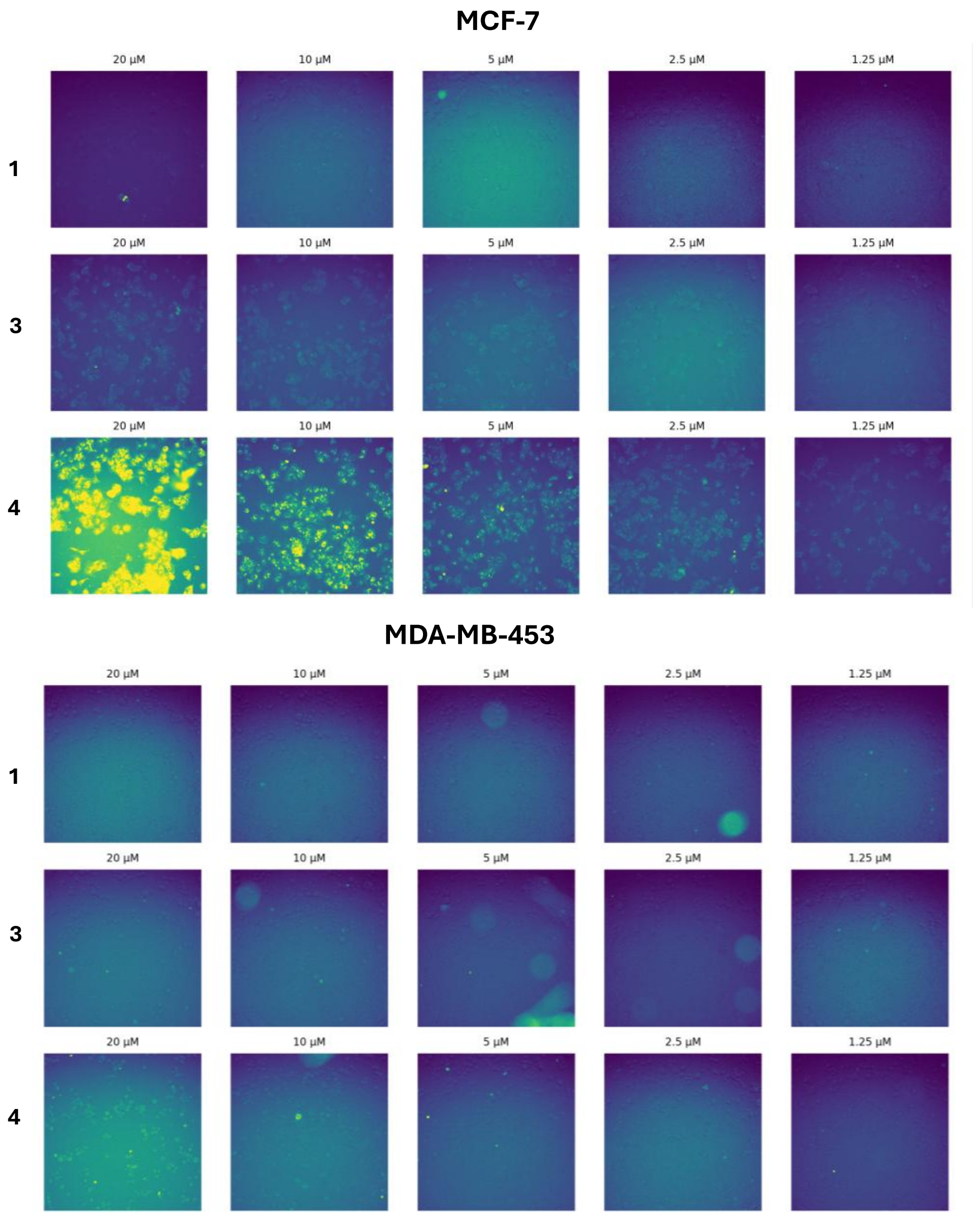

Figure S 62. Microscopy imaging of cells incubated with different concentrations of complexes **1**, **3** and **4** for 6 hours.
